# CRISPR-editing of the virus vector *Aedes albopictus* cell line C6/36, illustrated by prohibitin 2 gene knockout

**DOI:** 10.1016/j.mex.2024.102817

**Published:** 2024-06-21

**Authors:** Shiu-Wan Chan

**Affiliations:** Faculty of Biology, Medicine and Health, School of Biological Sciences, The University of Manchester, Michael Smith Building, Oxford Road, Manchester M13 9PT, United Kingdom

**Keywords:** CRISPR, Prohibitin 2, PHB2, *Aedes albopictus*, Mosquitoes, C6/36, Virus vector, Vector-borne diseases, CRISPR-editing of mosquito cells

## Abstract

*Aedes* mosquitoes are important virus vectors. We provide a toolkit for CRISPR-Cas9-editing of difficult-to-knockdown gene previously shown to be refractory to siRNA silencing in mosquito cells, which is pivotal in understanding vector biology, vector competence, host-pathogen interactions and in gene annotations. Starting from database searches of *Ae. albopictus* and the C6/36 cell line whole genome shotgun sequences for the prohibitin 2 (PHB2) gene, primers were designed to confirm the gene sequence in our laboratory-passaged C6/36 cell line for the correct design and cloning of CRISPR RNA into an insect plasmid vector to create a single guide RNA for the PHB2 gene target. After transfection of this plasmid vector into the C6/36 cells, cell clones selected by puromycin and/or limiting dilution were analyzed for insertions and deletions (INDELs) using PCR, sequencing and computational sequence decomposition. From this, we have identified mono-allelic and bi-allelic knockout cell clones. Using a mono-allelic knockout cell clone as an example, we characterized its INDELs by molecular cloning and computational analysis. Importantly, mono-allelic knockout was sufficient to reduce >80 % of PHB2 expression, which led to phenotypic switching and the propensity to form foci but was insufficient to affect growth rate or to inhibit Zika virus infection.

• We provide a toolkit for CRISPR-Cas9-editing of the virus vector, *Aedes albopictus* C6/36 cell line
• We validate this using a difficult-to-knockdown gene prohibitin 2
• This toolkit is pivotal in understanding vector biology, vector competence, host-pathogen interactions and in gene annotations

## Graphical abstract

**Figure:**
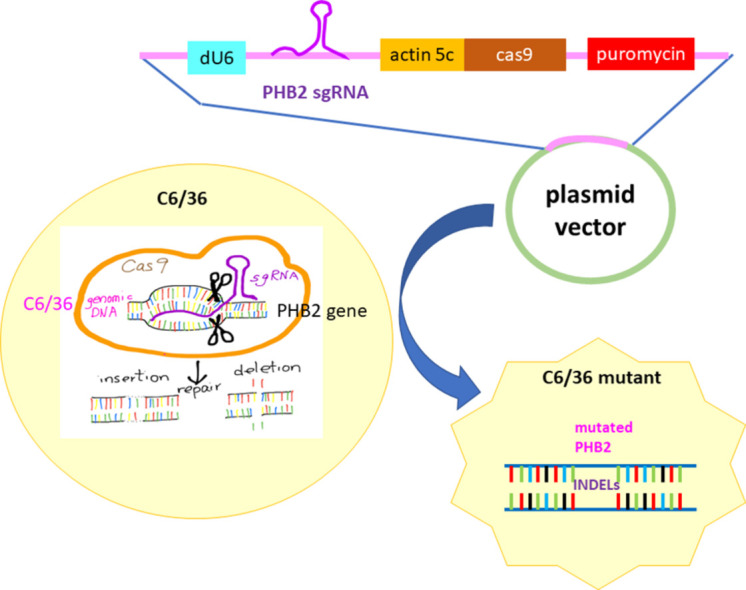

Specifications table

**Table:** 

Subject area:	Immunology and Microbiology
More specific subject area:	*Virology*
Name of your method:	*CRISPR-editing of mosquito cells*
Name and reference of original method:	*CRISPR*
Resource availability:	*Addgene; OFFinder; TIDE, CRISPR-ID* Addgene: pAc-sgRNA-Cas9 http://www.rgenome.net http://tide.nki.nl http://crispid.gbiomed.kuleuven.be

## Background

*Aedes* mosquitoes are important vectors for the transmission of dengue (DENV), Zika (ZIKV), yellow fever and chikungunya (CHIKV) viruses. Traditional chemical control is threatened by its potential impact on wildlife and environment and the emergence of insecticide-resistant mosquitoes [1]. Genetic intervention has become a new strategy in vector control. *Anopheles* and *Aedes* mosquitoes have been successfully engineered to carry pathogen-resistant transgenes or to produce suicide progeny, sterile female and male and male-biased population [[2], [3], [4], [5], [6], [7]].

Fundamental study in vector biology has played an important role in shaping designer mosquitoes. The surge in mosquito genomic sequencing data also requires a parallel increase in speed in functional annotations of genes. When insectaries are not generally available, the use of mosquito cell lines has greatly facilitated mutant library screening and functional study to unravel knowledge on vector biology, vector competence and virus-mosquito host interactions in order to find new targets for blocking transmission and in gene annotations.

Methodologies and resources for CRISPR-editing in mosquito cell lines are limited. Only until recently several methodologies have been developed using reporter genes [8] or genomically integrated reporter genes [9] and native genes from *An. coluzzii* cell line Sua-5B [9], *Ae. aegypti* cell line Aag2 and *Ae. albopictus* cell lines U4.4 [10] and C6/36 [11]. Here, we provide methodologies and extensive bioinformatics analysis for easy and efficient knockout/knockdown of native genes in the *Ae. albopictus* cell line C6/36 even without the use of homologous promoters, antibiotic selection and cell sorting as in the above studies [[8], [9], [10], [11]]. This is a significant improvement over the other methods that relied on the use of additional knock-in of puromycin and selectable fluorescent marker genes for antibiotic selection and cell sorting [11] or engineering of mosquito-specific promoters [9,10] and transfection of multiple single guide RNAs (sgRNAs) [10]. Moreover, our methods have proved to be effective for difficult-to-target inter-dependent genes.

*Aedes* mosquitoes are spreading into new areas; thus increasing the global population at risk of vector-borne diseases [12,13]. Despite *Ae. aegypti* being the principal vector, *Ae. albopictus* is predicted to be the main mosquito species responsible for global spread [12] due to its hardiness in temperate climates and its ability to undergo diapause to overwinter in colder locations [14]. *Ae. albopictus* is already the primary epidemic vector in several outbreaks of DENV, ZIKV and CHIKV [[15], [16], [17]]. Therefore, we chose the *Ae. albopictus* cell line, C6/36, in this study for gene knockout (KO).

Aedine prohibitin 2 (PHB2) has been proposed as a putative receptor for DENV-2 [18]. PHB2 is refractory to siRNA silencing in aedine cell lines. PHB2 knockdown could only be achieved by siRNA silencing of its inter-dependent partner PHB1; therefore, there is a need for a direct genetic knockdown tool for PHB2 in order to dissect the function of this protein. We, therefore, used CRISPR-genome-editing of PHB2 to illustrate that our method could be used in knocking out genes previously refractory to siRNA silencing in mosquito cells.

## Method details

### Database search for the *Aedes albopictus* PHB2 gene sequence

The genome of the Asian tiger mosquito, *Ae. albopictus* has been sequenced [[19], [20], [21], [22]]. A search of the National Centre for Biotechnology Information (NCBI) GenBank ‘genome’ database for ‘*Aedes albopictus*’ returned four whole genome shotgun (WGS) sequences (one Foshan laboratory isolate derived from wild mosquitoes in the Southeastern China: JXUM00000000.1 [19]; one Foshan inbred line FPA with two assemblies: SWKZ00000000.1 [20] & JAFDOQ000000000.1 [21]; one Rimini isolate: LMAV000000000.1 [22] and one C6/36 cell line derived from the larval tissue of *Ae. albopictus*: MNAF00000000.2 [23]).

The Foshan and FPA isolates WGS sequences have been annotated [[19], [20], [21]]. A search of the NCBI GenBank ‘nucleotide’ database for the ‘*Aedes albopictus* PHB2’ identified two loci KQ562192.1 and KQ571446.1 from the Foshan strain WGS sequence and a search for the ‘*Aedes albopictus* prohibitin 2' or ‘*Aedes albopictus* prohibitin’ returned two loci NW_021838943.1 and NW_021837434.1 from the FPA isolate WGS sequence that encodes a hypothetical protein homologous to the mammalian PHB2 protein. In addition, a search for the ‘*Aedes albopictus* prohibitin-2' in the NCBI GenBank nucleotide database returned two sets of transcripts. The first set of transcripts is derived from the transcriptome shotgun assembly (TSA) loci GAPW01002914.1 and GAPW01002921.1 that contains putative PHB2 mRNAs, Aa-54605 and Aa-54604, respectively [24]. The second set of transcripts contains four variants (X1 to X4) from the FPA genomic scaffold NW_021838943.1 with identical amino acid (aa) sequences apart from 3' end ambiguity (SuppFig.S1).

The human PHB2 has three reference isoforms (1, 3 and X1) (http://www.nlm.nhs.gov/gene/?term=phb2). Isoform 1 is the longest with 299aa whereas isoform 3 has a 38aa internal deletion and isoform X1 is truncated (SuppFig.S1). The *Drosophila melanogaster* PHB2 has six transcript variants from A-F (http://www.nlm.nhs.gov/gene/?term=phb2) (FlyBase Gene Report: Dmel\Phb2). Transcript variants A-C are identical in aa sequences to transcript variant F. The human and *Drosophila* PHB2s share 70 % aa identity. As expected, the mosquito PHB2 is more similar to *Drosophila* PHB2 (83 % aa identity) than to human PHB2 (73–74 % aa identity). Amongst the mosquito PHB2 contig sequences, the KQ571446.1 and Aa-54605 sequences encode a 299aa PHB2 which is the same size as the human and *Drosophila* PHB2s whereas the other sequence, KQ562192.1, has extended 3' end; therefore, KQ571446.1 was used as a reference sequence in the following search.

To identify the PHB2 from the C6/36 and Rimini WGS sequence genomes, the Foshan KQ571446.1 PHB2 nucleotide sequence was used as the input sequence to BLASTn against the ‘WGS contigs of *Aedes albopictus* (Skuse, 1894) (taxid:7160)’. Two Foshan, five FPA and two C6/36 contigs were identified to contain the PHB2 gene. The Rimini PHB2 sequence was assembled from a number of contigs whereas the Rimini_2 sequence is incomplete. Alignment of these contig sequences grouped them into the Foshan KQ571446.1 and the KQ562192.1 clusters which share 95–96 % nucleotide identity (SuppFig.S2a-b). The two alleles are present in the three mosquito isolates from Foshan, FPA and Rimini. Both of the two PHB2 gene sequences from the C6/36 WGS sequence are grouped with the KQ562192.1 cluster. Despite single nucleotide polymorphism (SNP), the aa sequences are almost identical apart from the 261I/V and 315R/K variants (SuppFig.S2c). PHB2 from the C6/36 WGS sequence has the 261 V and 315 K variants.

### Confirmation of C6/36 PHB2 exon 1 sequence

**Fig. 1:**
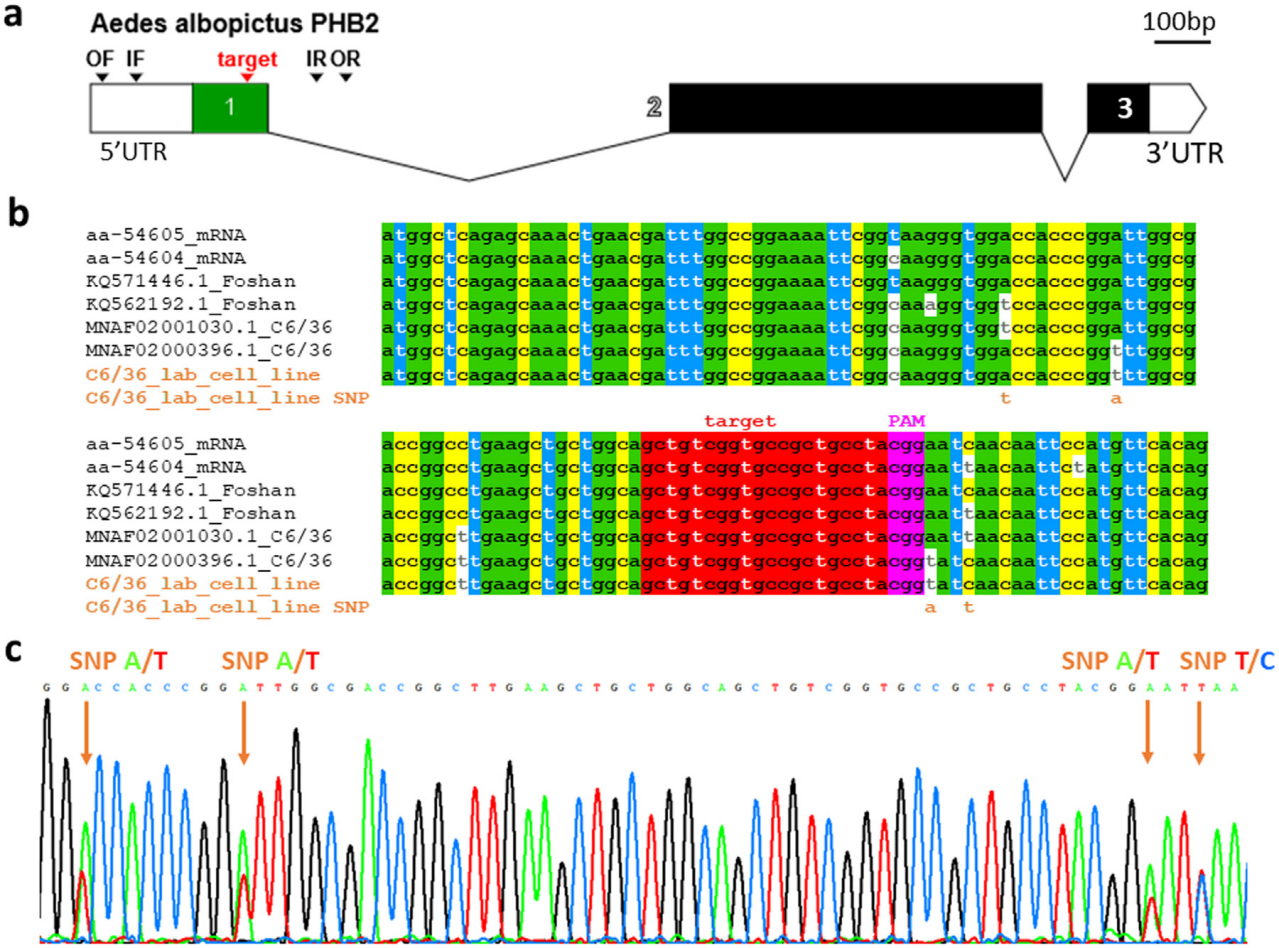
C6/36 prohibitin 2 exon 1 sequence. (a) The exon-intron organization of the *Aedes albopictus* prohibitin 2 (PHB2) gene is created using Exon-Intron Graphic Maker (http://wormweb.org/exonintron) which indicates the locations of exons 1–3 and the 5' and 3' untranslated regions (UTRs) and the locations of the target sequence and the primers (OF, IF, IR, OR) (Table 1) used to amplify up the exon 1 sequence from our laboratory-passaged C6/36 cell line for sequencing. **(b)** Alignment of the PHB2 exon 1 sequences from our laboratory-passaged C6/36 cell line (in brown label) with that of *Ae. albopictus* obtained from the Foshan (KQ571446.1, KQ562192.1) and C6/36 (MNAF02000396.1, MNAF02001030.1) whole genome shotgun sequences and the transcriptome shotgun assembly (Aa-54605, Aa-54604). Conserved sequences are colour-coded. It also highlights the target sequence subsequently chosen as the trans-activating CRISPR RNA (tracrRNA) in red and the protospacer adjacent motif (PAM) in violet. Single nucleotide polymorphism (SNP) is highlighted in brown alphabets. (c) A chromatogram illustrating SNP (brown arrow) in our laboratory-passaged C6/36 cell line PHB2 exon 1 sequence.

The *Ae. albopictus* PHB2 gene has three exons (Fig. 1a). Exon 1 was chosen as the target for CRISPR KO because of the higher probability of disrupting protein function when mutations occurred near the 5' end. Before proceeding to knock out the PHB2 gene from the cell line, C6/36, it was necessary to confirm the presence and sequence of the putative PHB2 gene in laboratory-passaged C6/36 cell line using nested PCR and outer and inner primer sets (Table 1, Fig. 1a). To sequence exon 1 of the PHB2 gene, primers were designed in the conserved 5' untranslated region (UTR) and intronic region that are flanking exon 1 using an alignment of the Foshan (KQ571446.1, KQ562192.1) and C6/36 WGS sequences (MNAF02000396.1, MNAF02001030.1) and the TSA (Aa-54605, Aa-54604) (SuppFig.S3). The primers were derived from the conserved sequences apart from a mismatch in the middle of the outer reverse (OR) primer within the MNAF02001030.1 sequence. Amplification of the PHB2 gene from the C6/36 genomic DNA by nested PCR followed by sequencing of the outer and inner PCR fragments in both orientations revealed SNP in the exon 1 sequence of the PHB2 gene from our laboratory-passaged C6/36 cell line and that each allele is identical to one of the two allelic sequences in MNAF02000396.1 and MNAF02001030.1 from the C6/36 WGS sequences (Fig. 1b-c).

**Table 1:** Primers used in this study.

primer	sequence
IF AeAlbop (IF)	5'-ATATTCCGGCGCGGTGATTG-3'
IR AeAlbop (IR)	5'-CCCCAATCAAGTTCCAGCGG-3'
OF AeAlbop (OF)	5'-TTGGTGTACGCGTTCCAAAG-3'
OR AeAlbop (OR)	5'-TCCCGGATTATGTCAATCCG-3'
AlbopPHB2sgRNAF	5'-TTCGCTGTCGGTGCCGCTGCCTA-3'
AlbopPHB2sgRNAR	5'-AACTAGGCAGCGGCACCGACAGC-3'
pAcSeqF	5'-GCCGAGTCAAATGCCGAATG-3'

*Procedures:* Genomic DNA was extracted from the C6/36 cells using PureLink® Genomic DNA Kits (Invitrogen) according to the manufacturer's instructions. Nested PCR was performed in a reaction containing 200 nM of each of the forward and reverse primers, 200µM dNTP and 0.025 U/µl *Taq* polymerase (New England Biolabs) using 2 min of 94 °C initial denaturation; 30 cycles of denaturation at 94 °C for 25 s, annealing at 57–59 °C for 35 s, and extension at 68 °C for 1 min with a final extension of 68 °C for 7 min. The outer fragment was amplified using the outer forward (OF) and outer reverse (OR) primers (Table 1) and 57 °C annealing temperature in 20µl PCR followed by amplification of the inner fragment using 1µl of the first PCR in 20 µl secondary PCR containing the inner forward (IF) and inner reverse (IR) primers (Table 1) and 59 °C annealing temperature. PCR product was cleaned by CleanSweep™ (Applied Biosystems) and sequenced using the Sanger sequencing services (Eurofins Genomics).

### Design of PHB2 tracrRNA to generate sgRNA

Successful CRISPR KO depends largely on the design of trans-activating CRISPR RNAs (tracrRNAs) with good on-target and low off-target activities. The exon 1 sequence of the C6/36 PHB2 gene exhibits SNP in four positions (Fig. 1b, c). By including the SNP, we manually identified 17 target sequences (including six SNP) in the sense orientation and 13 (including 12 SNP) in the antisense orientation with a C-terminal NGG protospacer adjacent motif (PAM) (Table 2). To illustrate the possibility of extending a target sequence into the intronic region, the genomic exon sequence was extended one base into the intronic region to generate the NGG PAM for the last two sense tracrRNAs.

**Table 2:** Target sequences in exon 1 of the C6/36 cell line prohibitin 2 gene and off-target predictions.

^a^The target sequences were read from the 5'−3' direction for both sense and anti-sense strands. NGG protospacer adjacent motifs (PAMs) are highlighted in pink. Overlapping PAMs are underlined. The extra G (in sense strand that forms part of the PAM) and C (in anti-sense strand) in the intronic region are in blue italics. Targets were manually identified and confirmed with Cas-Designer. The selected target sequence is in red. Target sequence with four ‘T’ can cause termination of RNA polymerase III and is greyed out. Lowercase letters indicate single nucleotide polymorphism (SNP) (Fig. 1). Sequences highlighted in beige are the eight entries returned by CRISPR GuideXpress.

^b^indicates the number of mismatches with the target sequence as predicted by Cas-OFFinder. Target sequences with two 0 mismatches, without 1 and 2 mismatches and ≤5 three mismatches are highlighted in yellow.

^c^indicates the probability of generating out-of-frame insertions and deletions in CRISPR-edited site as predicted by Cas-Designer. Since the out-of-frame scores are calculated in the context of neighbouring gene sequences, there are two out-of-frame scores (separated by a slash) for some identical sequences due to the presence of neighbouring SNP in the allelic genes. A score of >66 is recommended and those with a score >66 are highlighted in yellow.

^d^indicates %GC as predicted by Cas-Designer and the recommended range is 20–80 %.

Off-target effects are the Achilles’ heel of CRISPR-editing. Binding of single guide RNAs (sgRNAs) to similar but mismatched targets can produce off-target effects by knocking out the wrong targets. These off-target effects are unpredictable and confounding. To minimize off-target effects, the on-target tracrRNAs should be screened against the genome of the organism or the cell line to identify potential off-target hits. A number of company- and academic-supported design tools are available but many are limited to commonly studied organisms such as human, mouse, rat, zebra fish and *Caenorhabditis elegans*. Several tools support a wide range of species including mosquitoes and pathogens but not cell lines. These include Benchling (https://www.benchling.com/crispr) and e-CRISP (http://www.e-crisp.org). CHOPCHOP (http://chopchop.cbu.uib.no/) [25] and CRISPOR (http://crispor.tefor.net) [26] are the only two supporting *Ae. albopictus* mosquito but not cell lines. Eventually, we used the Cas-Designer [27] and Cas-OFFinder [28] inside the CRISPR RGEN Tools (http://www.rgenome.net) which have both the whole genome sequences of the Asian tiger mosquito, *Aedes albopictus*, and the C6/36 cell line added on our request. The Cas-Designer returned the same tracrRNAs manually predicted by us (Table 2). All of the tracrRNAs have within the recommended 20–80 % GC content. The Cas-Designer de-selected the target sequences with four ‘T’ that can cause termination by RNA polymerase III. The Cas-Designer also predicted the number of mismatches (off-targets) throughout the genome and calculated the possibility of creating out-of-frame INDELs. Since the Cas-Designer only predicted up to two mismatches, we also used Cas-OFFinder to predict three mismatches which was the cut-off we set for our tracrRNA design. In this study, we prioritized on-target match and off-target effects over out-of-frame score. Twenty target sequences (including the two with four ‘T’) with SNP (i.e. one 0 mismatch) were not selected. Nine sense and one antisense tracrRNA sequences returned two 0 mismatches i.e. they completely match their two target sequences in contigs MNAF02000396.1 and MNAF02001030.1; confirming the absence of SNP (see SuppTable S1 for an example). Out of those with two 0 mismatches (on-targets) and without 1 or 2 mismatches (off-targets), six of the sense tracrRNA sequences displayed ≤5 three mismatches (off-targets). Due to the possibility of using alternative ATG start codon after disrupting 5' end sequence, the 3' end target sequences of exon 1 were preferred. The 3' end tracrRNA sequence GCTGTCGGTGCCGCTGCCTA has five off-targets (SuppTable S1a). Four out of the five have PAM-proximal mismatches; further reducing the possibility of binding to off-targets. Another 3' end tracrRNA sequence GGCGACCGGCTTGAAGCTGC has four off-targets but only two have PAM-proximal mismatches (SuppTable S1b). The tracrRNA GCTGTCGGTGCCGCTGCCTA has an out-of-frame score of 56.7/58.3 which is close to the recommended 66; hence, GCTGTCGGTGCCGCTGCCTA was chosen as the tracrRNA sequence to generate sgRNA. Overall, Cas-OFFinder offers more control and flexibility over the input sequence and more interrogation of the output results.

A new bioinformatics portal called CRISPR GuideXpress (https://www.flyrnai.org/tools/fly2mosquito/web/) has since been developed specifically for single and batch sgRNA design in mosquitoes and mosquito cell lines [9] but is limited to genomic sequences and annotation files available from the VectorBase (http://vectorbase.org). We attempted this bioinformatics tool for the C6/36 PHB2 gene without success even after retrieving the gene IDs from the VectorBase and located its chromosomal locations. Input of our 30 target sequences as identified manually by us and Cas-Designer into CRISPR GuideXpress returned 16 tracrRNAs derived from the SNP sequences (Table 2 beige highlight). The CRISPR GuideXpress worked slightly better with the mosquito, *Ae. albopictus*. The orthologue search in the CRISPR GuideXpress identified two transcript variants of the *Ae. albopictus* PHB2 gene from the VectorBase, which are identical to the KQ562192.1 and KQ571446.1 variants that we obtained from the NCBI database above. Using the two transcript IDs as search criteria, only seven of the 57–62 tracrRNAs are derived from exon 1 and they are all derived from the SNP sequences (SuppTable S2).

Information on the off-target sequences and chromosomal locations (SuppTable S1a) can be used to design PCR primers to check for off-target effects in KO cell lines without or preceding the use of costly next-generation sequencing. This could be done by using Primer-BLAST and the accession number MNAF02000xxxx of the off-target in SuppTable S1a as an input PCR template and 500 bases 5' and 3' to the off-target position as the primer range. An example of the Primer-BLAST result is shown in SuppTable S1c.

### Cloning of PHB2 tracrRNA oligonucleotides to generate sgRNA plasmid

**Fig. 2:**
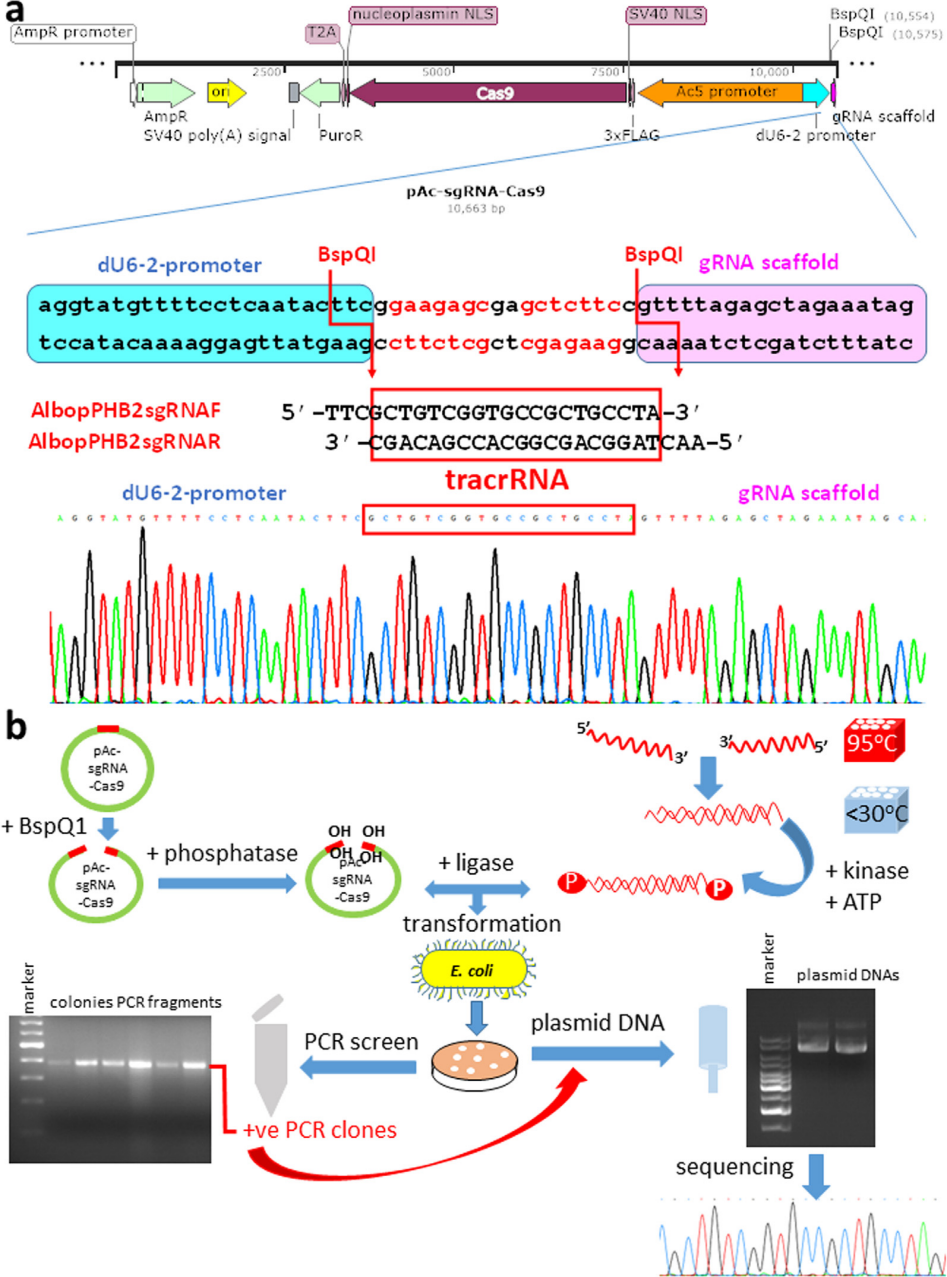
Cloning of the CRISPR RNA oligonucleotides to create single guide RNA. **(a) (top)** Plasmid map of pAc-sgRNA-Cas9 showing the BspQI cloning sites and the guide RNA (gRNA)-scaffold under the dU6–2 promoter and the Cas9 under the Ac5 promoter. The plasmid contains ampicillin- (AmpR) and puromycin-resistant (PuroR) genes. The plasmid map was created by SnapGene Viewer. **(middle)** Part of the plasmid map is enlarged to show the sequences of the dU6–2 promoter, the BspQ1 restriction enzyme sites and the gRNA scaffold. The trans-activating CRISPR RNA (tracrRNA) oligonucleotides AlbopPHB2sgRNAF and AlbopPHB2sgRNAR (Table 1) to be inserted into the vector are displayed underneath and boxed in red. **(bottom)** Chromatogram showing the correct insertion of the tracrRNA sequence (boxed in red) into the vector to create single guide RNA (sgRNA). **(b)** Schematic of the cloning procedures. **(left)** the plasmid vector was digested with BspQ1 and then dephosphorylated. **(right)** The forward and reverse oligonucleotides were annealed by heating at 95 °C and then cooling to <30 °C. The annealed oligonucleotides were phosphorylated and ligated into the dephosphorylated vector. The ligation mix was transformed into competent *Escherichia coli* cells and colonies were screened by PCR. Plasmid DNA was extracted from positive clones and sequenced.

The insect plasmid vector pAc-sgRNA-Cas9 (Addgene #49330) contains the BspQ1 cloning sites for tracrRNA downstream of the dU6–2 promoter and Cas9 downstream of the actin-5c promoter (Fig. 2a). Forward (AlbopPHB2sgRNAF) and reverse (AlbopPHB2sgRNAR) oligonucleotides (Table 1) were designed for cloning the PHB2 tracrRNA sequence into the vector for sgRNA production, according to the protocol of [29] with modifications (Fig. 2b). TTC and AAC were added to the 5' end of the forward and reverse oligonucleotides, respectively, to create BspQ1 sites (Fig. 2a). Since the tracrRNA sequence contains a 5' terminal G, it was not necessary to add an additional G to the 5' end to allow transcription from the dU6–2 promoter.

*Procedures:* Twenty-five micromolars of each of the forward (AlbopPHB2sgRNAF) and reverse oligonucleotides (AlbopPHB2sgRNAR) (Table 1) were annealed in 20µl of annealing buffer (10 mM Tris–HCl, 1 mM EDTA, 50 mM NaCl, pH8) by heating for 5 min in a 95 °C heat block which was then removed from the heating unit to allow it to slowly cool to <30 °C (Fig. 2b). The annealed reaction was then put on ice. One microlitre of the annealed oligonucleotides was phosphorylated using 1 U/µl of T4 polynucleotide kinase (PNK) (New England Biolabs) and 1 mM ATP in a final volume of 10µl for 30 min at 37 °C.

Two micrograms of the plasmid vector pAc-sgRNA-Cas9 (Addgene #49330) (Fig. 2a) were digested with 0.4 U/µl of BspQ1 (New England Biolabs) in a final volume of 50µl and dephosphorylated by 1 unit of Anza™ Alkaline Phosphatase (Invitrogen) at 37 °C for 10 min followed by heat inactivation of the phosphatase at 80 °C for 5 min.

Two microliters of the 10x diluted annealed oligonucleotides were ligated to 1.7 µl of the BspQ1-digested vector in Anza™ T4 DNA Ligase Master Mix (Invitrogen) in a final volume of 10 µl at RT for 1 h (Fig. 2b). Two microliters of the ligation mix were transformed into 100µl competent *Escherichia coli* TG1 cells and plated on ampicillin agar plates. Colonies were picked into individual PCR mix and screened for the presence of insert using 0.025 U/µl *Taq* polymerase (New England Biolabs), 200 µM dNTP and 200 nM of each of the primer pair, pAcSeqF and AlbopPHB2sgRNAR (Table 1) using 2 min of 94 °C initial denaturation; 30 cycles of denaturation at 94 °C for 25 s, annealing at 59 °C for 35 s, and extension at 68 °C for 1 min with a final extension of 68 °C for 7 min. The PCR fragments were resolved by 1 % TAE (0.4 M Tris-acetate, 0.01 M EDTA, pH8.3) agarose gel electrophoresis for 1 h at 100 V to confirm the presence of insert. DNA was extracted from positive colonies using QIAprep Spin Miniprep Kit (Qiagen) according to the manufacturer's instructions and sequenced using pAcSeqF primer (Table 1) by the Sanger sequencing services (Eurofins Genomics).

### Selection of KO cells

The plasmid would then be used to transfect mosquito C6/36 cells for the selection of KO clones. Insect cells were refractory to transfection; hence, an efficient method for transfection of the C6/36 cells was required. A number of transfection reagents were tested using a plasmid encoding β-galactosidase and X-gal staining of transfected cells. These included calcium phosphate [30,31], FuGENE® HD/XtremeGene HP (Roche) [32,33], Trans-IT 2020/Trans-IT X2 (Mirus), PolyFect/SuperFect/Effectene (Qiagen) [[34], [35], [36]], DreamFect™ (Oz BioSciences) and JetPRIME® (PolyPlus). Lipofectamine 3000 (Invitrogen) and XtremeGene 9 (Roche) gave the best transfection efficiencies of ≥10 % which might be an underestimation due to the use of CMV-driven β-galactosidase. Lipofectamine 3000 had a slightly higher transfection efficiency and a low level of cell clumping whereas XtremeGene 9 was non-toxic; therefore, both were selected to be the transfection reagents.

#### Transfection procedures

C6/36 cells were seeded at 9 × 10^6^ cells per 100 mm dish and transfected according to the manufacturer's instructions with modifications. For transfection with XtremeGene 9 (Roche), 5µg of the plasmid DNA were added to 15µl of XtremeGene 9 (Roche) pre-diluted in 500µl Opti-MEM™ (Gibco). For transfection with Lipofectamine 3000 (Invitrogen), 15µg of the plasmid DNA were diluted in 750µl Opti-MEM™ (Gibco) and 30µl of the P3000™ Reagent (Invitrogen). The mixture was added to 45µl of Lipofectamine™ 3000 (Invitrogen) pre-diluted in 750µl Opti-MEM™ (Gibco). The cells were transfected for a total of 48 h.

#### Kill curve procedures

The plasmid pAc-sgRNA-Cas9 contains a puromycin-resistant gene for cell selection (Fig. 2a). A kill curve was used to determine the concentration of puromycin (2µg/ml) for selection in the C6/36 cell line. C6/36 cells seeded at 1.5 × 10^5^ cells/well of a 24-well plate were treated with serial dilutions of puromycin (Sigma-Aldrich). The drug was replenished twice per week for 2 weeks and then removed for another week. The concentration of puromycin in the wells without resistant clones was selected.

#### Selection of edited clones procedures

**Fig. 3:**
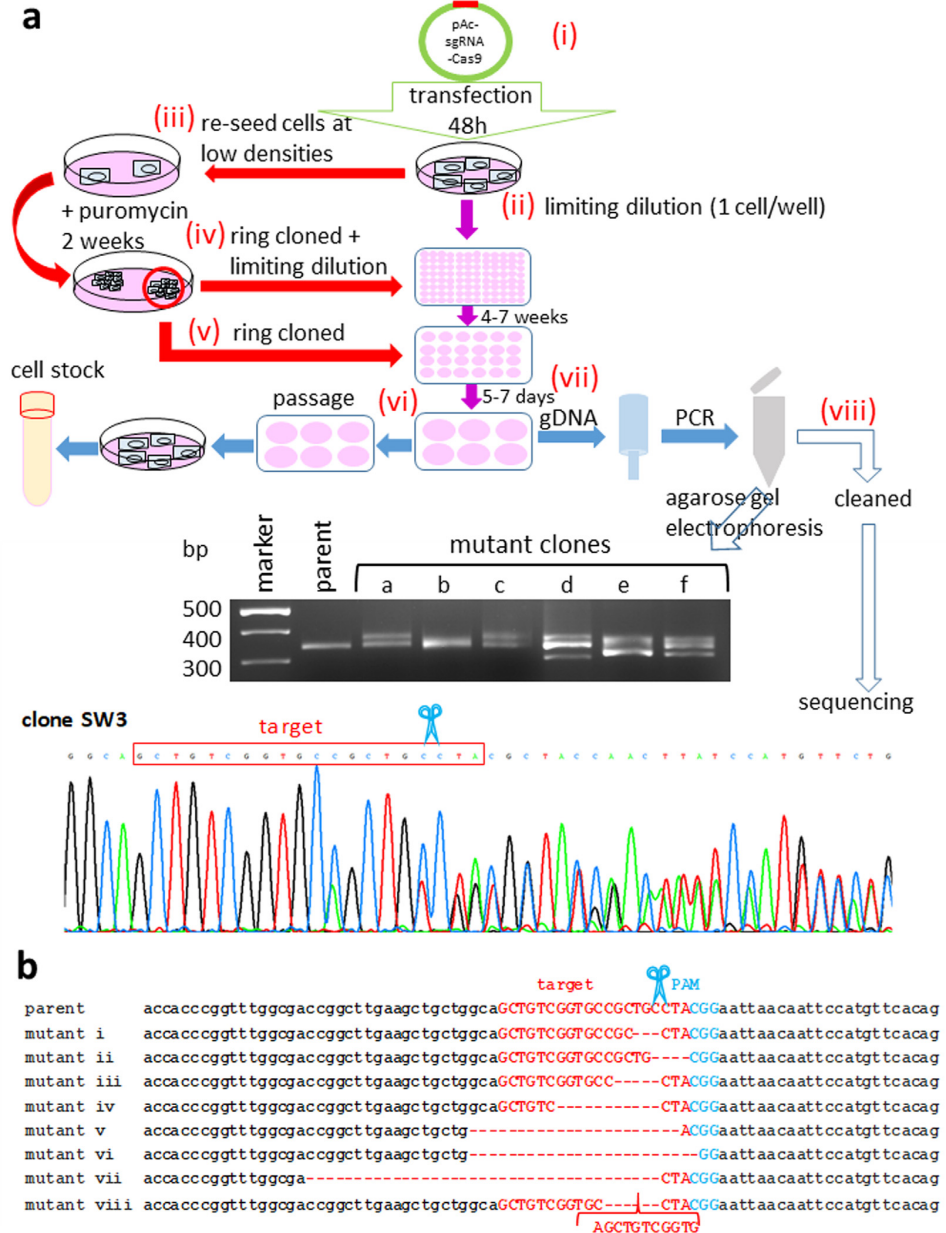
Selection of CRISPR-edited cells. **(a)** Schematic of the selection procedures. **(i)** Plasmid containing the single guide RNA (sgRNA) or the empty vector control was transfected into C6/36 cells for 48 h. **(ii)** Transfected cells were seeded at 1 cell per well in a 96-well plate by limiting dilution and then expanded into 24- and then 6-well plates. **(iii)** Transfected cells were re-seeded at low densities in 100 mm dishes and then selected by 2µg/ml puromycin for 2 weeks. Puromycin was then removed and cells were cultured until colonies appeared. Cell colonies were either **(iv)** ring-cloned and then seeded at 1 cell per well in a 96-well plate by limiting dilution and then expanded into 24- and then 6-well plates; or **(v)** ring-cloned and transferred directly into 24-well plate and then expanded into 6-well plate. When cells became confluent in 6-well plate, **(vi)** half was maintained in cell passage until confirmation of insertions and deletions (INDELs) before expanding to make cell stocks; **(vii)** half was harvested for genomic DNA (gDNA) extraction for PCR using primer pair IF and IR (Table 1). The PCR products were resolved by agarose gel electrophoresis to identify the INDELs. An example of an agarose gel showing INDELs is shown. **(viii)** Mutant clones with INDELs were further sequenced. An example of a chromatogram with mixed sequences is shown. The target sequence is boxed in red and the DNA site cleaved by Cas9 is denoted by a blue scissor. **(b)** Sequence alignment of the parental and edited PHB2 genes showing a repertoire of INDELs predicted by decomposition of mixed sequences using CRISPR-ID. The target sequence is in red and the protospacer adjacent motif (PAM) in blue. The DNA site cleaved by Cas9 is denoted by a blue scissor.

To perform CRISPR KO, the plasmid pAc-C6/36.PHB2-sgRNA-Cas9 was transfected into C6/36 cells, in parallel with the empty vector control plasmid, pAc-sgRNA-Cas9 (Fig. 3a). After 48 h of transfection selection of edited clones was performed in two ways (Fig. 3a). In the first method, transfected cells were seeded at one cell/well of a 96-well plate by limiting dilution. When cells in 96-well plate were grown to confluence, cells were transferred to 24-well and then 6-well plate. In the second method, transfected cells were re-seeded at low densities in 100 mm dish. After an overnight incubation, 2µg/ml puromycin were added and replenished twice per week for two weeks and then removed until colonies were visible. Individual colonies were either purified by seeding at one cell/well of a 96-well plate by limiting dilution as above or directly transferred into 24-well plate and then 6-well plate. When cells became confluent in 6-well plate, the medium was replaced with 2 ml of fresh medium before dislodging cells. Half of the dislodged cells was extracted for genomic DNA using PureLink® Genomic DNA Kits (Invitrogen) according to the manufacturer's instructions. Another 1 ml of fresh medium was added to the well to keep cell passaging until insertions/deletions (INDELs) identified and cell stocks made.

One microgram of extracted genomic DNA from each cell clone was used in *Taq* polymerase PCR to amplify the PHB2 exon 1 fragment using primer pair, IF and IR (Table 1) and 54 °C annealing temperature (Fig. 3a). PCR fragments were resolved by 2–3 % TAE agarose gel electrophoresis for 1–2 h at 100 V. INDELs were identified by comparing the sizes of the fragments with parental PCR fragments. PCR product was cleaned by CleanSweep™ (Applied Biosystems) and sequenced using the Sanger sequencing services (Eurofins Genomics).

### Identification of INDELs by decomposition

Since CRISPR acts on the two gene alleles independently to create random mutations resulting in different INDELs in the two gene alleles, direct sequencing from genomic DNA will generate mixed sequences after the cut site (Fig. 3a chromatogram). A number of computer programs are available to decompose mixed sequences. Tracking of INDELs by DEcomposition (TIDE) (http://tide.nki.nl) [37] can be used to predict the number and percentage of INDELs but not the sequences. CRISPR-ID (http://crispid.gbiomed.kuleuven.be) [38], Inference of CRIPSR Edits (ICE) (http://ice.synthego.com) [39], DEconvolution of Complex DNA Repair (DECODR) (https://decodr.org) [40] and Tracy Indigo application (http://www.gear-genomics.com) [41] etc. can decompose mixed sequences. ICE, DECODR and Tracy are more user-friendly. CRISPR-ID allows users to change the decomposition criteria.

Mono- and bi-allelic KO cell clones are the end-products and can be used for functional study of the PHB2 upon confirmation of INDELs in the allelic sequences and expression levels of the PHB2 protein (an example is illustrated below). Mono-allelic KO clones can also be re-CRISPRed to target the remaining wt allele, if required. Mixed clones can be further purified using limiting dilution and then re-characterized as above.

### Identification of INDELs by cloning into pBluescript for sequencing

For cloning into pBluescript, the PHB2 exon 1 fragment was amplified from 1µg of gDNA extracted from the edited cell clone using 0.02 U/µl high fidelity Q5 DNA polymerase (New England Biolabs), 500 nM of each of the forward and reverse primers, IF and IR (Table 1) and 200µM dNTP. The PCR conditions were 30 s of 98 °C initial denaturation; 30 cycles of denaturation at 98 °C for 10 s, annealing at 69 °C for 30 s, and extension at 72 °C for 30 s with a final extension at 72 °C for 2 min. The PCR products were purified using the NucleoSpin Gel and PCR Clean Up Kit (Machery Nagel) according to the manufacturer's instructions. Since the PCR products from Q5 polymerase amplification were blunt-ended, the pBluescript plasmid was digested with 0.5 U/µl *EcoR*V (Promega) to create blunt ends which was then purified by the NucleoSpin Gel and PCR Clean Up Kit (Machery Nagel). Then, 2.5µg of the vector were dephosphorylated by incubating with 3 units of Anza™ Alkaline Phosphatase (Invitrogen) in a total volume of 60µl at 37 °C for 15 min followed by inactivation at 80 °C for 5 min and purification using the NucleoSpin Gel and PCR Clean Up Kit (Machery Nagel). The insert (PCR fragment) was ligated to the vector in a 9:1 molar ratio using Anza™ T4 DNA Ligase Master Mix (Invitrogen) at RT for 30 min. The ligation mix was transformed into competent *E. coli* cells and plated on ampicillin plate containing 50µl of 40 mg/ml X-Gal and 50µl of 80 mM isopropyl-β-d-thio-galactopyranoside for blue-white screening. White colonies were picked into individual PCR mix and screened for the presence of insert using *Taq* polymerase PCR and the primer pair, IF and IR (Table 1) and 54 °C annealing temperature. White colonies with inserts were selected for plasmid DNA preparation using the QIAprep Spin Miniprep Kit (Qiagen) according to the manufacturer's instructions. The cloned insert was sequenced in both orientations using primers IF or IR (Table 1) by the Sanger sequencing services (Eurofins Genomics).

## Method validation

### Creation of sgRNA plasmid vector

Cloning of the PHB2 sgRNA into the plasmid vector generated 100 % positivity in 17 colonies screened. Six colonies were selected for further plasmid DNA preparation and sequenced using pAcSeqF primer (Table 1) to confirm the sequence of the insert (Fig. 2a chromatogram). The plasmid is named pAc-C6/36.PHB2-sgRNA-Cas9.

### Identification of INDELs

The plasmid pAc-C6/36.PHB2-sgRNA-Cas9 was transfected into C6/36 cells and cell clones were characterized for INDELs by agarose gel electrophoresis of PCR fragments amplified up from genomic DNAs using primers IF and IR (Table 1). An example is shown in Fig. 3a. The two alleles from the parental cells are of the same size and hence migrated as one band. Cell clone b has wild type (wt) genes. Mutant cell clones a and c contain mono-allelic insertions, hence one band migrated the same as the wt band and the other band migrated slower than the wt band. Mutant cell clone e is a bi-allelic KO clone; containing insertion in one allele and deletion in the other allele. Mutant clones d and f are mixed; containing wt band, insertion and deletion.

**Fig. 4:**
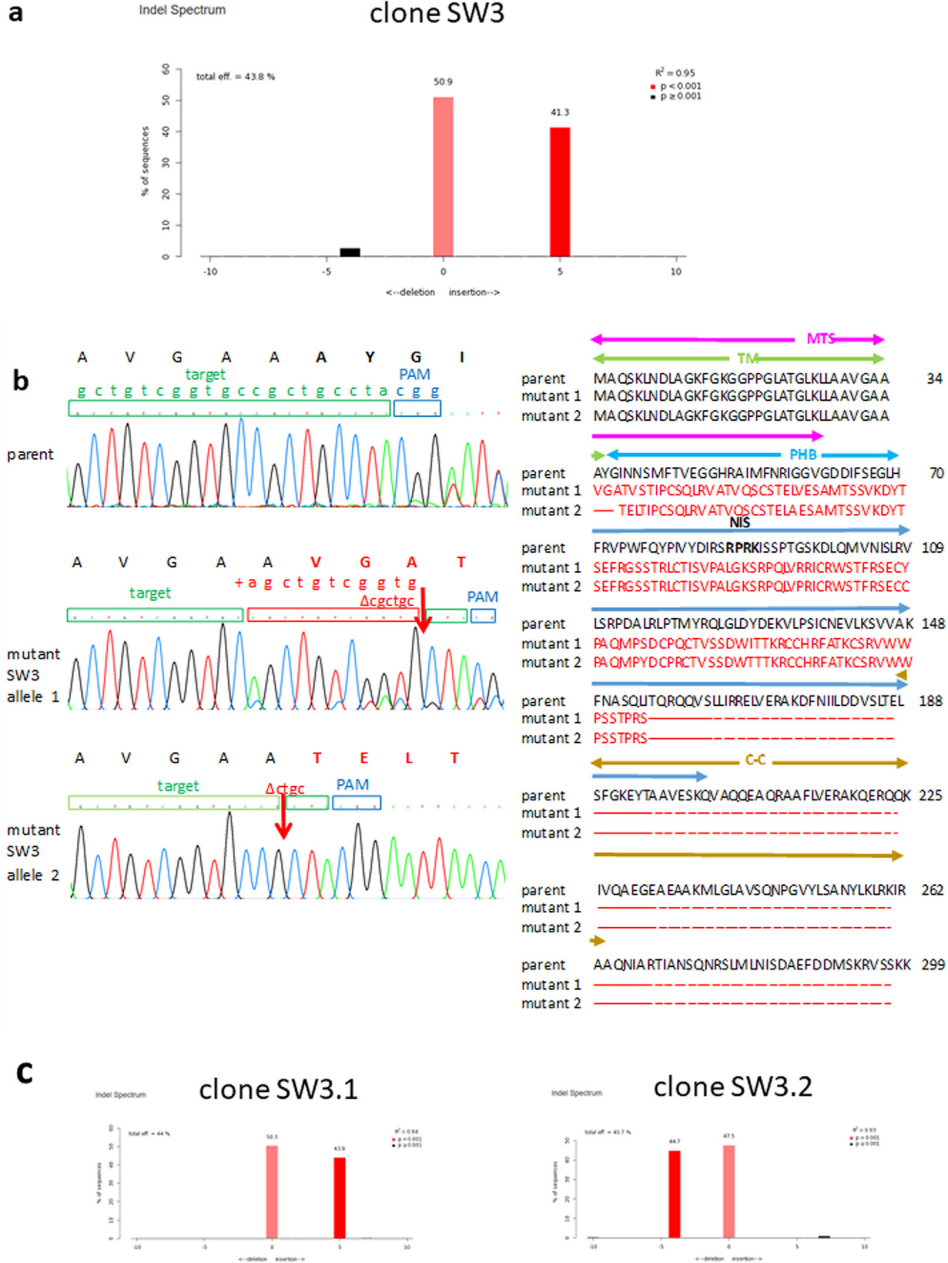
CRISPR gene editing changes PHB2 DNA sequences giving rise to frameshifted and truncated, non-functional proteins. **(a)** TIDE prediction of insertions and deletions (INDELs) in the mutant clone, SW3. **(b) (left panel)** Chromatograms showing the target sequence and protospacer adjacent motif (PAM) in the parental sequence of the prohibitin 2 (PHB2) gene and the INDELs in the two cloned allelic gene fragments from the CRISPR-edited mutant cell clone, SW3. Red arrow indicates the position where the DNA was cleaved by Cas9 and INDELs (in red box and alphabets) occurred. Frameshift is highlighted in red single-letter amino acid code above the chromatogram. **(Right panel)** Alignment of translated amino acid sequences of the PHB2 gene from the parental cells and the two cloned allelic gene fragments from the CRISPR-edited mutant cell clone, SW3. Red indicates frameshift and truncation. The PHB2 domains are delineated above the amino acid sequence and are colour-coded: transmembrane (TM) in green, PHB in blue, coiled-coil (CC) in brown and unconventional mitochondrial targeting sequence (MTS) in pink. The nuclear import signal (NIS) within the PHB domain is in bold. **(c)** TIDE prediction of INDELs in the two sub-clones, SW3.1 and SW3.2, isolated by re-cloning of the mutant clone, SW3.

To identify the INDELs, mutant clones were directly sequenced from genomic DNAs. Since CRISPR acts on the two gene alleles independently to create random mutations resulting in different INDELs in the two gene alleles, direct sequencing from genomic DNA will generate mixed sequences after the cut site (Fig. 3a chromatogram). To decompose the allelic sequences we used a number of computer programs. CRISPR-ID was the only program able to predict the correct deletion of cgctgc and insertion of agctgtCGGTG in mutant sequence viii (Fig. 3b) (which was later confirmed by molecular cloning (see below Fig. 4b mutant SW3 allele 1)). Both DECODR and Tracy correctly predicted the insertion of CGGTG only but not the replacement of cgctgc by agctgt. ICE predicted the wrong insertion of CTACG and also failed to predict the replacement. A repertoire of predicted INDELs is depicted in Fig. 3b, illustrating the diversity of INDELs generated by CRISPR KO. Sequence decomposition correctly predicted wt, mono-allelic, bi-allelic and mixed KO cell clones to correspond with agarose gel migration.

To obtain precise sequences of the INDELs, the gene alleles were separated by molecular cloning into pBluescript for plasmid sequencing. We illustrate this using cell clone SW3. SW3 was selected by limiting dilution in the absence of puromycin. Sequencing of the genomic DNA generated mixed sequences of half-peak size after the Cas9 cut site (i.e. three nucleotides before the PAM) (Fig. 3a chromatogram), in consistent with the presence of two alleles in diploid C6/36 cells (each of the half-peak represents one allele). TIDE predicted a mono-allelic KO with five insertions (50.9 % wild type allele; 41.3 % insertion allele; *p* < 0.001) together with a very small percentage (2.6 %; *p* = 0.0035) of a minor allele with four deletions (Fig. 4a). ICE was less powerful and could only predict the four deletions (but incorrect sequences) when the percentage of the minor allele increased to 4.2 % (data not shown). CRISPR-ID, DECODR and Tracy did not predict a deletion (data not shown). To confirm the INDELs, the gene alleles were amplified up using high fidelity Q5 polymerase (New England Biolabs) and cloned into pBluescript for sequencing. One of the cloned sequences revealed that the five insertions is a result of a deletion of the parental ‘CGCTGC’ and an insertion of ‘AGCTGTCGGTG’ (Fig. 4b mutant SW3 allele 1), which was correctly decomposed by CRISPR-ID (Fig. 3b mutant viii) but not the other programs. Another cloned sequence confirmed the presence of the minor allele with ‘CTGC’ deletion correctly predicted by TIDE (Fig. 4b). It proved that our methods were able to clone a gene fragment which is present in such a small percentage (2.6 %) that had escaped detection by CRISPR-ID, DECODR and Tracy. The results suggest that SW3 is a mixed cell clone dominated by a mono-allelic 5-insertion mutant. Upon re-cloning, the mutant cell clone, SW3, was separated into two cell clones, SW3.1 (with a mono-allelic 5-insertion) and SW3.2 (with a mono-allelic 4-deletion) (Fig. 4c). Therefore, our results show that the mutant cell clone, SW3, was made up of a major mono-allelic 5-insertion cell clone and a minor mono-allelic 4-deletion cell clone. Both of these mutations resulted in frameshift after alanine (A) at position 34 in exon 1 and premature termination; giving rise to a truncated protein (Fig. 4b). The PHB2 is divided into the transmembrane (TM), PHB and coiled-coil (CC) domains [42] and an unconventional mitochondrial targeting sequence (MTS) spanning aa1–61 [43]. The PHB domain plays a multi-functional role and contains a putative nuclear import sequence [42]. Part of the PHB domain is also involved in protein-protein interactions [44,45]. The CC domain mediates PHB2 interactions with PHB1 and other proteins [42]. The mutations in SW3 have removed half of the MTS (aa35–61) and all of the PHB and CC domains; therefore, the mutated proteins are likely non-functional and degraded, similar to the *Saccharomyces cerevisiae* PHB2 ∆aa36–61 which did not accumulate in the mitochondria [43].

### CRISPR-editing knockdowns PHB2 expression

**Fig. 5:**
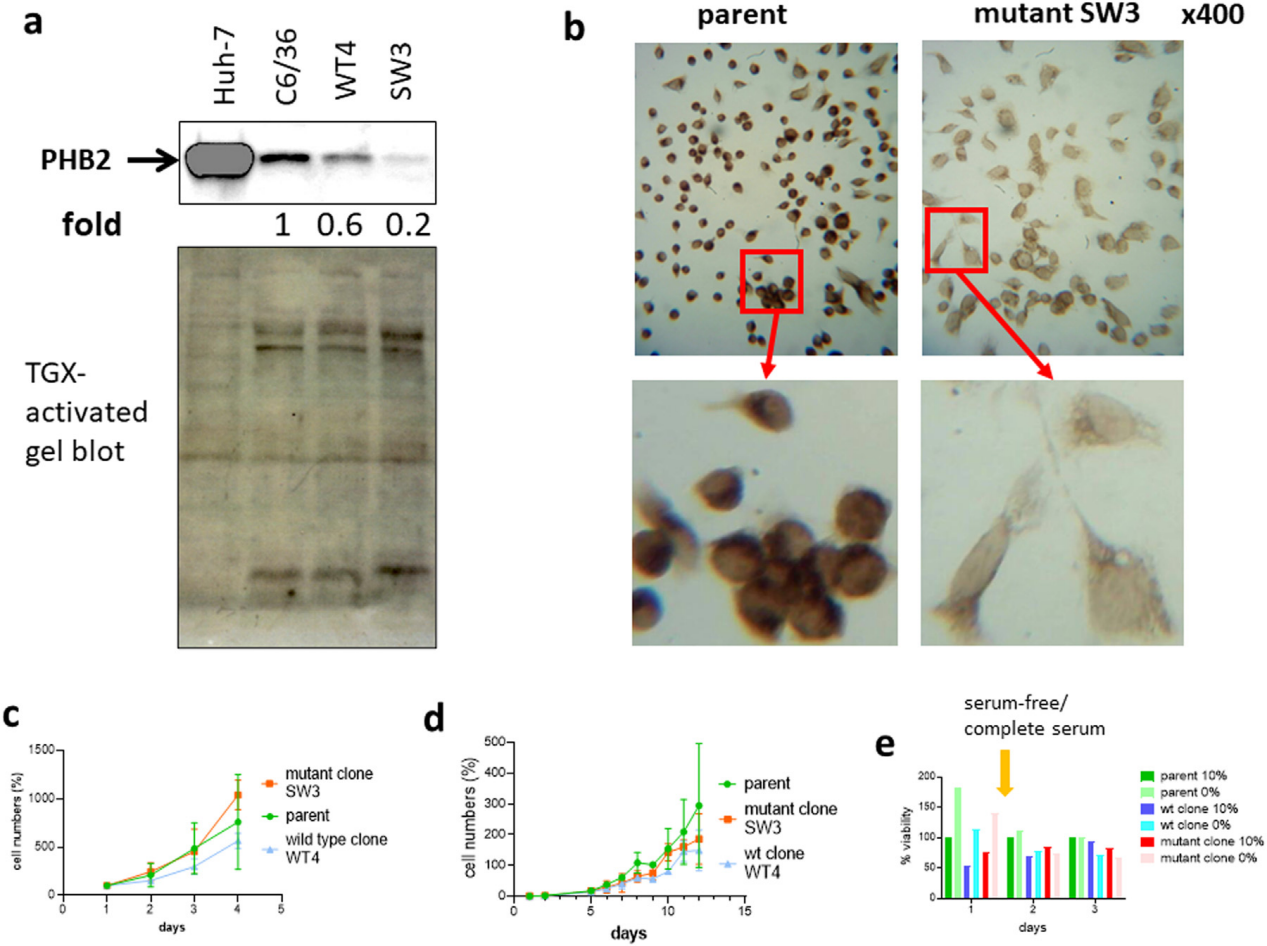
CRISPR-edited mutant cell clone has reduced prohibitin 2 expression. **(a)** Western blotting of parental C6/36 cells, a CRISPR-edited mutant clone (SW3) and a vector control-edited cell clone retaining the wild type (wt) genotype (wt clone WT4). Equal amounts of proteins were separated on TGX-stain-free SDS-PAGE gel and probed against the anti-prohibitin 2 (PHB2) antibody. The human hepatocyte cell line, Huh-7 was used as a positive control and was loaded at 1/6th of the quantity. Arrow indicates the PHB2 protein band. Total protein was used as a loading control. Fold change in protein quantity is indicated below the protein band. The anti-PHB2 antibody (Novus) was raised against the C-terminal half of the human PHB2 (SuppFig.S1), and, therefore, will not recognize the mutated PHB2. **(b)** Immunocytochemistry of parental C6/36 cells and a CRISPR-edited mutant clone, SW3, using anti-PHB2 antibody. The selected areas were enlarged to show details of the staining. The anti-PHB2 antibody (Proteintech) was raised against the human full-length protein. Images are of the same magnification and on the same scale. **(c)** Growth curves of parental C6/36 cells, SW3 and WT4 during early growth phase. Cells were seeded at 1 × 10^5^ in a 6-well plate and counted over a course of four days. Data are expressed as % of cell numbers on day 1. Results are presented as mean+/-SD of two repeats. **(d)** Growth curves of parental C6/36 cells, SW3 and WT4 over an extended period. Cells were seeded at 1–6 × 10^4^ in a 12-well plate and counted over a course of 12 days. Data are expressed as % of seeding density. Results are presented as mean+/-SD of two repeats. Data were analyzed by two-way ANOVA with Tukey's post-hoc test. A p value of <0.05 was considered statistically significant. **(e)** Comparison of cell growth of parental C6/36 cells, SW3 and WT4 in serum-free medium (0 %) and complete medium (10 %). Cells were seeded at 60,000 cells in a 96-well plate on Day 0. Cell viability was measured on Day 1 before cell medium was changed to serum-free or complete media to obtain the starting cell densities. Cell viability under serum-free or complete media conditions was measured on Days 2 and 3. Data are expressed as % of parental cells in complete medium (10 %) on the same day. (*n* = 1).

We further proved that mono-allelic KO was sufficient to greatly reduce PHB2 expression of >80 % in the cell population and in single cells, as detected by Western blotting (Fig. 5a) and immunocytochemistry (Fig. 5b), respectively. The reduction in PHB2 expression was much greater than the clonal variations in PHB2 expression in wt cells (between parent and wt clone WT4). This confirms that our methods were able to knockdown gene expression at the protein level. Homozygous deletion of *phb2* is lethal in mice [46]. Our results suggest that heterozygous deletion may be sufficient to suppress PHB2 expression to a level low enough for functional study.

### KO cells undergoes phenotypic switching

**Fig. 6:**
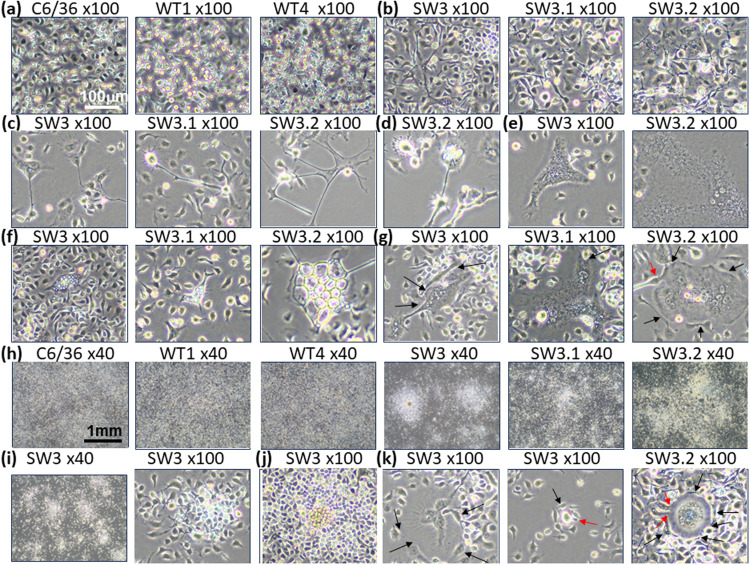
CRISPR-edited mutant cell clones undergo phenotypic switching. Microscopic images showing the morphology of parental C6/36 cells, CRISPR-edited mutant clone, SW3 and sub-clones, SW3.1 and SW3.2 and vector control-edited cell clones retaining the wild type (wt) genotype (wt clones WT1 and WT4). Phase contrast images were acquired using an Olympus CKX53 and Olympus EP50 camera and OSD software. Images of the same magnification are on the same scale. red arrow=putative cell fusion; black arrow=cell-cell contact.

**Fig. 7:**
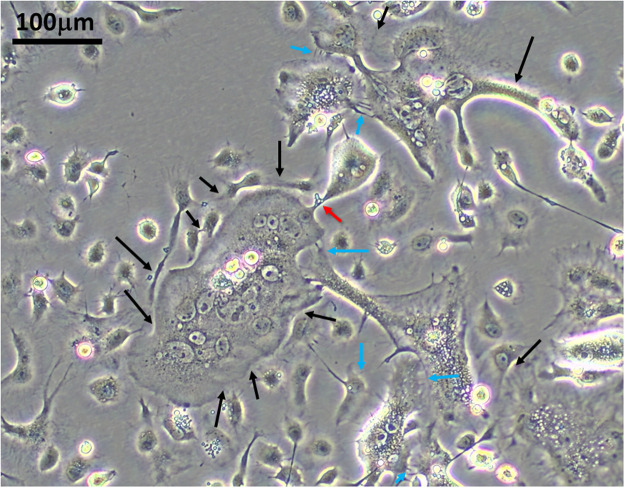
Putative cell fusion in PHB2 mutant cells. Microscopic images showing the formation of giant multinucleated cells from putative cell fusion in SW3.2. Phase contrast images were acquired using an Olympus CKX53 and Olympus EP50 camera and OSD software. red arrow=putative cell fusion or cell pinching; blue arrow=cell-cell contact by finger-like cytoplasmic protrusion; black arrow=sideway cell-cell contact.

We further characterized the effects of PHB2 KO on cell morphology and growth rates. The KO cells were morphologically distinct from the parental (wt) cells and wt clones WT1 and WT4 (Fig. 6). WT1 and WT4 were two non-target control sgRNA-selected clones. They were transfected with the empty vector control pAc-sgRNA-cas9 and selected in parallel under the same conditions as the target clones. The parental cells and the wt clones, WT1 and WT4, were round/oval in shape (Fig. 6a) and formed monolayer and never formed foci even at high cell density (Fig. 6h). The mutant cells were bigger and more irregular in shape with some assuming angular, spindle and elongated shapes sending out long, slender projections resembling stellate cells (Fig. 6c) whereas others have short cytoplasmic projections giving them a spiky appearance (Fig. 6d). Some of the big cells had extensive reticulate cytoplasm and/or a large cytoplasm (Fig. 6e). Some of the giant cells were apparently caught in the fusion process (Fig. 6g,k; Fig. 7; red arrows) via cell-cell contact through finger-like cytoplasmic projections or sideway apposition (Fig. 6g,k; Fig. 7, black and blue arrows). A peculiar feature of the mutant cells is the formation of double membrane vesicles (DMVs) (Fig. 6f) and multinucleated giant cells with and without DMVs (Fig. 6g). The mutant cells had lost contact inhibition and grew over each other (Fig. 6b) and clumped together (Fig. 6h) even at low cell density (Fig. 6i), suggesting a propensity to form focus. The foci were mainly made up of mononucleated cell clumps (Fig. 6j) whereas a small subset of foci were formed by multinucleated cells enclosed by a thick layer of cytoplasm encircled by single cells (Fig. 6k). Despite extensive morphological changes, the SW3 cells did not differ significantly from the parent cells or the wt cell clone, WT4, in terms of growth rates during early phase (four days) (Fig. 5c) and prolonged cultivation (12 days) (Fig. 5d) or under serum-free condition (Fig. 5e). Altogether, the results show that mono-allelic KO is sufficient to greatly reduce PHB2 expression, resulting in phenotypic switching.

### PHB2 deficiency does not inhibit ZIKV infection

**Fig. 8:**
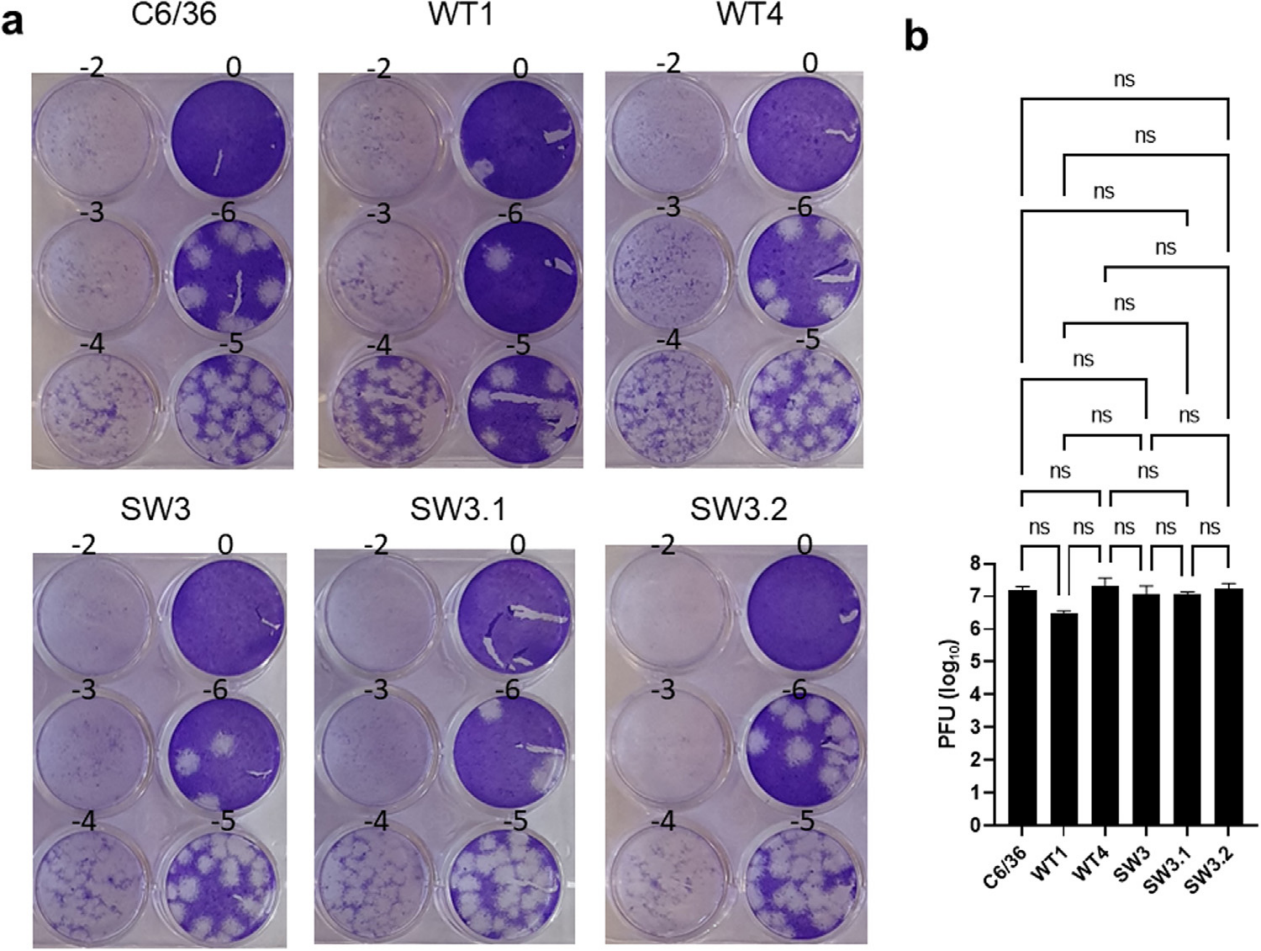
Mono-allelic knockdown of prohibitin 2 gene is insufficient to inhibit virus infection. (a) Representation of plaque assays. Titration of serial dilutions (−2 to −6; 0=no virus) of the supernatants of C6/36 cells, wild type clones (WT1; WT4) and PHB2+/- clone SW3 and sub-clones SW3.1 and SW3.2 infected with Zika virus for 48 h on A549Npro cells; (b) mean +/- SD of plaque forming unit (PFU)/ml of three independent repeats performed in duplicates. One-way ANOVA with Tukey's post-hoc test was used for the analysis of the PFU data set. A p value of <0.05 was considered statistically significant. ns=non-significant.

A previous study has proposed that PHB2 is a dengue receptor in aedine cells based on siRNA silencing of its interdependent partner PHB1 [18]. To investigate the role of PHB2 in virus infection, we infected wild type and PHB2+/- cells with ZIKV at multiplicity of infection (MOI)=1 for 48 h and then measured the virus titres secreted into the supernatants, as described previously [47]. Despite some clonal variations, there was no significant difference in virus titres between wild type cells (C6/36) and clones (WT1, WT4) and mutant clone (SW3) and sub-clones (SW3.1, SW3.2), suggesting that either PHB2 is not required for ZIKV infection or the residual 20 % PHB2 expression is sufficient for ZIKV infection (Fig. 8).

## Limitations

We have provided a toolkit for efficient knockout of difficult-to-silent genes. These PHB2-edited cell clones are valuable tools for further investigation of the roles of PHB2 in insect cell biology, virus infection and virus-host interactions. There are several limitations in this study. Firstly, we used a heterologous *Drosophila* promoter-based CRISPR-Cas9 system to express the sgRNA and Cas9. Although effective, it can be further optimized using homologous *Aedes* promoters such as the polyubiquitin promoter and the U6 promoter, *AAEL017774*. Secondly, our selection methods rely on the use of limiting dilution and/or puromycin selection, which could be a lengthy process. To speed up this process, fluorescent markers could be incorporated to allow cell sorting to improve the efficiency and accuracy of selecting KO cells. Thirdly, off-target effects pose a major obstacle in CRISPR-editing. Here, we have only detailed the use of computational tools to predict and minimize off-target hits. In the future, experimental approaches could be included to further minimize off-target effects e.g. high fidelity Cas9, paired Cas9 nickases, base editors, sgRNA length adjustment and chemical modifications, delivery methods to achieve transient peak expression. Off-targets can be assessed by whole genome sequencing (WGS) or CRISPR-edited sites enrichment followed by next-generation sequencing.

## CRediT authorship contribution statement

**Shiu-Wan Chan:** Conceptualization, Methodology, Validation, Formal analysis, Investigation, Data curation, Writing – original draft, Writing – review & editing, Visualization, Supervision, Project administration, Funding acquisition.

## Declaration of competing interests

The authors declare that they have no known competing financial interests or personal relationships that could have appeared to influence the work reported in this paper.

## Data Availability

Data will be made available on request.

## Appendix Supplementary materials

Supplementary Figure S1 Alignment of the human and insect prohibitin 2 amino acid sequences. Image, application 1

Supplementary Figure S2 Sequence alignment and phylogeny of the *Aedes albopictus* prohibitin 2 genes. Image, application 2

Supplementary Figure S3 Flanking sequences used to design primers for nested PCR of the prohibitin 2 exon 1 fragment Image, application 3

Supplementary Table S1a Target^a^ and predicted off-target^b^ sequences of GCTGTCGGTGCCGCTGCCTA in the C6/36 cell line genome^c^ Supplementary Table S1b Target^a^ and predicted off-target^b^ sequences of GGCGACCGGCTTGAAGCTGC in the C6/36 cell line genome^c^ Supplementary Table S1c Primer pair for the detection of potential off-target MNAF02000045.1^a^ Image, application 4

Supplementary Table S2 Target sequences in exon 1 of the *Aedes albopictus* prohibitin 2 gene and off-target predictions Image, application 5

Supplementary S1_File (detailed step-by-step protocols) Image, application 6
