# Supplementary material for: CRISPR-editing of the virus vector Aedes albopictus cell line C6/36, illustrated by prohibitin 2 gene knockout
Source: MethodsX. 2024 Jun 21;13:102817. doi: 10.1016/j.mex.2024.102817 (PMC11267050; doi:10.1016/j.mex.2024.102817)
Supplement: Supplementary file 1 — Supplementary Figure S1 Alignment of the human and insect prohibitin 2 amino acid sequences. [file mmc1.docx]

**1** **80**

human_PHB2_1_NP_001138303.1 **maqn-lkdlagrlp-agprgmgtalklllgagavaygvresvftvegghraiffnriggvqqdtilaeglhfripwfqyp**

human_PHB2_3_NP_001254629.1 **maqn-lkdlagrlp-agprgmgtalklllgagavaygvresvftvegghraiffnriggvqqdtilaeglhfripwfqyp**

human_PHB2_X1_XP_047284190.1 **maqn-lkdlagrlp-agprgmgtalklllgagavaygvresvftvegghraiffnriggvqqdtilaeglhfripwfqyp**

Drosophila_F_NP_001097373.1 **maqsklndlagklgkggppglgiglkvlaavgaaaygvsqslytvegghraiifsrlggiqsd-iyseglhvripwfqyp**

Drosophila_E_NP_001097372.1 **maqsklndlagklgkggppglgiglkvlaavgaaaygvsqslytvegghraiifsrlggiqsd-iyseglhvripwfqyp**

Drosophila_D_NP_001097371.1 **maqsklndlagklgkggppglgiglkvlaavgaaaygvsqslytvegghraiifsrlggiqsd-iyseglhvripwfqyp**

Foshan_KQ571446.1 **maqsklndlagkfgkggppglatglkllaavgaaayginnsmftvegghraimfnriggvgdd-ifseglhfrvpwfqyp**

Foshan_KQ562192.1 **maqsklndlagkfgkggppglatglkllaavgaaayginnsmftvegghraimfnriggvgdd-ifseglhfrvpwfqyp**

Aa-54605_GAPW01002914.1 **maqsklndlagkfgkggppglatglkllaavgaaayginnsmftvegghraimfnriggvgdd-ifseglhfrvpwfqyp**

Aa-54604_GAPW01002921.1 **maqsklndlagkfgkggppglatglkllaavgaaayginnsmftvegghraimfnriggvgdd-ifseglhfrvpwfqyp**

FPA_X1_1_XM_019686280.1 **maqsklndlagkfgkggppglatglkllaavgaaayginnsmftvegghraimfnriggvgdd-ifseglhfrvpwfqyp**

FPA_X2_1_XM_019686282.1 **maqsklndlagkfgkggppglatglkllaavgaaayginnsmftvegghraimfnriggvgdd-ifseglhfrvpwfqyp**

FPA_X3_2_XM_029858195.1 **maqsklndlagkfgkggppglatglkllaavgaaayginnsmftvegghraimfnriggvgdd-ifseglhfrvpwfqyp**

FPA_X4_1_XM_019686284.1 **maqsklndlagkfgkggppglatglkllaavgaaayginnsmftvegghraimfnriggvgdd-ifseglhfrvpwfqyp**

**81**  **160**

human_PHB2_1_NP_001138303.1 **iiydirarprkissptgskdlqmvnislrvlsrpnaqelpsmyqrlgldyeervlpsivnevlksvvakfnasqlitqra**

human_PHB2_3_NP_001254629.1 **iiydirarprkissptgskdlqmvnislrvlsrpnaqelpsmyqrlgldyeervlpsivnevlksvvakfnasqlitqra**

human_PHB2_X1_XP_047284190.1 **iiydirarprkissptgskdlqmvnislrvlsrpnaqelpsmyqrlgldyeervlpsivnevlksvvakfnasqlitqra**

Drosophila_F_NP_001097373.1 **iiydirsrprkissptgskdlqminislrvlsrpdslnlpylhkqlgvdydekvlpsicnevlksviakfnasqlitqrq**

Drosophila_E_NP_001097372.1 **iiydirsrprkissptgskdlqminislrvlsrpdslnlpylhkqlgvdydekvlpsicnevlksviakfnasqlitqrq**

Drosophila_D_NP_001097371.1 **iiydirsrprkissptgskdlqminislrvlsrpdslnlpylhkqlgvdydekvlpsicnevlksviakfnasqlitqrq**

Foshan_KQ571446.1 **ivydirsrprkissptgskdlqmvnislrvlsrpdalrlptmyrqlgldydekvlpsicnevlksvvakfnasqlitqrq**

Foshan_KQ562192.1 **ivydirsrprkissptgskdlqmvnislrvlsrpdalrlptmyrqlgldydekvlpsicnevlksvvakfnasqlitqrq**

Aa-54605_GAPW01002914.1 **ivydirsrprkissptgskdlqmvnislrvlsrpdalrlptmyrqlgldydekvlpsicnevlksvvakfnasqlitqrq**

Aa-54604_GAPW01002921.1 **ivydirsrprkissptgskdlqmvnislrvlsrpdalrlptmyrqlgldydekvlpsicnevlksvvakfnasqlitqrq**

FPA_X1_1_XM_019686280.1 **ivydirsrprkissptgskdlqmvnislrvlsrpdalrlptmyrqlgldydekvlpsicnevlksvvakfnasqlitqrq**

FPA_X2_1_XM_019686282.1 **ivydirsrprkissptgskdlqmvnislrvlsrpdalrlptmyrqlgldydekvlpsicnevlksvvakfnasqlitqrq**

FPA_X3_2_XM_029858195.1 **ivydirsrprkissptgskdlqmvnislrvlsrpdalrlptmyrqlgldydekvlpsicnevlksvvakfnasqlitqrq**

FPA_X4_1_XM_019686284.1 **ivydirsrprkissptgskdlqmvnislrvlsrpdalrlptmyrqlgldydekvlpsicnevlksvvakfnasqlitqrq**

**Novus antibody immunogen**

**161** **240**

human_PHB2_1_NP_001138303.1 **qvsllirrelterakdfslilddvaitelsfsreytaaveakqvaqqeaqraqflvekakqeqrqkivqaegeaeaakml**

human_PHB2_3_NP_001254629.1 **qvsllirrelterakdfslilddvaitelsfsreytaaveakq-------------------------------------**

human_PHB2_X1_XP_047284190.1 **qvsllirrelterakdfslilddvaitelsfsreytaaveakq-------------------------------------**

Drosophila_F_NP_001097373.1 **qvsllirkelverardfniilddvsltelsfgkeytaaieakqvaqqeaqravffverakqekqqkivqaegeaeaakml**

Drosophila_E_NP_001097372.1 **qvsllirkelverardfniilddvsltelsfgkeytaaieakqvaqqeaqravffverakqekqqkivqaegeaeaakml**

Drosophila_D_NP_001097371.1 **qvsllirkelverardfniilddvsltelsfgkeytaaieakqvaqqeaqravffverakqekqqkivqaegeaeaakml**

Foshan_KQ571446.1 **qvsllirrelverakdfniilddvsltelsfgkeytaaveskqvaqqeaqraaflverakqerqqkivqaegeaeaakml**

Foshan_KQ562192.1 **qvsllirrelverakdfniilddvsltelsfgkeytaaveskqvaqqeaqraaflverakqerqqkivqaegeaeaakml**

Aa-54605_GAPW01002914.1 **qvsllirrelverakdfniilddvsltelsfgkeytaaveskqvaqqeaqraaflverakqerqqkivqaegeaeaakml**

Aa-54604_GAPW01002921.1 **qvsllirrelverakdfniilddvsltelsfgkeytaaveskqvaqqeaqraaflverakqerqqkivqaegeaeaakml**

FPA_X1_1_XM_019686280.1 **qvsllirrelverakdfniilddvsltelsfgkeytaaveskqvaqqeaqraaflverakqerqqkivqaegeaeaakml**

FPA_X2_1_XM_019686282.1 **qvsllirrelverakdfniilddvsltelsfgkeytaaveskqvaqqeaqraaflverakqerqqkivqaegeaeaakml**

FPA_X3_2_XM_029858195.1 **qvsllirrelverakdfniilddvsltelsfgkeytaaveskqvaqqeaqraaflverakqerqqkivqaegeaeaakml**

FPA_X4_1_XM_019686284.1 **qvsllirrelverakdfniilddvsltelsfgkeytaaveskqvaqqeaqraaflverakqerqqkivqaegeaeaakml**

**241**  **320**

human_PHB2_1_NP_001138303.1 **gealsknpgyiklrkiraaqnisktiatsqnriyltadnlvlnlqdesftrgsdslikgkk**

human_PHB2_3_NP_001254629.1 **-valsknpgyiklrkiraaqnisktiatsqnriyltadnlvlnlqdesftrgsdslikgkk**

human_PHB2_X1_XP_047284190.1 **-vaqrrtgarecgkdrard**

Drosophila_F_NP_001097373.1 **glavkqnpaylklrklraaqsiartiassqnkvylsadslmlniqdsgfddmtekvyksk**

Drosophila_E_NP_001097372.1 **glavkqnpaylklrklraaqsiartiassqnkvylsadslmlniqdsgfddmtekvykigtglpkdwldarkmaskvaqp**

Drosophila_D_NP_001097371.1 **glavkqnpaylklrklraaqsiartiassqnkvylsadslmlniqdsgfddmtekvyki-----addld**

Foshan_KQ571446.1 **glavsqnpgylklrkiraaqniartiansqnrvylsanslmlnisdaefddmskrvs-skk**

Foshan_KQ562192.1 **glavsqnpgylklrkiraaqnvartiansqnrvylsanslmlnisdaefddmskkvststt---------------sakv**

Aa-54605_GAPW01002914.1 **glavsqnpgylklrkiraaqniartiansqnrvylsanslmlnisdaefddmskrvs-skk**

Aa-54604_GAPW01002921.1 **glavsqnpgylklrkiraaqnvartiansqnrvylsanslmlnisdaefddmskkvsn-k**

FPA_X1_1_XM_019686280.1 **glavsqnpgylklrkiraaqniartiansqnrvylsanslmlnisdaefddmskrvsn-kgqsrpfrvipipkasisaka**

FPA_X2_1_XM_019686282.1 **glavsqnpgylklrkiraaqniartiansqnrvylsanslmlnisdaefddmskrvsn-kgqsrpfrvipipkasisaka**

FPA_X3_2_XM_029858195.1 **glavsqnpgylklrkiraaqniartiansqnrvylsanslmlnisdaefddmskrvstdvkldhpgl**

FPA_X4_1_XM_019686284.1 **glavsqnpgylklrkiraaqniartiansqnrvylsanslmlnisdaefddmskrvsn-klgtlldk**

**321**  **350**

human_PHB2_1_NP_001138303.1

human_PHB2_3_NP_001254629.1

human_PHB2_X1_XP_047284190.1

Drosophila_F_NP_001097373.1

Drosophila_E_NP_001097372.1 **aekeknvgnvassmaermm**

Drosophila_D_NP_001097371.1

Foshan_KQ571446.1

Foshan_KQ562192.1 **eadddeqgydeslikviaeqtaerisggasq**

Aa-54605_GAPW01002914.1

Aa-54604_GAPW01002921.1

FPA_X1_1_XM_019686280.1 **eadddeqgydeslikviaeqtaerisgepev**

FPA_X2_1_XM_019686282.1 **eadddeqgydeslikviaeqtaeris-epev**

FPA_X3_2_XM_029858195.1 **-----------------------**

FPA_X4_1_XM_019686284.1 **------------------------------**

**Supplementary Figure S1 Alignment of the human and insect prohibitin 2 amino acid sequences.**

The human prohibitin 2 (PHB2) gene contains three isoforms (1, 3 and X1). The *Drosophila melanogaster* PHB2 gene contains six transcript variants with which the A, B and C variants are identical to the F variant; therefore, only transcript variants D, E, F are presented. Foshan KQ571446.1 and Foshan KQ562192.1 are two loci from the whole genome shotgun sequence of the *Aedes* *albopictus* Foshan isolate (JXUM00000000.1) that contain the PHB2 gene. Ae-54605 and Ae-54604 are putative PHB2 mRNAs derived from the transcriptome shotgun assembly loci GAPW01002914.1 and GAPW01002921.1, respectively. FPA_X1 to X4 are transcript variants from the *Ae.* *albopictus* FPA line genomic scaffold [NW_021838943.1](https://www.ncbi.nlm.nih.gov/nuccore/1696225917?report=graph&v=777467:788409). The PHB2 amino acids were aligned using Clustal Omega Multiple sequence alignment ([Clustal Omega < Multiple Sequence Alignment < EMBL-EBI](https://www.ebi.ac.uk/Tools/msa/clustalo/)) and manually adjusted for N- and C-terminal sequences. Results are depicted using MView. Conserved sequences are coloured. Accession numbers for Foshan_KQ571446.1 and Foshan_KQ562192.1 are KXJ68175 and KXJ76445, respectively. Other accession numbers can be found after the gene names. Blue arrowed line delineates the human PHB2 peptide sequence used to raise the Novus anti-PHB2 antibody in the Western blotting (Fig.5a).
