# Supplementary material for: CRISPR-editing of the virus vector Aedes albopictus cell line C6/36, illustrated by prohibitin 2 gene knockout
Source: MethodsX. 2024 Jun 21;13:102817. doi: 10.1016/j.mex.2024.102817 (PMC11267050; doi:10.1016/j.mex.2024.102817)
Supplement: Supplementary file 2 — Supplementary Figure S2 Sequence alignment and phylogeny of the Aedes albopictus prohibitin 2 genes. [file mmc2.docx]

**a**

**1** **120**

Foshan_KQ571446.1 **atggctcagagcaaactgaacgatttggccggaaaattcggtaagggtggaccacccggattggcgaccggcctgaagctgctggcagctgtcggtgccgctgcctacggaatcaacaat**

Foshan_JXUM01152402.1 **atggctcagagcaaactgaacgatttggccggaaaattcggtaagggtggaccacccggattggcgaccggcctgaagctgctggcagctgtcggtgccgctgcctacggaatcaacaat**

FPA_SWKZ01010266.1 **atggctcagagcaaactgaacgatttggccggaaaattcggtaagggtggaccacccggattggcgaccggcctgaagctgctggcagctgtcggtgccgctgcctacggaatcaacaat**

FPA_SWKZ01015888.1 **atggctcagagcaaactgaacgatttggccggaaaattcggtaagggtggaccacccggattggcgaccggcctgaagctgctggcagctgtcggtgccgctgcctacggaatcaacaat**

FPA_SWKY01000073.1_1 **atggctcagagcaaactgaacgatttggccggaaaattcggtaagggtggaccacccggattggcgaccggcctgaagctgctggcagctgtcggtgccgctgcctacggaatcaacaat**

FPA_SWKY01000073.1_2 **atggctcagagcaaactgaacgatttggccggaaaattcggtaagggtggaccacccggattggcgaccggcctgaagctgctggcagctgtcggtgccgctgcctacggaatcaacaat**

Rimini **atggctcagagcaaactgaacgatttggccggaaaattcggtaagggtggaccacccggattggcgaccggcctgaagctgctggcagctgtcggtgccgctgcctacggaatcaacaat**

Rimini_2_602-792 **------------------------------------------------------------------------------------------------------------------------**

Rimini_2_131-274 **------------------------------------------------------------------------------------------------------------------------**

C636_MNAF02000396.1 **atggctcagagcaaactgaacgatttggccggaaaattcggcaagggtggaccacccggtttggcgaccggcttgaagctgctggcagctgtcggtgccgctgcctacggtatcaacaat**

C636_MNAF02001030.1 **atggctcagagcaaactgaacgatttggccggaaaattcggcaagggtggtccacccggattggcgaccggcttgaagctgctggcagctgtcggtgccgctgcctacggaattaacaat**

Foshan_KQ562192.1 **atggctcagagcaaactgaacgatttggccggaaaattcggcaaaggtggtccacccggattggcgaccggcctgaagctgctggcagctgtcggtgccgctgcctacggaattaacaat**

Foshan_JXUM01062273.1 **atggctcagagcaaactgaacgatttggccggaaaattcggcaaaggtggtccacccggattggcgaccggcctgaagctgctggcagctgtcggtgccgctgcctacggaattaacaat**

FPA_SWKY01000135.1 **atggctcagagcaaactgaacgatttggccggaaaattcggcaaaggtggtccacccggattggcgaccggcctgaagctgctggcagctgtcggtgccgctgcctacggaattaacaat**

**121**  **240**

Foshan_KQ571446.1 **tccatgttcacagtcgaaggtggccaccgtgcaatcatgtttaaccgaattggcggagtcggtgatgacatcttcagtgaaggactacacttccgagttccgtggttccagtacccgatt**

Foshan_JXUM01152402.1 **tccatgttcacagtcgaaggtggccaccgtgcaatcatgtttaaccgaattggcggagtcggtgatgacatcttcagtgaaggactacacttccgagttccgtggttccagtacccgatt**

FPA_SWKZ01010266.1 **tccatgttcacagtcgaaggtggccaccgtgcaatcatgtttaaccgaattggcggagtcggtgatgacatcttcagtgaaggactacacttccgagttccgtggttccagtacccgatt**

FPA_SWKZ01015888.1 **tccatgttcacagtcgaaggtggccaccgtgcaatcatgtttaaccgaattggcggagtcggtgatgacatcttcagtgaaggactacacttccgagttccgtggttccagtacccgatt**

FPA_SWKY01000073.1_1 **tccatgttcacagtcgaaggtggccaccgtgcaatcatgtttaaccgaattggcggagtcggtgatgacatcttcagtgaaggactacacttccgagttccgtggttccagtacccgatt**

FPA_SWKY01000073.1_2 **tccatgttcacagtcgaaggtggccaccgtgcaatcatgtttaaccgaattggcggagtcggtgatgacatcttcagtgaaggactacacttccgagttccgtggttccagtacccgatt**

Rimini **tccatgttcacagtcgaaggtggccaccgtgcaatcatgtttaaccgaattggcggagtcggtgatgacatcttcagtgaaggactacacttccgagttccgtggttccagtacccgatt**

Rimini_2_602-792 **------------------------------------------------------------------------------------------------------------------------**

Rimini_2_131-274 **----------cagttgagggtggccaccgtgcaatcatgttcaaccgaattggcggagtcggtgatgacatcttcagtgaagggctgcacttccgagttccgtggttccagtacccgatt**

C636_MNAF02000396.1 **tccatgttcacagttgagggtggccaccgtgcaatcatgttcaaccgaattggtggagtcggcgatgacatcttcagtgaaggactacacttccgagttccgtggttccagtacccgatt**

C636_MNAF02001030.1 **tccatgttcacagttgagggtggccaccgtgcaatcatgttcaaccgaattggcggagtcggcgatgacatcttcagtgaaggactacacttccgagttccgtggttccagtacccgatt**

Foshan_KQ562192.1 **tccatgttcacagtcgagggtggccaccgtgcaatcatgttcaaccgaattggcggagtcggtgatgacatcttcagtgaagggctgcacttccgagttccgtggttccagtacccgatt**

Foshan_JXUM01062273.1 **tccatgttcacagtcgagggtggccaccgtgcaatcatgttcaaccgaattggcggagtcggtgatgacatcttcagtgaagggctgcacttccgagttccgtggttccagtacccgatt**

FPA_SWKY01000135.1 **tccatgttcacagtcgagggtggccaccgtgcaatcatgttcaaccgaattggcggagtcggtgatgacatcttcagtgaagggctgcacttccgagttccgtggttccagtacccgatt**

**241**  **360**

Foshan_KQ571446.1 **gtgtacgacatccgttcccgcccgaggaaaatctcgtccccaactggttcgaaggatttgcagatggtcaatatctcgctccgagtgttgtcccgcccagatgccctccgattgcccacg**

Foshan_JXUM01152402.1 **gtgtacgacatccgttcccgcccgaggaaaatctcgtccccaactggttcgaaggatttgcagatggtcaatatctcgctccgagtgttgtcccgcccagatgccctccgattgcccacg**

FPA_SWKZ01010266.1 **gtgtacgacatccgttcccgcccgaggaaaatctcgtccccaactggttcgaaggatttgcagatggtcaatatctcgctccgagtgttgtcccgcccagatgccctccgattgcccacg**

FPA_SWKZ01015888.1 **gtgtacgacatccgttcccgcccgaggaaaatctcgtccccaactggttcgaaggatttgcagatggtcaatatctcgctccgagtgttgtcccgcccagatgccctccgattgcccacg**

FPA_SWKY01000073.1_1 **gtgtacgacatccgttcccgcccgaggaaaatctcgtccccaactggttcgaaggatttgcagatggtcaatatctcgctccgagtgttgtcccgcccagatgccctccgattgcccacg**

FPA_SWKY01000073.1_2 **gtgtacgacatccgttcccgcccgaggaaaatctcgtccccaactggttcgaaggatttgcagatggtcaatatctcgctccgagtgttgtcccgcccagatgccctccgattgcccacg**

Rimini **gtgtacgacatccgttcccgcccgaggaaaatctcgtccccaactggttcgaaggatttgcagatggtcaatatctcgctccgagtgttgtcccgcccagatgccctccgattgcccacg**

Rimini_2_602-792 **------------------------------------------------------------------------------------------------------------------------**

Rimini_2_131-274 **gtgtacgacatccgttcccgccctaggaaaatct--------------------------------------------------------------------------------------**

C636_MNAF02000396.1 **gtgtacgatatccgttcccgccctaggaaaatctcgtccccaactggttcgaaggatttgcagatggtcaacatttcgctccgagtgctatcccgcccagatgccctccgattgcccaca**

C636_MNAF02001030.1 **gtgtacgatatccgttcccgccctaggaaaatctcgtccccaactggttccaaggatttgcagatggtcaacatttcgctccgagtgctgtcccgcccagatgccctacgattgcccacg**

Foshan_KQ562192.1 **gtgtacgatatccgttcccgccccaggaaaatctcgtccccaactggttcgaaggatttgcagatggtcaacatttcactccgagtgctgtcccgcccagatgccctccgattgcccacg**

Foshan_JXUM01062273.1 **gtgtacgatatccgttcccgccccaggaaaatctcgtccccaactggttcgaaggatttgcagatggtcaacatttcactccgagtgctgtcccgcccagatgccctccgattgcccacg**

FPA_SWKY01000135.1 **gtgtacgatatccgttcccgccccaggaaaatctcgtccccaactggttcgaaggatttgcagatggtcaacatttcactccgagtgctgtcccgcccagatgccctccgattgcccacg**

**361** **480**

Foshan_KQ571446.1 **atgtaccgtcagcttggattggactacgacgaaaaggtgctgccatcgatttgcaacgaagtgctcaagagtgtggtcgccaagttcaatgcctcccagctgattacccagcgtcagcag**

Foshan_JXUM01152402.1 **atgtaccgtcagcttggattggactacgacgaaaaggtgctgccatcgatttgcaacgaagtgctcaagagtgtggtcgccaagttcaatgcctcccagctgattacccagcgtcagcag**

FPA_SWKZ01010266.1 **atgtaccgtcagcttggattggactacgacgaaaaggtgctgccatcgatttgcaacgaagtgctcaagagtgtggtcgccaagttcaatgcctcccagctgattacccagcgtcagcag**

FPA_SWKZ01015888.1 **atgtaccgtcagcttggattggactacgacgaaaaggtgctgccatcgatttgcaacgaagtgctcaagagtgtggtcgccaagttcaatgcctcccagctgattacccagcgtcagcag**

FPA_SWKY01000073.1_1 **atgtaccgtcagcttggattggactacgacgaaaaggtgctgccatcgatttgcaacgaagtgctcaagagtgtggtcgccaagttcaatgcctcccagctgattacccagcgtcagcag**

FPA_SWKY01000073.1_2 **atgtaccgtcagcttggattggactacgacgaaaaggtgctgccatcgatttgcaacgaagtgctcaagagtgtggtcgccaagttcaatgcctcccagctgattacccagcgtcagcag**

Rimini **atgtaccgtcagcttggattggactacgacgaaaaggtgctgccatcgatttgcaacgaagtgctcaagagtgtggtcgccaagttcaatgcctcccagctgattacccagcgtcagcag**

Rimini_2_602-792 **------------------------------------------------------------------------------------------------------------------------**

Rimini_2_131-274 **------------------------------------------------------------------------------------------------------------------------**

C636_MNAF02000396.1 **atgtaccgtcagctcggattggattacgacgaaaaggtgctgccatcgatttgcaacgaagtgctcaagagtgtggtggccaagttcaacgcctcgcagctgattacccagcgtcagcag**

C636_MNAF02001030.1 **atgtaccgtcagctcggattggattacgacgaaaaggtgctgccatcgatttgcaacgaagtgctcaagagtgtggtggccaagttcaacgcctcgcagctgattacccagcgtcagcag**

Foshan_KQ562192.1 **atgtaccgtcagctcggattggattacgacgaaaaggtgctgccgtcgatttgcaacgaagtgctcaagagtgtggtggccaagttcaacgcctcgcagctgattacccagcgtcagcag**

Foshan_JXUM01062273.1 **atgtaccgtcagctcggattggattacgacgaaaaggtgctgccgtcgatttgcaacgaagtgctcaagagtgtggtggccaagttcaacgcctcgcagctgattacccagcgtcagcag**

FPA_SWKY01000135.1 **atgtaccgtcagctcggattggattacgacgaaaaggtgctgccgtcgatttgcaacgaagtgctcaagagtgtggtggccaagttcaacgcctcgcagctgattacccagcgtcagcag**

**481**  **600**

Foshan_KQ571446.1 **gtttcgctgctgatccgccgggagctggtggagcgcgcgaaggatttcaacatcattctggatgacgtttcgcttacggagctcagctttggcaaggaatatacggcggccgtcgaaagc**

Foshan_JXUM01152402.1 **gtttcgctgctgatccgccgggagctggtggagcgcgcgaaggatttcaacatcattctggatgacgtttcgcttacggagctcagctttggcaaggaatatacggcggccgtcgaaagc**

FPA_SWKZ01010266.1 **gtttcgctgctgatccgccgggagctggtggagcgcgcgaaggatttcaacatcattctggatgacgtttcgcttacggagctcagctttggcaaggaatatacggcggccgtcgaaagc**

FPA_SWKZ01015888.1 **gtttcgctgcttatccgccgggagctggtggagcgcgcgaaggatttcaacatcattctggatgacgtttcgcttacggagctcagctttggcaaggaatatacggcggccgtcgaaagc**

FPA_SWKY01000073.1_1 **gtttcgctgctgatccgccgggagctggtggagcgcgcgaaggatttcaacatcattctggatgacgtttcgcttacggagctcagctttggcaaggaatatacggcggccgtcgaaagc**

FPA_SWKY01000073.1_2 **gtttcgctgctgatccgccgggagctggtggagcgcgcgaaggatttcaacatcattctggatgacgtttcgcttacggagctcagctttggcaaggaatatacggcggccgtcgaaagc**

Rimini **gtttcgctgctgatccgccgggagctggtggagcgcgcgaaggatttcaacatcattctggatgacgtttcgcttacggagctcagctttggcaaggaatatacggcggccgtcgaaagc**

Rimini_2_602-792 **------------------------------------------------------------------------------------------------------------------------**

Rimini_2_131-274 **------------------------------------------------------------------------------------------------------------------------**

C636_MNAF02000396.1 **gtttcgctgctgatccgccgggagttggtggagcgcgcaaaggatttcaacatcattctggatgacgtttcgcttacggagctcagcttcggcaaggaatacacggcggccgtcgaaagc**

C636_MNAF02001030.1 **gtttcgctgctgatccgccgggagttggtggagcgcgcgaaggatttcaacatcattctggatgacgtttcgctgacggagctcagcttcggcaaggaatacacggcggccgtcgaaagc**

Foshan_KQ562192.1 **gtttcgctgctgatccgccgggagttggtggagcgcgcgaaggatttcaacatcattctggatgacgtttcgctgacggagctcagcttcggcaaggaatacacggcggccgtcgaaagc**

Foshan_JXUM01062273.1 **gtttcgctgctgatccgccgggagttggtggagcgcgcgaaggatttcaacatcattctggatgacgtttcgctgacggagctcagcttcggcaaggaatacacggcggccgtcgaaagc**

FPA_SWKY01000135.1 **gtttcgctgctgatccgccgggagttggtggagcgcgcgaaggatttcaacatcattctggatgacgtttcgctgacggagctcagcttcggcaaggaatacacggcggccgtcgaaagc**

**601**  **720**

Foshan_KQ571446.1 **aaacaagtggcccagcaggaagcccagcgggctgctttcctggtggagcgtgccaagcaggaacgtcaacagaagattgtccaggccgagggagaagctgaggccgcaaaaatgttgggt**

Foshan_JXUM01152402.1 **aaacaagtggcccagcaggaagcccagcgggctgctttcctggtggagcgtgccaagcaggaacgtcaacagaagattgtccaggccgagggagaagctgaggccgcaaaaatgttgggt**

FPA_SWKZ01010266.1 **aaacaagtggcccagcaggaagcccagcgggctgctttcctggtggagcgtgccaagcaggaacgtcaacagaagattgtccaggccgagggagaagctgaggccgcaaaaatgttgggt**

FPA_SWKZ01015888.1 **aaacaagtggcccagcaggaagcccagcgggctgctttcctggtggagcgtgccaagcaggaacgtcaacagaagattgtccaggccgagggagaagctgaggccgcaaaaatgttgggt**

FPA_SWKY01000073.1_1 **aaacaagtggcccagcaggaagcccagcgggctgctttcctggtggagcgtgccaagcaggaacgtcaacagaagattgtccaggccgagggagaagctgaggccgcgaaaatgttgggt**

FPA_SWKY01000073.1_2 **aaacaagtggcccagcaggaagcccagcgggctgctttcctggtggagcgtgccaagcaggaacgtcaacagaaaattgtccaggccgagggagaagctgaggccgcgaaaatgttgggt**

Rimini **aaacaagtggcccagcaggaagcccagcgggctgctttcctggtggagcgtgccaagcaggaacgtcaacagaaaattgtccaggccgagggagaagctgaggccgcgaaaatgttgggt**

Rimini_2_602-792 **-aacaggttgcccagcaggaagcacagcgggccgctttcctggtggagcgtgccaagcaggaacgtcagcagaagattgtccaagctgagggagaagccgaggccgcgaaaatgttgggt**

Rimini_2_131-274 **------------------------------------------------------------------------------------------------------------------------**

C636_MNAF02000396.1 **aaacaggttgcccagcaggaagcccagcgggccgctttcctggtggagcgtgccaagcaggaacgtcagcagaagattgttcaagctgagggagaagccgaggccgcaaaaatgttgggt**

C636_MNAF02001030.1 **aaacaggttgcccagcaggaagcccagcgggccgctttcctggtggagcgtgccaagcaggaacgtcagcagaagattgtccaagctgagggagaagccgaggccgcgaaaatgttgggt**

Foshan_KQ562192.1 **aaacaggttgcccagcaggaagcccagcgggccgctttcctggtggagcgtgccaagcaggaacgtcagcagaagattgtccaagctgagggagaagccgaggccgcgaaaatgttgggt**

Foshan_JXUM01062273.1 **aaacaggttgcccagcaggaagcccagcgggccgctttcctggtggagcgtgccaagcaggaacgtcagcagaagattgtccaagctgagggagaagccgaggccgcgaaaatgttgggt**

FPA_SWKY01000135.1 **aaacaggttgcccagcaggaagcccagcgggccgctttcctggtggagcgtgccaagcaggaacgtcagcagaagattgtccaagctgagggagaagccgaggccgcgaaaatgttgggt**

**721** **840**

Foshan_KQ571446.1 **ctggccgttagtcaaaaccctggctacctaaagctcaggaagatccgcgccgcacagaacattgctcgaacgatcgccaactcgcagaaccgtgtctacctctccgccaacagtctgatg**

Foshan_JXUM01152402.1 **ctggccgttagtcaaaaccctggctacctaaagctcaggaagatccgcgccgcacagaacattgctcgaacgatcgccaactcgcagaaccgtgtctacctctccgccaacagtctgatg**

FPA_SWKZ01010266.1 **ctggccgttagtcaaaaccctggctacctaaagctcaggaagatccgcgccgcacagaacattgctcgaacgatcgccaactcgcagaaccgtgtctacctctccgccaacagtctgatg**

FPA_SWKZ01015888.1 **ctggccgttagtcaaaaccctggctacctaaagctcaggaagatccgcgccgcacagaacattgctcgaacgatcgccaactcgcagaaccgtgtctacctctccgccaacagtctgatg**

FPA_SWKY01000073.1_1 **ctggccgttagtcaaaaccctggctacctgaagctcaggaagatccgcgccgcacagaacattgctcgaacgatcgccaactcgcagaaccgtgtctacctctccgccaacagtctgatg**

FPA_SWKY01000073.1_2 **ctggccgttagtcaaaaccctggctacctgaagctcaggaagatccgcgccgcacagaacattgctcgaacgatcgccaactcgcagaaccgtgtctacctctccgccaacagtctgatg**

Rimini **ctggccgttagtcaaaaccctggctacctgaagctcaggaagatccgcgccgcacagaacattgctcgaacgatcgccaactcgcagaaccgtgtctacctctccgccaacagtctgatg**

Rimini_2_602-792 **ctggccgttagtcaaaatcctggttacctaaagctcaggaagatccgcgccgcacagaacgttgctcgaacg------------------------------------------------**

Rimini_2_131-274 **------------------------------------------------------------------------------------------------------------------------**

C636_MNAF02000396.1 **ctggccgttagtcaaaatcctggctacctgaagctcaggaagattcgcgccgcacagaacgttgcccgaacgatcgccaactcgcagaaccgtgtctacctctccgccaacagtttgatg**

C636_MNAF02001030.1 **ctggccgttagtcaaaatcccggctacctgaagctcaggaagatccgcgccgcacagaacgttgctcgaacgatcgccaactcgcagaaccgtgtctacctctccgccaacagtctgatg**

Foshan_KQ562192.1 **ctggccgttagtcaaaatcccggctacctgaagctcaggaagatccgcgccgcacagaacgttgctcgaacgatcgccaactcgcagaaccgtgtctacctctccgccaacagtctgatg**

Foshan_JXUM01062273.1 **ctggccgttagtcaaaatcccggctacctgaagctcaggaagatccgcgccgcacagaacgttgctcgaacgatcgccaactcgcagaaccgtgtctacctctccgccaacagtctgatg**

FPA_SWKY01000135.1 **ctggccgttagtcaaaatcccggctacctgaagctcaggaagatccgcgccgcacagaacgttgctcgaacgatcgccaactcgcagaaccgtgtctacctctccgccaacagtctgatg**

**841**  **960**

Foshan_KQ571446.1 **ttgaacatttccgatgccgagttcgacgacatgtccaagagggtttcaagtaagaaatga------------------------------------------------------------**

Foshan_JXUM01152402.1 **ttgaacatttccgatgccgagttcgacgacatgtccaagagggtttcaagtaagaaatga------------------------------------------------------------**

FPA_SWKZ01010266.1 **ttgaacatttccgatgccgagttcgacgacatgtccaagagggtttcaagtaagaaatga------------------------------------------------------------**

FPA_SWKZ01015888.1 **ttgaacatttccgatgccgagttcgacgacatgtccaagagggtttcaagtaagaaatga------------------------------------------------------------**

FPA_SWKY01000073.1_1 **ttgaacatttccgatgccgagttcgacgacatgtccaagagggtttcaagtaagaaatga------------------------------------------------------------**

FPA_SWKY01000073.1_2 **ttgaacatttccgatgccgagttcgacgacatgtccaagagggtttcaagtaagaaatga------------------------------------------------------------**

Rimini **ttgaacatttccgatgccgagttcgacgacatgtccaagagggtttcaagtaagaaatga------------------------------------------------------------**

Rimini_2_602-792 **------------------------------------------------------------------------------------------------------------------------**

Rimini_2_131-274 **------------------------------------------------------------------------------------------------------------------------**

C636_MNAF02000396.1 **ttgaacatttccgatgccgagttcgacgacatgtccaagaaagtttcaagtaagaaatga------------------------------------------------------------**

C636_MNAF02001030.1 **ttgaacatttccgatgccgagttcgacgacatgtccaagaaagtttcaagtaagaaatga------------------------------------------------------------**

Foshan_KQ562192.1 **ttgaacatttccgatgccgagttcgacgacatgtccaagaaagtttcaacctccaccacttctgcgaaagtagaagctgatgacgacgaacagggatatgacgagagtctgatcaaagtt**

Foshan_JXUM01062273.1 **ttgaacatttccgatgccgagttcgacgacatgtccaagaaagtttcaagtaagaaatga------------------------------------------------------------**

FPA_SWKY01000135.1 **ttgaacatttccgatgccgagttcgacgacatgtccaagaaagtttcaagtaagaaatga------------------------------------------------------------**

**961**  **1008**

Foshan_KQ571446.1 **------------------------------------------------**

Foshan_JXUM01152402.1 **------------------------------------------------**

FPA_SWKZ01010266.1 **------------------------------------------------**

FPA_SWKZ01015888.1 **------------------------------------------------**

FPA_SWKY01000073.1_1 **------------------------------------------------**

FPA_SWKY01000073.1_2 **------------------------------------------------**

Rimini **------------------------------------------------**

Rimini_2_602-792 **------------------------------------------------**

Rimini_2_131-274 **------------------------------------------------**

C636_MNAF02000396.1 **------------------------------------------------**

C636_MNAF02001030.1 **------------------------------------------------**

Foshan_KQ562192.1 **atcgcggaacagacggctgaaaggatcagtggtggtgctagccagtaa**

Foshan_JXUM01062273.1 **------------------------------------------------**

FPA_SWKY01000135.1 **------------------------------------------------**

**b**


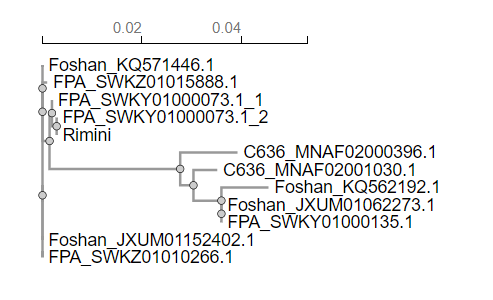

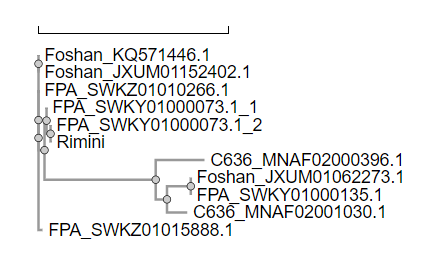


phylogenetic tree with Foshan_KQ562192.1 removed

**c**

**1**  **80**

Foshan_KQ571446.1 **MAQSKLNDLAGKFGKGGPPGLATGLKLLAAVGAAAYGINNSMFTVEGGHRAIMFNRIGGVGDDIFSEGLHFRVPWFQYPI**

Foshan_JXUM01152402.1 **MAQSKLNDLAGKFGKGGPPGLATGLKLLAAVGAAAYGINNSMFTVEGGHRAIMFNRIGGVGDDIFSEGLHFRVPWFQYPI**

FPA_SWKZ01010266.1 **MAQSKLNDLAGKFGKGGPPGLATGLKLLAAVGAAAYGINNSMFTVEGGHRAIMFNRIGGVGDDIFSEGLHFRVPWFQYPI**

FPA_SWKZ01015888.1 **MAQSKLNDLAGKFGKGGPPGLATGLKLLAAVGAAAYGINNSMFTVEGGHRAIMFNRIGGVGDDIFSEGLHFRVPWFQYPI**

FPA_SWKY01000073.1_1 **MAQSKLNDLAGKFGKGGPPGLATGLKLLAAVGAAAYGINNSMFTVEGGHRAIMFNRIGGVGDDIFSEGLHFRVPWFQYPI**

FPA_SWKY01000073.1_2 **MAQSKLNDLAGKFGKGGPPGLATGLKLLAAVGAAAYGINNSMFTVEGGHRAIMFNRIGGVGDDIFSEGLHFRVPWFQYPI**

Rimini **MAQSKLNDLAGKFGKGGPPGLATGLKLLAAVGAAAYGINNSMFTVEGGHRAIMFNRIGGVGDDIFSEGLHFRVPWFQYPI**

Rimini_2_602-792 **--------------------------------------------------------------------------------**

Rimini_2_131-274 **--------------------------------------------VEGGHRAIMFNRIGGVGDDIFSEGLHFRVPWFQYPI**

C636_MNAF02000396.1 **MAQSKLNDLAGKFGKGGPPGLATGLKLLAAVGAAAYGINNSMFTVEGGHRAIMFNRIGGVGDDIFSEGLHFRVPWFQYPI**

C636_MNAF02001030.1 **MAQSKLNDLAGKFGKGGPPGLATGLKLLAAVGAAAYGINNSMFTVEGGHRAIMFNRIGGVGDDIFSEGLHFRVPWFQYPI**

Foshan_KQ562192.1 **MAQSKLNDLAGKFGKGGPPGLATGLKLLAAVGAAAYGINNSMFTVEGGHRAIMFNRIGGVGDDIFSEGLHFRVPWFQYPI**

Foshan_JXUM01062273.1 **MAQSKLNDLAGKFGKGGPPGLATGLKLLAAVGAAAYGINNSMFTVEGGHRAIMFNRIGGVGDDIFSEGLHFRVPWFQYPI**

FPA_SWKY01000135.1 **MAQSKLNDLAGKFGKGGPPGLATGLKLLAAVGAAAYGINNSMFTVEGGHRAIMFNRIGGVGDDIFSEGLHFRVPWFQYPI**

**81**   **160**

Foshan_KQ571446.1 **VYDIRSRPRKISSPTGSKDLQMVNISLRVLSRPDALRLPTMYRQLGLDYDEKVLPSICNEVLKSVVAKFNASQLITQRQQ**

Foshan_JXUM01152402.1 **VYDIRSRPRKISSPTGSKDLQMVNISLRVLSRPDALRLPTMYRQLGLDYDEKVLPSICNEVLKSVVAKFNASQLITQRQQ**

FPA_SWKZ01010266.1 **VYDIRSRPRKISSPTGSKDLQMVNISLRVLSRPDALRLPTMYRQLGLDYDEKVLPSICNEVLKSVVAKFNASQLITQRQQ**

FPA_SWKZ01015888.1 **VYDIRSRPRKISSPTGSKDLQMVNISLRVLSRPDALRLPTMYRQLGLDYDEKVLPSICNEVLKSVVAKFNASQLITQRQQ**

FPA_SWKY01000073.1_1 **VYDIRSRPRKISSPTGSKDLQMVNISLRVLSRPDALRLPTMYRQLGLDYDEKVLPSICNEVLKSVVAKFNASQLITQRQQ**

FPA_SWKY01000073.1_2 **VYDIRSRPRKISSPTGSKDLQMVNISLRVLSRPDALRLPTMYRQLGLDYDEKVLPSICNEVLKSVVAKFNASQLITQRQQ**

Rimini **VYDIRSRPRKISSPTGSKDLQMVNISLRVLSRPDALRLPTMYRQLGLDYDEKVLPSICNEVLKSVVAKFNASQLITQRQQ**

Rimini_2_602-792 **--------------------------------------------------------------------------------**

Rimini_2_131-274 **VYDIRSRPRKI---------------------------------------------------------------------**

C636_MNAF02000396.1 **VYDIRSRPRKISSPTGSKDLQMVNISLRVLSRPDALRLPTMYRQLGLDYDEKVLPSICNEVLKSVVAKFNASQLITQRQQ**

C636_MNAF02001030.1 **VYDIRSRPRKISSPTGSKDLQMVNISLRVLSRPDALRLPTMYRQLGLDYDEKVLPSICNEVLKSVVAKFNASQLITQRQQ**

Foshan_KQ562192.1 **VYDIRSRPRKISSPTGSKDLQMVNISLRVLSRPDALRLPTMYRQLGLDYDEKVLPSICNEVLKSVVAKFNASQLITQRQQ**

Foshan_JXUM01062273.1 **VYDIRSRPRKISSPTGSKDLQMVNISLRVLSRPDALRLPTMYRQLGLDYDEKVLPSICNEVLKSVVAKFNASQLITQRQQ**

FPA_SWKY01000135.1 **VYDIRSRPRKISSPTGSKDLQMVNISLRVLSRPDALRLPTMYRQLGLDYDEKVLPSICNEVLKSVVAKFNASQLITQRQQ**

**161** **240**

Foshan_KQ571446.1 **VSLLIRRELVERAKDFNIILDDVSLTELSFGKEYTAAVESKQVAQQEAQRAAFLVERAKQERQQKIVQAEGEAEAAKMLG**

Foshan_JXUM01152402.1 **VSLLIRRELVERAKDFNIILDDVSLTELSFGKEYTAAVESKQVAQQEAQRAAFLVERAKQERQQKIVQAEGEAEAAKMLG** FPA_SWKZ01010266.1 **VSLLIRRELVERAKDFNIILDDVSLTELSFGKEYTAAVESKQVAQQEAQRAAFLVERAKQERQQKIVQAEGEAEAAKMLG**

FPA_SWKZ01015888.1 **VSLLIRRELVERAKDFNIILDDVSLTELSFGKEYTAAVESKQVAQQEAQRAAFLVERAKQERQQKIVQAEGEAEAAKMLG**

FPA_SWKY01000073.1_1 **VSLLIRRELVERAKDFNIILDDVSLTELSFGKEYTAAVESKQVAQQEAQRAAFLVERAKQERQQKIVQAEGEAEAAKMLG**

FPA_SWKY01000073.1_2 **VSLLIRRELVERAKDFNIILDDVSLTELSFGKEYTAAVESKQVAQQEAQRAAFLVERAKQERQQKIVQAEGEAEAAKMLG**

Rimini **VSLLIRRELVERAKDFNIILDDVSLTELSFGKEYTAAVESKQVAQQEAQRAAFLVERAKQERQQKIVQAEGEAEAAKMLG**

Rimini_2_602-792 **-----------------------------------------QVAQQEAQRAAFLVERAKQERQQKIVQAEGEAEAAKMLG**

Rimini_2_131-274 **--------------------------------------------------------------------------------**

C636_MNAF02000396.1 **VSLLIRRELVERAKDFNIILDDVSLTELSFGKEYTAAVESKQVAQQEAQRAAFLVERAKQERQQKIVQAEGEAEAAKMLG**

C636_MNAF02001030.1 **VSLLIRRELVERAKDFNIILDDVSLTELSFGKEYTAAVESKQVAQQEAQRAAFLVERAKQERQQKIVQAEGEAEAAKMLG**

Foshan_KQ562192.1 **VSLLIRRELVERAKDFNIILDDVSLTELSFGKEYTAAVESKQVAQQEAQRAAFLVERAKQERQQKIVQAEGEAEAAKMLG**

Foshan_JXUM01062273.1 **VSLLIRRELVERAKDFNIILDDVSLTELSFGKEYTAAVESKQVAQQEAQRAAFLVERAKQERQQKIVQAEGEAEAAKMLG**

FPA_SWKY01000135.1 **VSLLIRRELVERAKDFNIILDDVSLTELSFGKEYTAAVESKQVAQQEAQRAAFLVERAKQERQQKIVQAEGEAEAAKMLG**

**241**  *** * 299**

Foshan_KQ571446.1 **LAVSQNPGYLKLRKIRAAQNIARTIANSQNRVYLSANSLMLNISDAEFDDMSKRVSSKK---------------------**

Foshan_JXUM01152402.1 **LAVSQNPGYLKLRKIRAAQNIARTIANSQNRVYLSANSLMLNISDAEFDDMSKRVSSKK---------------------**

FPA_SWKZ01010266.1 **LAVSQNPGYLKLRKIRAAQNIARTIANSQNRVYLSANSLMLNISDAEFDDMSKRVSSKK---------------------**

FPA_SWKZ01015888.1 **LAVSQNPGYLKLRKIRAAQNIARTIANSQNRVYLSANSLMLNISDAEFDDMSKRVSSKK---------------------**

FPA_SWKY01000073.1_1 **LAVSQNPGYLKLRKIRAAQNIARTIANSQNRVYLSANSLMLNISDAEFDDMSKRVSSKK---------------------**

FPA_SWKY01000073.1_2 **LAVSQNPGYLKLRKIRAAQNIARTIANSQNRVYLSANSLMLNISDAEFDDMSKRVSSKK---------------------**

Rimini **LAVSQNPGYLKLRKIRAAQNIARTIANSQNRVYLSANSLMLNISDAEFDDMSKRVSSKK---------------------**

Rimini_2_602-792 **LAVSQNPGYLKLRKIRAAQNVART--------------------------------------------------------**

Rimini_2_131-274 **--------------------------------------------------------------------------------**

C636_MNAF02000396.1 **LAVSQNPGYLKLRKIRAAQNVARTIANSQNRVYLSANSLMLNISDAEFDDMSKKVSSKK---------------------**

C636_MNAF02001030.1 **LAVSQNPGYLKLRKIRAAQNVARTIANSQNRVYLSANSLMLNISDAEFDDMSKKVSSKK---------------------**

Foshan_KQ562192.1 **LAVSQNPGYLKLRKIRAAQNVARTIANSQNRVYLSANSLMLNISDAEFDDMSKKVSTSTTSAKVEADDDEQGYDESLIKV**

Foshan_JXUM01062273.1 **LAVSQNPGYLKLRKIRAAQNVARTIANSQNRVYLSANSLMLNISDAEFDDMSKKVSSKK---------------------**

FPA_SWKY01000135.1 **LAVSQNPGYLKLRKIRAAQNVARTIANSQNRVYLSANSLMLNISDAEFDDMSKKVSSKK---------------------**

**321**  **335**

Foshan_KQ571446.1 **---------------**

Foshan_JXUM01152402.1 **---------------**

FPA_SWKZ01010266.1 **---------------**

FPA_SWKZ01015888.1 **---------------**

FPA_SWKY01000073.1_1 **---------------**

FPA_SWKY01000073.1_2 **---------------**

Rimini **---------------**

Rimini_2_602-792 **---------------**

Rimini_2_131-274 **---------------**

C636_MNAF02000396.1 **---------------**

C636_MNAF02001030.1 **---------------**

Foshan_KQ562192.1 **IAEQTAERISGGASQ**

Foshan_JXUM01062273.1 **---------------**

FPA_SWKY01000135.1 **---------------**

**Supplementary Figure S2 Sequence alignment and phylogeny of the *Aedes albopictus* prohibitin 2 genes.**

The Foshan KQ571446.1 locus containing the prohibitin 2 (PHB2) gene was used as the input sequence to BLASTn against the ‘whole genome shotgun sequences of *Aedes albopictus*’. Two Foshan, five FPA and two C6/36 contigs were identified to contain the PHB2 gene. The Rimini PHB2 sequence was assembled from a number of contigs whereas the Rimini_2 sequence is incomplete and is represented by two fragments containing sequences from 131-274 and 602-792 bp. **(a)** The contig sequences were aligned using Clustal Omega Multiple sequence alignment (https://www.ebi.ac.uk/Tools/msa/clustalo/). The Foshan_KQ562192.1 locus PHB2 gene sequence was included in the alignment. Results are depicted using MView. Conserved sequences are coloured. **(b)** Neighbour joining phylogenetic trees generated from the alignment (excluding the two incomplete Rimini contigs) in Clustal Omega. Because of the extended 3’ end of contig KQ562192.1, a second tree was built excluding the KQ562192.1 contig sequence. **(c)** The nucleotide sequences were translated into amino acid sequences using Expasy translate (<https://web.expasy.org/translate>/). The amino acid sequences were aligned using Clustal Omega Multiple sequence alignment (https://www.ebi.ac.uk/Tools/msa/clustalo/). The Foshan_KQ562192.1 locus PHB2 sequence was included in the alignment. Results are depicted using MView. Conserved sequences are coloured. Asterisks indicate amino acid variants.
