# Supplementary material for: CRISPR-editing of the virus vector Aedes albopictus cell line C6/36, illustrated by prohibitin 2 gene knockout
Source: MethodsX. 2024 Jun 21;13:102817. doi: 10.1016/j.mex.2024.102817 (PMC11267050; doi:10.1016/j.mex.2024.102817)
Supplement: Supplementary file 3 — Supplementary Figure S3 Flanking sequences used to design primers for nested PCR of the prohibitin 2 exon 1 fragment [file mmc3.docx]

**OF**

aa-54605_mRNA ------------------------------------------------------------ 0

aa-54604_mRNA ------------------------------------------------------------ 0

KQ571446.1_Foshan acgtgcagttgttggtgtacgcgttccaaagtaggtgtaaaaaagacctgaattctacaa 190

KQ562192.1_Foshan acgtgctgttgttggtgtacgcgttccaaagtaggtgtaaaaaatacctgaattctacaa 159

MNAF02001030.1_C6/36 acgtgctgttgttggtgtacgcgttccaaagtaggtgtaaaaaatacctgaattctacaa 469

MNAF02000396.1_C6/36 acgtgctgttgttggtgtacgcgttccaaagtaggtgtaaaaaatacctgaattctacaa 415

**C6/36_lab_cell_line** -----------ttggtgtacgcgttccaaagtaggtgtaaaaaatacctgaattctacaa 17

**IF**

aa-54605_mRNA ------------------------------------------------------------ 0

aa-54604_mRNA ------------------------------------------------------------ 0

KQ571446.1_Foshan aatttcatcacatattccggcgcggtgattgtgcaactctatccattggttctttccagc 250

KQ562192.1_Foshan aatttcatcatatattccggcgcggtgattgtgcaac--tatccattggttcttttcagc 217

MNAF02001030.1_C6/36 aatttcatcatatattccggcgcggtgattgtgcaac--tatccattggttctttccagc 527

MNAF02000396.1_C6/36 aatttcatcatatattccggcgcggtgattgtgcaac--tatccattggttctttccagc 473

**C6/36_lab_cell_line** aatttcatcatatattccggcgcggtgattgtgcaac--tatccattggttctttccagc 75

**5’ UTR**

aa-54605_mRNA ------------------------------------------------------------ 0

aa-54604_mRNA ------------------------------------------------------------ 0

KQ571446.1_Foshan tctaaaaactccatttgaaaagcgtagtttttacgcattgtctgcgtcggcggttccaaa 310

KQ562192.1_Foshan tctaataactccatttgagaagcgtacttattacgcagtgtctgcgacggcggttccaaa 277

MNAF02001030.1_C6/36 tctaaaagctccattcgagaagcgtactcattacgcagtgtctgcgacagcggttccaaa 587

MNAF02000396.1_C6/36 tctaaaagctccattcgagaagcgtacttattacgcagtgtctgcgacggcggttccaaa 533

**C6/36_lab_cell_line** tctaaaagctccattcgagaagcgtacttattacgcagtgtctgcgacggcggttccaaa 135

**start codon** **exon1**

aa-54605_mRNA **atg**gctcagagcaaactgaacgatttggccggaaaattcggtaagggtggaccacccgga 60

aa-54604_mRNA **atg**gctcagagcaaactgaacgatttggccggaaaattcggcaagggtggaccacccgga 60

KQ571446.1_Foshan **atg**gctcagagcaaactgaacgatttggccggaaaattcggtaagggtggaccacccgga 370

KQ562192.1_Foshan **atg**gctcagagcaaactgaacgatttggccggaaaattcggcaaaggtggtccacccgga 337

MNAF02001030.1_C6/36 **atg**gctcagagcaaactgaacgatttggccggaaaattcggcaagggtggtccacccgga 647

MNAF02000396.1_C6/36 **atg**gctcagagcaaactgaacgatttggccggaaaattcggcaagggtggaccacccggt 593

**C6/36_lab_cell_line** **atg**gctcagagcaaactgaacgatttggccggaaaattcggcaagggtggaccacccggt 195

**C6/36_lab_cell_line SNP** **t a**

***************************************** ** ***** ********

**target** **PAM**

aa-54605_mRNA ttggcgaccggcctgaagctgctggca**gctgtcggtgccgctgcctacgg**aatcaacaat 120

aa-54604_mRNA ttggcgaccggcctgaagctgctggca**gctgtcggtgccgctgcctacgg**aattaacaat 120

KQ571446.1_Foshan ttggcgaccggcctgaagctgctggca**gctgtcggtgccgctgcctacgg**aatcaacaat 430

KQ562192.1_Foshan ttggcgaccggcctgaagctgctggca**gctgtcggtgccgctgcctacgg**aattaacaat 397

MNAF02001030.1_C6/36 ttggcgaccggcttgaagctgctggca**gctgtcggtgccgctgcctacgg**aattaacaat 707

MNAF02000396.1_C6/36 ttggcgaccggcttgaagctgctggca**gctgtcggtgccgctgcctacgg**tatcaacaat 653

**C6/36_lab_cell_line** ttggcgaccggcttgaagctgctggca**gctgtcggtgccgctgcctacgg**tatcaacaat 255

**C6/36_lab_cell_line SNP** **a t**

************ ************************************* ** ******

**intron**

aa-54605_mRNA tccatgttcacagtcgaaggtggccaccgtgcaatcatgtttaa-----ccgaattggcg 175

aa-54604_mRNA tctatgttcacagttgagggtggccaccgtgcaatcatgttcaa-----ccgaattggcg 175

KQ571446.1_Foshan tccatgttcacaggtaagtttgggttttggttctgtgagggattttaagcagttttgaag 490

KQ562192.1_Foshan tccatgttcacaggtgagtttggactgtggttctgtgggggattttgagcagttttgaag 457

MNAF02001030.1_C6/36 tccatgttcacaggtgagtttgggctgcggttctgtgggggattttgagcagttttgaag 767

MNAF02000396.1_C6/36 tccatgttcacaggtgagtttggaatgtggttctgtgggggattttgagcagttttgaag 713

**C6/36_lab_cell_line** tccatgttcacaggtgagtttggaatgtggttctgtgggggattttgagcagttttgaag 315

** ********** * *** * * * * *** *

**IR**

aa-54605_mRNA gagtcggtgatgacatcttcagtgaaggactacacttccgagttccgtggttccagtacc 235

aa-54604_mRNA gagtcggcgatgacatcttcagtgaagggctgcacttccgagttccgtggttccagtacc 235

KQ571446.1_Foshan tcttgaatgtagagaacataaccttca-aataccgctggaacttgattggggctgatttc 549

KQ562192.1_Foshan tcttggatgcagggaacataaccttca-aataccgctggaacttgattggggctgatttc 516

MNAF02001030.1_C6/36 tcttgggtgcagggaacataaccttca-aataccgctggaacttgattggggctgatttc 826

MNAF02000396.1_C6/36 ttttgggtgcagggaacataaccttca-aataccgctggaacttgattggggctggtctc 772

**C6/36_lab_cell_line** ttttgggtgcagggaacataaccttca-aataccgctggaacttgattggggctggtctc 374

* * * * * * * * * * * ** *** * * *

**OR**

aa-54605_mRNA cgattgtgtacgacatccgttcccgcccgaggaaaatctcgtccccaactggttcgaagg 295

aa-54604_mRNA cgattgtgtacgatatccgttcccgccccaggaaaatctcgtccccaactggttccaagg 295

KQ571446.1_Foshan acatggagattacacaat-ataatacggattgacataatccgggaggatactctctcttt 604

KQ562192.1_Foshan acatggagattacacaatgataatacggattgacataatccgggaggatactctctcttt 572

MNAF02001030.1_C6/36 acatggagattacacaat-ataatacggattgaaataatccgggaggatactctctcttt 881

MNAF02000396.1_C6/36 acatggagattacacaatgataatacggattgacataatccgggaggatacactctcttt 828

**C6/36_lab_cell_line** acatggagattacacaatgataatacggattgacataatccggga 430

** * * * * ** * ** *

**Supplementary Figure S3 Flanking sequences used to design primers for nested PCR of the prohibitin 2 exon 1 fragment**

Alignment of the *Aedes albopictus* prohibitin 2 (PHB2) exon 1 flanking sequences (5’ untranslated region (5’ UTR) & intron) from the Foshan (KQ571446.1, KQ562192.1) and C6/36 (MNAF02000396.1, MNAF02001030.1) whole genome shotgun sequences and the transcriptome shotgun assembly (Aa-54605, Aa-54604) was used to design primers for nested PCR. The exon 1 sequence is highlighted in green with the start codon highlighted in dark blue. The inner forward (IF) and reverse (IR) primers are highlighted in yellow. The outer forward (OF) and reverse (OR) primers are highlighted in sky blue. The primers were used in nested PCR to amplify up the PHB2 exon 1 fragment from our laboratory-passaged C6/36 cell line. The sequence of our laboratory-passaged C6/36 PHB2 exon 1, single nucleotide polymorphism (SNP) and flanking sequences are added to the alignment retrospectively. It also highlights the target sequence subsequently chosen as the CRISPR RNA (crRNA) in red and the protospacer adjacent motif (PAM) in violet.
