# Supplementary material for: CRISPR-editing of the virus vector Aedes albopictus cell line C6/36, illustrated by prohibitin 2 gene knockout
Source: MethodsX. 2024 Jun 21;13:102817. doi: 10.1016/j.mex.2024.102817 (PMC11267050; doi:10.1016/j.mex.2024.102817)
Supplement: Supplementary file 4 — Supplementary Table S1a Targeta and predicted off-targetb sequences of GCTGTCGGTGCCGCTGCCTA in the C6/36 cell line genomec Supplementary Table S1b Targeta and predicted off-targetb sequences of GGCGACCGGCTTGAAGCTGC in the C6/36 cell line genomec Supplementary Table S1c Primer pair for the detection of potential off-target MNAF02000045.1a [file mmc4.docx]

**Supplementary Table S1a Target^a^ and predicted off-target^b^ sequences of GCTGTCGGTGCCGCTGCCTA in the C6/36 cell line genome^c^**

| **Target** | **Chromosome** | **Position** | **Direction** | **Mismatches** |
| --- | --- | --- | --- | --- |
| **crRNA: GCTGTCGGTGCCGCTGCCTANGG   DNA: GCTGcCGcTGCtGCTGCCTACGG** | MNAF02000045.1 | 532831 | + | 3 |
| **crRNA: GCTGTCGGTGCCGCTGCCTANGG   DNA: GCTGTtGGTGCtGCTGCCcATGG** | MNAF02000106.1 | 7314550 | + | 3 |
| **crRNA: GCTGTCGGTGCCGCTGCCTANGG   DNA: GCTGTtGGTGCCGCTGaCTgTGG** | MNAF02000224.1 | 2758372 | + | 3 |
| **crRNA: GCTGTCGGTGCCGCTGCCTANGG   DNA: GCTGTCGGTGCCGCTGCCTACGG** | MNAF02000396.1 | 1479419 | + | 0 |
| **crRNA: GCTGTCGGTGCCGCTGCCTANGG   DNA: GCTGTCGGTGCCGCTGCCTACGG** | MNAF02001030.1 | 1251003 | - | 0 |
| **crRNA: GCTGTCGGTGCCGCTGCCTANGG   DNA: GCTGTtGGTGCtGCTGCCcATGG** | MNAF02001226.1 | 2686686 | - | 3 |
| **crRNA: GCTGTCGGTGCCGCTGCCTANGG   DNA: GCTGaCGGTGCCGCTGCtTcCGG** | MNAF02001287.1 | 3814110 | - | 3 |

**^a^** target sequence is denoted by 0 mismatch with the genomic sequence

**^b^** off-target sequence is denoted by 3 mismatches with the genomic sequence and mismatch is highlighted in yellow lowercase (there are no 1 or 2 mismatches)

**^c^** prediction was performed using Cas-OFFinder against whole genome shotgun sequence of the C6/36 cell line (accession no. MNAF02000000). Bulge type and bulge size were not included in the prediction

**Supplementary Table S1b Target^a^ and predicted off-target^b^ sequences of GGCGACCGGCTTGAAGCTGC in the C6/36 cell line genome^c^**

| **Target** | **Chromosome** | **Position** | **Direction** | **Mismatches** |
| --- | --- | --- | --- | --- |
| **crRNA: GGCGACCGGCTTGAAGCTGCNGG   DNA: GGCGACCGGCTTGAAGCTGCTGG** | MNAF02000396.1 | 1479394 | + | 0 |
| **crRNA: GGCGACCGGCTTGAAGCTGCNGG   DNA: tGCGACCatCTTGAAGCTGCGGG** | MNAF02000460.1 | 115744 | - | 3 |
| **crRNA: GGCGACCGGCTTGAAGCTGCNGG   DNA: GGCGACCGGCTTGAAGCTGCTGG** | MNAF02001030.1 | 1251028 | - | 0 |
| **crRNA: GGCGACCGGCTTGAAGCTGCNGG   DNA: tGCGACCatCTTGAAGCTGCGGG** | MNAF02001485.1 | 636508 | - | 3 |
| **crRNA: GGCGACCGGCTTGAAGCTGCNGG   DNA: GGCGACCaGCTgGAAGCTGaCGG** | MNAF02001591.1 | 1660665 | - | 3 |
| **crRNA: GGCGACCGGCTTGAAGCTGCNGG   DNA: GGaGACCGGCTTGAcGCgGCAGG** | MNAF02001968.1 | 1367644 | + | 3 |

**^a^** target sequence is denoted by 0 mismatch with the genomic sequence

**^b^** off-target sequence is denoted by 3 mismatches with the genomic sequence and mismatch is highlighted in yellow lowercase (there are no 1 or 2 mismatches)

**^c^** prediction was performed using Cas-OFFinder against whole genome shotgun sequence of the C6/36 cell line (accession no. MNAF02000000). Bulge type and bulge size were not included in the prediction

**Supplementary Table S1c Primer pair for the detection of potential off-target MNAF02000045.1^a^**

**
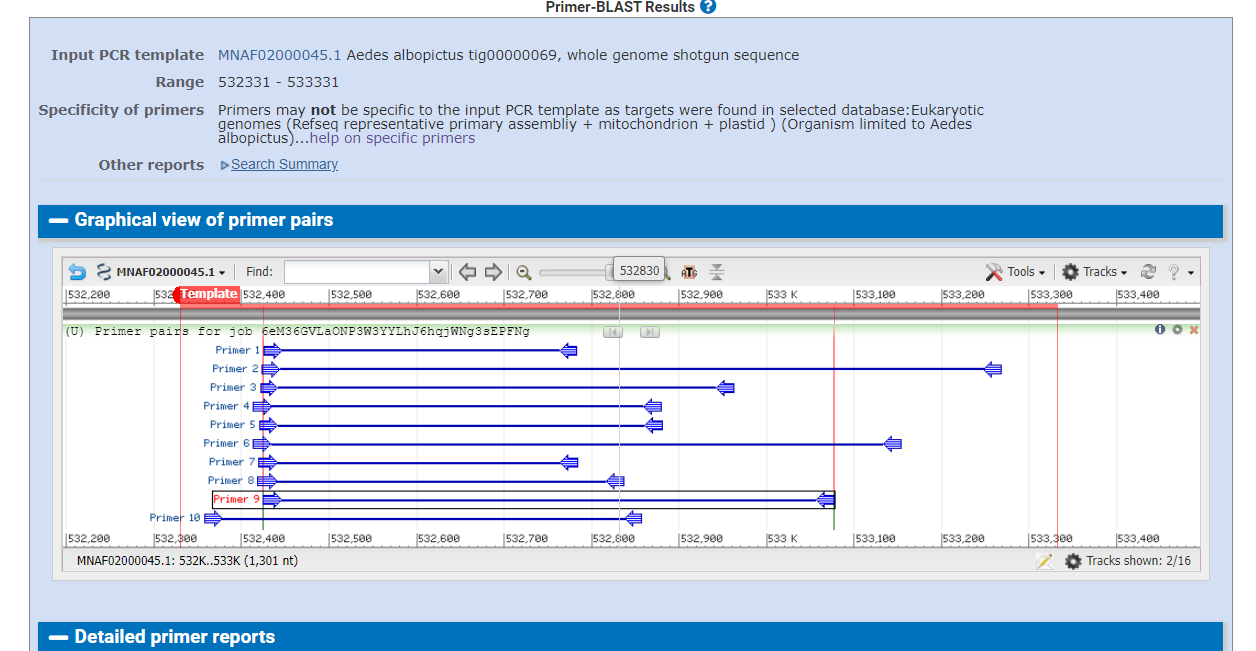
**

**Primer pair 9**

|  | **Sequence (5'->3')** | **Template strand** | **Length** | **Start** | **Stop** | **Tm** | **GC%** | **Self complementarity** | **Self 3' complementarity** |
| --- | --- | --- | --- | --- | --- | --- | --- | --- | --- |
| **Forward primer** | CAGCTAAACAAACTGCGCTGC | Plus | 21 | 532425 | 532445 | 61.25 | 52.38 | 4.00 | 3.00 |
| **Reverse primer** | ATCAGTTGATGGCTGGCTGT | Minus | 20 | 533076 | 533057 | 59.67 | 50.00 | 6.00 | 0.00 |
| **Product length** | 652 | | | | | | | | |

**Primer pair 6**

|  | **Sequence (5'->3')** | **Template strand** | **Length** | **Start** | **Stop** | **Tm** | **GC%** | **Self complementarity** | **Self 3' complementarity** |
| --- | --- | --- | --- | --- | --- | --- | --- | --- | --- |
| **Forward primer** | CGACAGAGAGCCAGCTAAAC | Plus | 20 | 532414 | 532433 | 58.37 | 55.00 | 4.00 | 1.00 |
| **Reverse primer** | AGTACCCGCATTCATCACCG | Minus | 20 | 533153 | 533134 | 60.18 | 55.00 | 4.00 | 2.00 |
| **Product length** | 740 | | | | | | | | |

**^a^** The accession number MNAF02000045.1 was used as an input PCR template in Primer-BLAST and the primer range was set at 500 bases either side of the off-target position 532831 (start: 532331; stop: 533331). The graphic view displays the primer pairs. Primer pair 9 produces a 652bp PCR product which covers the off-target position. Primer pair 6 can also be used.
