# Supplementary material for: CRISPR-editing of the virus vector Aedes albopictus cell line C6/36, illustrated by prohibitin 2 gene knockout
Source: MethodsX. 2024 Jun 21;13:102817. doi: 10.1016/j.mex.2024.102817 (PMC11267050; doi:10.1016/j.mex.2024.102817)
Supplement: Supplementary file 5 — Supplementary Table S2 Target sequences in exon 1 of the Aedes albopictus prohibitin 2 gene and off-target predictions [file mmc5.docx]

**Supplementary Table S2 Target sequences in exon 1 of the *Aedes albopictus* prohibitin 2 gene and off-target predictions**

| **sense strand targets^a^**  5’ATGGCTCAGAGCAAACTGAACGATTTGGCCGGAAAATTCGG(t/c)AA(g/a)GGTGG(a/t)CCACCCGGATTGGCGACCGGCCTGAAGCTGCTGGCAGCTGTCGGTGCCGCTGCCTACGGAAT(c/t)AACAATTCCATGTTCACAG-exon 1-*G*-3’ | | | | | | |
| --- | --- | --- | --- | --- | --- | --- |
|  | mismatches**^b^** | | | |  |  |
| target-NGG (PAM) (5’-3’) | 0 | 1 | 2 | 3 | out-of-frame score**^c^** | %GC**^d^** |
| TCAGAGCAAACTGAACGATT | 2 | 0 | 0 | 3 | 54.1 | 40 |
| AGCAAACTGAACGATTTGGC | 2 | 0 | 0 | 1 | 63 | 45 |
| AACGATTTGGCCGGAAAATT | 2 | 0 | 0 | 10 | 51.4 | 40 |
| TTTGGCCGGAAAATTCGGTA  -------------------- | 1 | 0 | 0 | 1 | 53 | 45 |
| TTGGCCGGAAAATTCGG**t**AA  TTGGCCGGAAAATTCGG**c**AA | 1  1 | 1  1 | 0  0 | 6  6 | 49.8  48.2 | 45  50 |
| GCCGGAAAATTCGG**t**AA**g**GG  GCCGGAAAATTCGG**c**AA**a**GG | 1  1 | 0  0 | 1  1 | 2  6 | 48.1  47.5 | 55  55 |
| TTCGG**t**AA**g**GGTGG**a**CCACC  TTCGG**c**AA**a**GGTGG**t**CCACC | 1  1 | 0  0 | 0  0 | 4  7 | 51.6  52.1 | 60  60 |
| **t**AA**g**GGTGG**a**CCACCCGGAT  **c**AA**a**GGTGG**t**CCACCCGGAT | 1  1 | 0  0 | 2  0 | 5  2 | 64.9  70.2 | 60  60 |
| GG**a**CCACCCGGATTGGCGAC  GG**t**CCACCCGGATTGGCGAC | 1  1 | 1  1 | 0  0 | 2  3 | 61.9  61.3 | 70  70 |
| GGCGACCGGCCTGAAGCTGC | 2 | 0 | 0 | 1 | 60.5 | 75 |
| CTGAAGCTGCTGGCAGCTGT | 2 | 0 | 1 | 6 | 65.7/65.3 | 60 |
| **GCTGTCGGTGCCGCTGCCTA** | 2 | 0 | 0 | 7 | 57/58.3 | 70 |
| AT**c**AACAATTCCATGTTCAC  AT**t**AACAATTCCATGTTCAC | 1  1 | 1  1 | 0  0 | 1  1 | 65.8  66.1 | 35  30 |
| **antisense strand targets^a^**  3’TACCGAGTCTCGTTTGACTTGCTAAACCGGCCTTTTAAGCC(a/g)TT(c/t)CCACC(t/a)GGTGGGCCTAACCGCTGGCCGGACTTCGACGACCGTCGACAGCCACGGCGACGGATGCCTTA(g/a)TTGTTAAGGTACAAGTGTC-*C*-5’ | | | | | | |
|  | mismatches**^b^** | | | |  |  |
| target-NGG (PAM) (5’-3’) | 0 | 1 | 2 | 3 | out-of-frame score**^c^** | %GC**^d^** |
| CATGGAATTGTT**g**ATTCCGT  CATGGAATTGTT**a**ATTCCGT | 1  1 | 1  1 | 1  2 | 6  2 | 49.3  54.2 | 40  35 |
| ATTGTT**g**ATTCCGTAGGCAG  ATTGTT**a**ATTCCGTAGGCAG | 1  1 | 1  1 | 0  0 | 2  0 | 50.8  51.5 | 45  40 |
| CGACAGCTGCCAGCAGCTTC | 2 | 0 | 0 | 4 | 58.6/59 | 65 |
| AGCTGCCAGCAGCTTCAGGC | 2 | 0 | 0 | 8 | 61.2/62.1 | 65 |
| CTTCAGGCCGGTCGCCAATC | 2 | 0 | 0 | 3 | 73.5/76.2 | 65 |
| TTCAGGCCGGTCGCCAATCC | 2 | 0 | 0 | 1 | 72.9/75.4 | 65 |
| AGGCCGGTCGCCAATCCGGG | 2 | 0 | 0 | 0 | 57.9/59.1 | 75 |
| **t**CCACC**c**TT**a**CCGAATTTTC  **a**CCACC**t**TT**g**CCGAATTTTC | 1  1 | 0  0 | 1  0 | 5  8 | 55.8  57.7 | 45  45 |

**^a^** The target sequences were read from the 5’-3’ direction for both sense and anti-sense strands. NGG protospacer adjacent motifs (PAMs) are highlighted in pink. Overlapping PAMs are underlined. NGG underlined by a dash line indicates the presence of PAM in one allele only due to single nucleotide polymorphism (SNP). The extra G (in sense strand that forms part of the PAM) and C (in anti-sense strand) in the intronic region are in blue italics. Targets were manually identified and confirmed with Cas-Designer. The sequence equivalent to the selected target sequence in the C6/36 cell line is in red. Target sequence with four ‘T’ can cause termination of RNA polymerase III and is greyed out. Lowercase letters indicate SNP (Fig.1). Sequences highlighted in beige are the seven entries returned by CRISPR GuideXpress.

**^b^** indicates the number of mismatches with the target sequence as predicted by Cas-OFFinder. Target sequences with two 0 mismatches, without 1 and 2 mismatches and ≤5 three mismatches are highlighted in yellow.

**^c^** indicates the probability of generating out-of-frame insertions and deletions in CRISPR-edited site as predicted by Cas-Designer. Since the out-of-frame scores are calculated in the context of neighbouring gene sequences, there are two out-of-frame scores (separated by a slash) for some identical sequences due to the presence of neighbouring SNP in the allelic genes. A score of >66 is recommended and those with a score >66 are highlighted in yellow.

**^d^** indicates %GC as predicted by Cas-Designer and the recommended range is 20-80%
