# Supplementary material for: CRISPR-editing of the virus vector Aedes albopictus cell line C6/36, illustrated by prohibitin 2 gene knockout
Source: MethodsX. 2024 Jun 21;13:102817. doi: 10.1016/j.mex.2024.102817 (PMC11267050; doi:10.1016/j.mex.2024.102817)
Supplement: Supplementary file 6 — Supplementary S1_File (detailed step-by-step protocols) [file mmc6.pdf]

PROTOCOLS

CRISPR-editing of the virus vector *Aedes albopictus* cell line, C6/36, illustrated by prohibitin 2 gene knockout

Shiu-Wan Chan[1*]

[1]Faculty of Biology, Medicine and Health, School of Biological Sciences, The University of Manchester, Michael Smith Building, Oxford Road, Manchester M13 9PT, United Kingdom

*** Correspondence:**
Corresponding Author
shiu-wan.chan@manchester.ac.uk

ABSTRACT

Aedes mosquitoes are important vectors for human viruses. Comprehensive methods, reagents and web tools available for CRISPR-editing of commonly studied organisms are lacking for functional study in mosquito cell lines pivotal in understanding vector biology, vector competence and host-pathogen interactions and in gene annotations. Here, we described methodologies of CRISPR-Cas9-editing in the *Ae. albopictus* mosquito cell line, C6/36, and showed that we could use CRISPR to knock down/out the prohibitin 2 (PHB2) gene previously shown to be refractory to siRNA silencing, providing a valuable toolkit for editing difficult-to-knockdown gene in mosquito cells.

1. Database search for the *Aedes albopictus* PHB2 gene sequence

1.1 Search for mosquito whole genome shotgun (WGS) sequences

- Go to National Centre for Biotechnology Information (NCBI) GenBank (https://www.ncbi.nlm.nih.gov)
- Select 'genome' in the dropdown menu
- Type in search box 'Aedes albopictus'
- It should return four WGS sequences (one Foshan laboratory isolate derived from wild mosquitoes in the Southeastern China: JXUM00000000.1; one Foshan inbred line FPA with two assemblies: SWKZ00000000.1 & JAFDOQ000000000.1; one Rimini isolate: LMAV000000000.1 and one C6/36 cell line derived from the larval tissue of *Ae. albopictus*: MNAF00000000.2

1.2 Search for mosquito PHB2 gene

1.2.1 from the Foshan WGS sequence loci

- Go to National Centre for Biotechnology Information (NCBI) GenBank (https://www.ncbi.nlm.nih.gov)
- Select 'nucleotide' in the dropdown menu
- Type in search box 'Aedes albopictus PHB2'
- It should return two loci KQ562192.1 and KQ571446.1 from the Foshan strain WGS sequence that encodes a hypothetical protein homologous to the mammalian PHB2 protein. Scroll down to the PHB2 gene. The amino acid sequence is displayed and can be retrieved by cut-and-paste into word or notepad
- Retrieve the transcript sequence by clicking on the 'mRNA' feature and click on the 'FASTA' button

1.2.2 from the FPA WGS sequence loci

- Go to National Centre for Biotechnology Information (NCBI) GenBank (https://www.ncbi.nlm.nih.gov)
- Select 'nucleotide' in the dropdown menu
- Type in search box 'Aedes albopictus prohibitin 2' or 'Aedes albopictus prohibitin'
- It should return two loci NW_021838943.1 and NW_021837434.1 from the FPA isolate WGS sequence that encodes a hypothetical protein homologous to the mammalian PHB2 protein. Sequences were not retrieved. Sequences were retrieved as in 1.2.3 below.

1.2.3 PHB2 transcript variants

- Go to National Centre for Biotechnology Information (NCBI) GenBank (https://www.ncbi.nlm.nih.gov)
- Select 'nucleotide' in the dropdown menu
- Type in search box 'Aedes albopictus prohibitin-2'
- It should return two sets of transcripts. The first set of transcripts is derived from the transcriptome shotgun assembly (TSA) loci GAPW01002914.1 and GAPW01002921.1 that contains putative PHB2 mRNAs, Aa-54605 and Aa-54604, respectively. The second set of transcripts contains four variants (X1 to X4) from the FPA genomic scaffold NW_021838943.1. Click on the description to retrieve the nucleotide and amino acid sequences.

1.2.4 BLAST search of PHB2 from the C6/36 and Rimini WGS sequence genomes

- Go to BLAST https://blast.ncbi.nlm.nih.gov
- Click on ‘nucleotide BLAST’ button
- Enter the retrieved sequence of the PHB2 mRNA from the KQ571446.1 contig as described above in 1.2.1 into the query sequence box.
- Select ‘standard’ databases and ‘Whole genome shotgun contigs (wgs)’ in the dropdown menu. Set limit by ‘organism’ to ‘Aedes albopictus (Skuse, 1894) (taxid:7160)’. Optimize for ‘highly similar sequences (Megablast)’. A list of the results will be displayed in the blastn suite
- You should see all the contigs from Foshan, FPA, C6/36 and Rimini WGS genomes. Click on each contig description to see alignment of gene segments with the input sequence to locate the positions of the PHB2 coding segments in the contig. Click on the sequence ID above the alignment and assemble the segments into complete PHB2 coding sequence using ‘Change Region Shown’ and then ‘select region’ buttons, using the positions to delineate the gene segments
- Translate the assembled nucleotide sequence into amino acid sequence using Expasy translate as described in 2.4

2. Alignment of human and insect PHB2

2.1 Identification of human PHB2 reference sequence

- Go to National Centre for Biotechnology Information (NCBI) GenBank (https://www.ncbi.nlm.nih.gov)
- Select ‘gene’ in the dropdown menu
- Type in search box ‘PHB2’
- It should return the human PHB2 Refseq transcripts and Refseq proteins. Click on Refseq proteins to reveal the three human isoforms, 1, 3 and X1. Obtain the accession no. and retrieve the sequences

2.2 Identification of Drosophila PHB2 reference sequence

- As above from NCBI. Instead of clicking on the Refseq proteins, scroll down the list to find Drosophila melanogaster. Click on Phb2. It should return a full report. Click on ‘Transcript Table’ and it should return the six transcript variants PA-PF and you can find the accession no. and retrieve the sequences from there

OR from FlyBase

- The NCBI search from above will give you a full report in which you can find the link to the FlyBase gene FLYBASE:Fbgn0010551 (http://flybase.org/reports/FBgn0010551)
- Alternatively, go direct to FlyBase (https://flybase.org)
- In the J2G (Jump to Gene) menu, type in search box ‘PHB2’. It will give you the page on Gene: Dmel/Phb2
- Scroll down to Gene Model and Products. Open it. You should see the transcript variants Phb2-PA-PF
- Right click on each transcript and click on view details to retrieve the sequence

2.3 Reverse complement of gene on the minus strand

- Some genes are on the minus strand and will need to be converted into the plus strand sequence
- Go to Reverse Complement https://www.bioinformatics/org/sms/rev_comp.html

- Paste the sequence into the box
- Select 'reverse-complement'
- Click submit. The reverse complement will be displayed

2.4 Translation of nucleotides into amino acid sequences

- Open Expasy translate (https://web.expasy.org/translate/)
- Enter individual nucleotide sequence into the box
- Select output format to Compact: M, -, no spaces
- Set DNA strands in forward and/or reverse orientation
- Use standard genetic code
- Click the 'TRANSLATE' button
- Predicted amino acids in the chosen reading frames will be displayed. Choose the correct amino acid sequence and cut and paste into word or notepad

2.5 CLUSTAL Omega Multiple sequence alignment

- Open CLUSTAL Omega https://www.ebi.ac.uk/tools/msa/clustalo/
- Enter DNA or amino acid sequences in FASTA format i.e.
  <name_of_sequence
  sequence
- Output format can be set at 'aligned' or 'input'
- In the results page, sequence alignment is displayed and can be cut and paste into words or notepad. To change results display, click on 'RESULTS VIEWERS' button. Select View in MView. Set output parameters
- To view the phylogenetic trees, select the 'Phylogenetic Tree' button in the results page

3. Sequencing of C6/36 PHB2 exon 1 sequence

3.1 Cultivation of C6/36 cells

Materials:

- C6/36 cells (ATCC CRL-1660™)
- C6/36 growth medium: Eagle's minimal essential medium (MEM) #M4655 (Sigma-Aldrich) supplemented with 1x non-essential amino acid (NEAA) #M7145 (Sigma-Aldrich), 10% fetal calf serum (FCS) #16500-064 (Gibco) and 100 U/ml penicillin, and 100mg/ml streptomycin #P0781 (Sigma-Aldrich)
- Tissue culture flasks, dishes and plates (Corning)
- CO_2 incubator at 28°C and 5% CO_2 (MiniGalaxy E)

Methods:

- Grow C6/36 in growth medium in a CO_2 incubator at 28°C and 5% CO_2

3.2 Genomic DNA extraction

Materials:

- PureLink® Genomic DNA Kits #K1820-01(Invitrogen)
- Make up PureLink® Genomic Wash Buffer 1 and PureLink® Genomic Wash Buffer 2 by adding 96–100% ethanol according to instructions on each label. Mix well.
- trypsin #T3924 (Sigma-Aldrich)
- DPBS #D8537 (Sigma-Aldrich)

- PBS #P4417-100TAB (Sigma-Aldrich) made up one tablet in 200ml milli-Q water; autoclaved
- ethanol #E/0650DF/P17 (Fisher)
- agarose gel (section 3.4)
- autoclaved milli-Q water
- water bath (Grant) or heat block (Grant) at 55°C
- microfuge MicroCentaur Plus (MSE)
- refrigerated centrifuge (Sorvall RT6000B)
- NanoDrop ND-1000 (section 3.3)

Methods:

Extract genomic DNA from the C6/36 cells using PureLink® Genomic DNA Kits (Invitrogen) according to the manufacturer's instructions.

- Trypsinize <5x10^6 C6/36 cells. Pellet cells by centrifugation at 1000rpm for 5min, wash cells once in DPBS. Pellet cells by centrifugation at 1000rpm for 5min. Re-suspend pellet in 200µl PBS
- Add 20µl Proteinase K (20mg/ml supplied with the kit) and 20µl RNase A (20mg/ml supplied with the kit) to the sample. Incubate at RT for 2 min
- Add 200µl of PureLink® Genomic Lysis/Binding Buffer to lyse cells. Mix by vortexing until sample is homogenous
- Incubate at 55°C for 10min
- Add 200µl of 96-100% ethanol to the lysate. Mix by vortexing for 5s
- Add the lysate to a PureLink® Spin Column in a collection Tube. Spin at 10,000g/1min/RT
- Discard the flow-through. Add 500µl Wash Buffer 1 to the column. Spin at 10,000g/1min/RT
- Discard the flow-through. Add 500µl Wash Buffer 2 to the column. Spin at 14,000rpm/3min/RT
- Discard the flow-through. Spin at 14,000rpm/1.5min/RT to remove any residual liquid
- Discard collection tube. Place the spin column in a sterile 1.5ml Eppendorf tube
- Add 50µl autoclaved milli-Q water to the column to cover the membrane for 1min
- Spin at 14,000rpm/1min/RT to collect the eluted DNA in the Eppendorf tube
- Keep DNA on ice
- Measure the DNA concentration using NanoDrop (see 3.3)
- Check the quality of the DNA by running 1µl on agarose gel electrophoresis (see 3.4)
- Store the DNA at -20°C.

3.3 Measuring DNA concentration using NanoDrop

Materials:

- autoclaved milli-Q water
- pipette for 2µl (Gilson or your preferred brand)
- soft laboratory paper
- NanoDrop ND-1000 (Thermo Scientific)

Methods:

- Program the NanoDrop for DNA measurement. Select 'Nucleic Acid' at the main menu. Select sample type 'DNA-50' at the dropdown menu.

- Pipette 2µl of diluent (autoclaved milli-Q water for DNA) onto the lower measurement pedestal. Close the sampling arm. Press OK to initialize machine.
- Wipe clean both pedestals gently with soft laboratory wipe.
- Pipette 2µl of diluent (autoclaved milli-Q water for DNA) onto the lower measurement pedestal. Close the sampling arm. Press ‘BLANK’.
- Wipe clean both pedestals gently with soft laboratory wipe.
- Pipette 2µl of DNA sample onto the lower measurement pedestal. Close the sampling arm. Press ‘MEASURE’
- DNA concentration will be displayed as ng/µl. DNA purity is indicated by $260/280 \geq 1.8$ and $260/230 \geq 1.8$-2.2
- Wipe clean both pedestals gently with soft laboratory wipe
- Wash the NanoDrop by pipetting 2µl of diluent (autoclaved milli-Q water for DNA) onto the lower measurement pedestal. Close the sampling arm. Press ‘MEASURE’
- Wipe clean both pedestals gently with soft laboratory wipe

3.4 Agarose gel electrophoresis

Materials:

- agarose molecular grade #A9539 (Sigma-Aldrich)
- Tris #BP152-1 (Fisher)
- glacial acetic acid #A/0400/PB15 (Fisher)
- EDTA #E5134 (Sigma-Aldrich)
- TAE (0.4M Tris-acetate, 0.01M EDTA, pH8.3)
- ethidium bromide 10mg/ml #E1510 (Sigma-Aldrich)
    - Note: Ethidium bromide is a known mutagen. Follow all the health and safety measures when handling ethidium bromide. Safer alternatives to ethidium bromide include SafeView #NBS-SV1 (NBS Biologicals) etc.
- molecular weight marker (commercially available 1kb ladder, 100bp ladder from Fermentas, Bioline, New England Biolabs etc)
- loading buffer usually supplied with molecular weight marker
- 250ml conical flask (PYREX)
- gel tray (Bio-Rad)
- gel tank (Bio-Rad)
- Powerpac 300 (Bio-Rad)
- microwave oven
- UV transilluminator Uvipro Silver (Uvitec)
- Printer

Methods:

- Melt agarose in TAE buffer in a 250ml flask to the desired percentage (1% for genomic DNA) in a microwave oven
- When cool, add 1µl of ethidium bromide to agarose. Mix by gentle swirling. Pour agarose onto gel tray
- Make up samples in loading buffer.
- When gel is set, load samples together with a molecular weight marker
- run samples at 100V for the desired time (1h for genomic DNA)
- Visualize bands with a UV transilluminator. Take picture. Save picture to computer.

3.5 Design primers for nested PCR

- Retrieve PHB2 nucleotide sequences from Foshan (KQ571446.1, KQ562192.1) and C6/36 WGS contigs (MNAF02000396.1, MNAF02001030.1) to include exon 1 plus ~200 bases 5' and 3' to exon 1
- Go to National Centre for Biotechnology Information (NCBI) GenBank (https://www.ncbi.nlm.nih.gov)
- Select 'nucleotide' in the dropdown menu. In search box type in KQ571446.1. The page on KQ571446.1 appears. Click on 'mRNA' features for PHB2 then 'FASTA' button and note down the first coding sequence positions for exon 1 above the sequence i.e. 10610-10478. If the positions run in ascending order the gene is on plus strand. If the positions run in descending order the gene is on minus strand. The gene is on minus strand of the contig KQ571446.1
- Add ~200 bases to either side of the coding sequence positions for exon 1 to set the search range. Add 200 bases to the bigger number 10610 and subtract 200 bases from the smaller number 10478 to give a new range 10810-10278
- Go back to the KQ571446.1 page. Click the 'FASTA' button. Go to 'Change Region Shown' on the right hand button. Input the search range into the begin and end positions of the selected region
- Click 'update view' to retrieve the sequence
- For minus strand sequence, convert into reverse-complement as in section 2.3
- Repeat with KQ562192.1. MNAF02000396.1 and MNAF02001030.1. For MNAF02000396.1 and MNAF02001030.1, the coding sequence positions for exon 1 have been located as in 1.2.4 above.
- Go to CLUSTAL Omega https://www.ebi.ac.uk/tools/msa/clustalo/
- Align the above retrieved sequences together with the transcript variants (Aa-54605, Aa-54604) from the TSA (1.2.3 above), which help to delineate the 5'UTR and exon-intron boundary (see section 2.5)
- Select 20-mer inner primers about 70 bases from the start codon or the exon-intron boundary and the outer primers about 60 bases from the inner primers in the conserved regions, if possible, keeping GC content within 40-60%
- Check the primer quality, Tm and specificity using Primer-BLAST https://www.ncbi.nlm.nhs.gov/tools/primer-blast/index.cgi. Use KQ571446.1 10810-10278 as the input query sequence and own forward and reverse primer pair. Select database for 'Genomes for selected eukaryotic organisms' and select 'Aedes albopictus (Skuse, 1894) (taxid:7160)'. Click 'GET PRIMERS' button.

3.6 Nested PCR to amplify PHB2 exon 1

Materials:

- Order the following primers from Eurofins Genomics (or your preferred source)

primer	sequence	final concentration
IF AeAlbop (IF)	5'-ATATTCCGGCGCGGTGATTG-3'	10μM
IR AeAlbop (IR)	5'-CCCCAATCAAGTTCCAGCGG-3'	10μM
OF AeAlbop (OF)	5'-TTGGTGTACGCGTTCCAAAG-3'	10μM
OR AeAlbop (OR)	5'-TCCCGGATTATGTCAATCCG-3'	10μM

- genomic DNA
- *Taq* DNA polymerase 5U/μl #M0267S (New England Biolabs)

- 10xThermoPol® buffer containing 200mM Tris-HCl, 100mM $(NH_4)_2SO_4$, 100mM KCl, 20mM $MgSO_4$, 1% Triton® X-100 pH 8.8@25°C #B9004 (supplied with *Taq* DNA polymerase)
- 10mM dNTP #1277049 (Roche)
- filtered milli-Q water: filter through 0.22mm filter E4780-1223 (STARLAB)
- PCR machine MULTIGENE™ MINI (Labnet)

Methods:

- Set up first PCR as below:

**reagent**	**volume**	**final concentration**
10xThermoPol® buffer	2μl	1x
10mM dNTP	0.4μl	200μM
IF (10μM)	0.4μl	200nM
IR (10μM)	0.4μl	200nM
genomic DNA	xμl (=1μg)	
Taq polymerase 5U/μl	0.1μl	0.025U/μl
filtered milli-Q water	make up to total volume of 20μl	

<table>
<tr><th>step</th><th>temperature</th><th>time</th><th>no. of cycle</th></tr>
<tr><td>initial denaturation</td><td>94°C</td><td>2min</td><td>1</td></tr>
<tr><td>denaturation</td><td>94°C</td><td>25s</td><td rowspan="3">30</td></tr>
<tr><td>annealing</td><td>57°C</td><td>35s</td></tr>
<tr><td>extension</td><td>68°C</td><td>1min</td></tr>
<tr><td>final extension</td><td>68°C</td><td>7min</td><td>1</td></tr>
</table>

- Set up second PCR as below

**reagent**	**volume**	**final concentration**
10xThermoPol® buffer	2μl	1x
10mM dNTP	0.4μl	200μM
OF (10μM)	0.4μl	200nM
OR (10μM)	0.4μl	200nM
first PCR	1μl	
Taq polymerase 5U/μl	0.1μl	0.025U/μl
filtered milli-Q water	15.7μl	

<table>
<tr><th>step</th><th>temperature</th><th>time</th><th>no. of cycle</th></tr>
<tr><td>initial denaturation</td><td>94°C</td><td>2min</td><td>1</td></tr>
<tr><td>denaturation</td><td>94°C</td><td>25s</td><td rowspan="3">30</td></tr>
<tr><td>annealing</td><td>59°C</td><td>35s</td></tr>
<tr><td>extension</td><td>68°C</td><td>1min</td></tr>
<tr><td>final extension</td><td>68°C</td><td>7min</td><td>1</td></tr>
</table>

3.7 Clean PCR products

Materials:

- CleanSweep™ #A29895 (Applied Biosystems)
- Heat block (Grant) or water bath (Grant) at 37ºC
- Heat block (Grant) at 80ºC

Methods:

- Add 2µl of CleanSweep™ to 5µl PCR product
- Incubate at 37ºC for 15min
- inactivate at 80ºC for 15min

3.8 Sequencing

Materials:

- Sequencing primer (4µM) IF, IR, OF, OR
- filtered milli-Q water: filter through 0.22mm filter E4780-1223 (STARLAB)
- Software: Sequence viewer e.g. Chromas

Methods:

- set up the sequencing reaction as follow in a 1.5ml Eppendorf tube

**reagent**	**volume**	**final concentration**
cleaned PCR product	2µl	
sequencing primer (4µM) one primer from (IF, IR, OF, OR) per reaction	1µl	400nM
filtered milli-Q water	7µl	
**final volume**	**10µl**	

- Send to the Sanger sequencing services from GATC (Eurofins Genomics).
- Open sequence files in sequence viewer e.g. Chromas (NOTE: other popular viewers include BioEdit, Finch TV and 4Peaks for Mac)
- Export sequence in FASTA format using export or use 'Copy sequence' 'FASTA format' and save to notepad for downstream analysis

4. Design of PHB2 CRISPR RNA (crRNA) to generate single guide RNA (sgRNA)

Successful CRISPR knockout depends largely on the design of crRNA with good on-target and low off-target activities.

4.1 Manual search for crRNA (principal behind crRNA)

- Target exon 1 as it is more likely to generate non-functional protein. You can extend the target sequence slightly into the intronic region of the genomic DNA if there is a 5'-NGG-3' protospacer adjacent motif (PAM) near the 3' end of exon 1
- Search for PAM in the sense strand. The crRNA is 20 nucleotides before the PAM in 5' to 3' orientation e.g. 5'-GCTGTCGGTGCCGCTGCCTACGG-3' and the crRNA is 5'-GCTGTCGGTGCCGCTGCCTA-3'
- For anti-sense strand, you need to obtain the complement of the sense strand (section 2.3; select 'complement'), which now runs in the 3' to 5' orientation e.g. 3'-GGCCGAACTTCGACGACCGTCGA-5'
- PAM runs in 5' to 3' so you need to read from the 5' end (right to left). Looking at the sequence you will see 3'-GGN-5'. The crRNA reads from 5' to 3' so the crRNA is to the right of the PAM i.e. 3'-GGN-crRNA-5'. Read the 20 nucleotides from 5' to 3' to

obtain the crRNA e.g. 3’-GGCCGAACTTCGACGACCGTCGA-5’. Reverse the sequence (section 2.3; select ‘reverse’) and the crRNA is 5’-AGCTGCCAGCAGCTTCAAGC-3’.

4.2 Use Design tool Cas-Designer and Cas-OFFinder

- Go to CRISPR RGEN Tools (http://www.rgenome.net)
- Select Cas-Designer
- Input the exon 1 sequence into ‘Target Sequence’ box. Because the out-of-frame score is calculated based on the flanking sequence so include >30 extra flanking bases (from the 5’ untranslated region and the intronic region of the genomic DNA) to the exon 1 sequence. See section 3.5 to see how to add extra bases to the exon 1 sequence
- select crRNA length as 20 and PAM type as SpCas9 from Streptococcus pyogenes: 5’NGG-3’
- Select the organism (in our case it is ‘Insects’ then ‘Genomes’ Aedes albopictus (C6/36)-Asian tiger mosquito OR Aedes albopictus (JXUM01)-Asian tiger mosquito
- Click submit
- It will return a table of crRNAs with GC content, out-of-frame score and up to 2 number of mismatches and whether the target sequence is on plus or minus strand
- The Cas-Designer greys out the target sequences with 4 ‘T’ that can cause termination by RNA polymerase III. The recommended GC content is 20-80%. The recommended out-of-frame score is 66. De-select sequences with one 0 mismatch which means they exhibit single nucleotide polymorphism (SNP). Select sequences with two 0 mismatches which means they completely match their two target sequences in the two alleles; confirming the absence of SNP
- To set the cut-off of 3 mismatches in crRNA design, use Cas-OFFinder. Go to CRISPR RGEN Tools (http://www.rgenome.net). Select Cas-OFFinder
- Input all predicted crRNAs into the ‘Query Sequences’ box and ‘PAM type’ as SpCas9 from Streptococcus pyogenes: 5’NGG-3’. Select mismatch no=3, DNA bulge size=0, RNA bulge size=0 (you can change these criteria to suit your purpose)
- Select the organism (in our case it is ‘Insects’ then ‘Genomes’ Aedes albopictus (C6/36)-Asian tiger mosquito OR Aedes albopictus (JXUM01)-Asian tiger mosquito
- Click submit
- It will return a result table for the number of mismatches and the number of targets having that mismatch and also a table for the match/mismatch alignment with on-targets and off-targets. You can find PAM-proximal mismatches here
- Select crRNAs with two 0 mismatches (2 on-site targets without SNP) and ≤5 targets with up to 3 mismatches (off-targets)
- Due to the possibility of using alternative ATG start codon after disrupting 5’ end sequence, select crRNAs near the 3’ end of exon 1
- Choose the crRNA with more PAM-proximal mismatches. PAM-proximal mismatches reduces the possibility of binding to off-targets.

After CRISPR knockout of target gene (section 8.5), you can use the Cas-OFFinder results to design primers for checking off-target effects in knockout cells without or preceding the use of costly next-generation sequencing.

- Go to Primer-BLAST https://www.ncbi.nlm.nhs.gov/tools/primer-blast/index.cgi
- Input the accession number of the off-target, which can be found under ‘Chromosome’ of the Cas-OFFinder result table, as an input PCR template. Set the primer range to

500 bases 5’ and 3’ to the off-target position. Set PCR product size 200-1000. Select ‘Genomes for selected eukaryotic organisms (primary assembly only)’ then select ‘Aedes albopictus (Skuse, 1894) (taxid:7160)’. Press ‘Get Primers’ button

- Choose the primer pair which produces a short PCR product that covers the off-target site

NOTE:

A number of company- and academic-supported design tools are available but many are limited to commonly studied organisms such as human, mouse, rat, zebra fish and *Caenorhabditis elegans*. Several tools support a wide range of species including mosquitoes and pathogens but not cell lines:

- Benchling (https://www.benchling.com/crispr) supports *Ae. aegypti*, *Anopheles gambiae* and *Culex quinquefasciatus* but can request to add organisms
- e-CRISP (http://www.e-crisp.org) supports *Ae. aegypti* and *An. gambiae* and *An. darlingi* but can make suggestion
- CHOPCHOP (http://chopchop.cbu.uib.no/) supporting *Ae. albopictus* mosquito but not cell lines
- CRISPOR (http://crispor.tefor.net) supporting *Ae. albopictus* mosquito but not cell lines
- CRISPR GuideXpress (https://www.flyrnai.org/tools/fly2mosquito/web/) for single and batch sgRNA design in mosquitoes and mosquito cell lines but is limited to genomic sequences and annotation files available from the VectorBase (http://vectorbase.org).

5. Cloning of PHB2 crRNA oligonucleotides to generate sgRNA plasmid

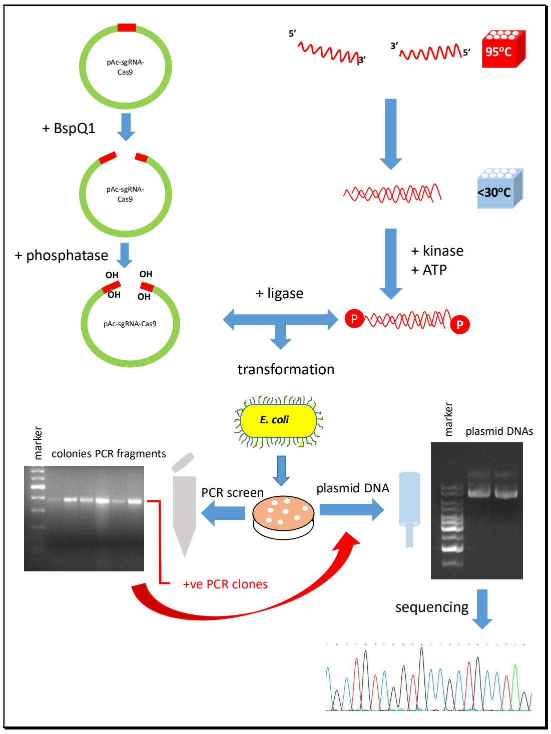

5.1 Annealing of crRNA oligonucleotides

Materials:

- We choose 5’-GCTGTCGGTGCCGCTGCCTA-3’ as our crRNA

- Add TTC to the sense primer and AAC to the antisense primer to create BspQ1 sites
- Since the crRNA sequence contains a 5’ terminal G, it was not necessary to add an additional G to the 5’ end to allow transcription from the dU6-2 promoter
- Order the following primers from Eurofins Genomics (or your preferred source)

primer	sequence	concentration
AlbopPHB2sgRNAF	5’-TTCGCTGTCGGTGCCGCTGCCTA-3’	100μM
AlbopPHB2sgRNAR	5’-AACTAGGCAGCGGCACCGACAGC-3’	100μM

- T4 polynucleotide kinase (PNK) 10U/μl #M0201S (New England Biolabs)
- 10x PNK buffer (700mM Tris-HCl, 100mM $MgCl_2$, 50mM DTT, pH 7.6@25°C) supplied with PNK #B02015 (New England Biolabs)
- 10mM ATP #A2383 (Sigma-Aldrich)
- Tris #BP152-1 (Fisher)
- EDTA #E5134 (Sigma-Aldrich)
- NaCl #S3160/65 (Fisher Scientific)
- 2x annealing buffer (20mM Tris-HCl, 2mM EDTA, 100mM NaCl, pH8)
- autoclaved milli-Q water
- heat block (Grant) set at 95°C
- 37°C water bath (Grant) or heat block (Grant)
- thermometer
- ice

Methods:

- Pre-heat a heat block to 95°C
- Set up an annealing reaction as below:

**reagent**	**volume**	**final concentration**
2x annealing buffer	10μl	1x
sense oligonucleotide (100μM) AlbopPHB2sgRNAF	5μl	25μM
anti-sense oligonucleotide (100μM) AlbopPHB2sgRNAR	5μl	25μM

- Place in a 95°C heat block for 5min
- remove heat block from heating unit and place at RT for 2-3h to let cool to RT. Monitor with a thermometer on the heat block. In our case, the temperature dropped to 40°C after 1h, 30°C after 2h and 23°C after 3h. Put on ice
- Set up a phosphorylation reaction of the annealed oligonucleotides as below:

**reagent**	**volume**	**final concentration**
annealed oligonucleotides from above	1μl	
10x PNK buffer	1μl	1x
ATP (10mM)	1μl	1mM
T4 PNK(10U/μl)	1μl	1U/μl
milli-Q water (autoclaved)	6μl	

- Incubate at 37°C for 30min
- Dilute the phosphorylated reaction 1:10 in autoclaved milli-Q water

5.2 Digestion and dephosphorylation of plasmid vector to create cloning sites

Materials:

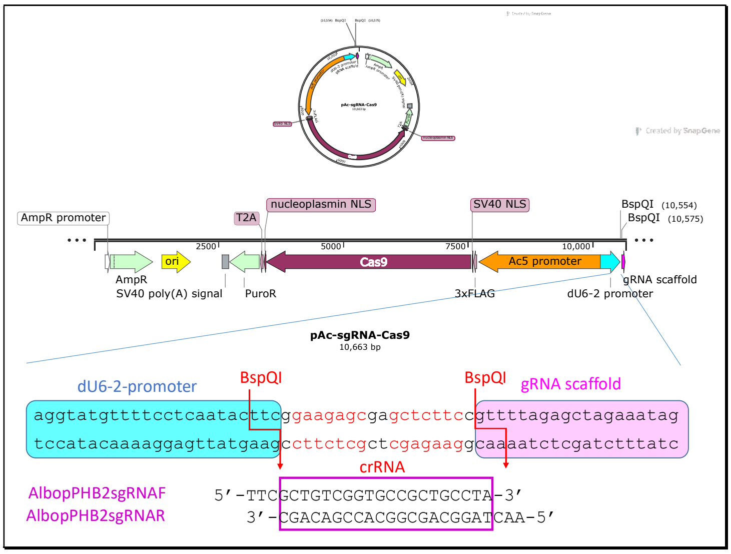

- The insect plasmid vector pAc-sgRNA-Cas9 which contains BspQ1 cloning sites and trans-acting crRNA (tracrRNA) downstream of the dU6-2 promoter and Cas9 downstream of the actin-5c promoter was purchased from Addgene (plasmid #49330)

NOTE: our methods are efficient in silencing genes in mosquito cell lines using *Drosophila* promoter-based CRISPR-Cas9 system. You can upgrade to mosquito promoter-based CRISPR-Cas9 system

- QIAprep Spin Miniprep Kit #27104 (Qiagen)
- BspQ1 (10U/µl) #R0712S (New England Biolabs)
- 10x NEB3.1 buffer #B6003S (New England Biolabs) supplied with the enzyme (1M NaCl, 500mM Tris-HCl, 100mM $MgCl_2$, 1mg/ml recombinant albumin, pH 7.9 @ 25°C)
- Anza™ Alkaline Phosphatase 1U/µl #IVGN220-4 (Invitrogen)
- tryptone #T1332 (Melford)
- yeast extract #Y20020 (Melford)
- agar #A20020 (Melford)
- NaCl #S3160/65 (Fisher Scientific)
- ampicillin #A9518 (Sigma-Aldrich)
- Luria Broth (LB): 1% tryptone, 0.5% yeast extract, 1% NaCl in milli-Q-water; autoclaved
- LB-agar: 1.5% agar in LB
- LB sterile in universals
- L-amp plate (LB-agar plate supplemented with 100µg/ml ampicillin
- agarose gel (section 3.4)
- autoclaved glycerol #G/0650/08 (Fisher)
- autoclaved milli-Q water
- 2ml cryo-vials #E3110-6122 (STARLAB)
- heat blocks (Grants) or water baths (Grants) at 37°C, 50°C and 80°C
- 37°C orbital shaker (Innova 4300, New Brunswick Scientific)

- 37ºC incubator
- NanoDrop ND-1000 (section 3.3)

Methods:

- the plasmid came as agar stab. To recover plasmid, streak the bacteria onto L-amp plate and incubate in a 37ºC incubator overnight to obtain single colonies
- Prepare 5ml sterile LB in a universal. Add 5µl of 100mg/ml ampicillin to a final concentration of 100µg/ml.
- Pick single colonies into individual universals. Put several universals in 37ºC orbital shaker for 6-8h to prepare 15% glycerol stocks in cryo-vials and stored as stocks at -80ºC.
- Put a couple of universals in 37ºC orbital shaker overnight for plasmid DNA preparation using the QIAprep Spin Miniprep Kit (Qiagen) as described in the manufacturer's instructions.
- NanoDrop to check DNA concentration and purity (section 3.3)
- Run 1µl of plasmid DNA on 0.7% TAE agarose gel to check DNA integrity (section 3.4). 100V 1h
- Set up a digestion reaction as below

reagent	volume	final concentration
10x NEB3.1 buffer	5µl	1x
plasmid DNA	xµl=2µg	
BspQ1 (10U/µl)	2µl	0.4U/µl
milli-Q water (autoclaved)	make up to a final volume of 50µl	

- Incubate at 50ºC for at least 1h (we used 2.5h)
- Incubate at 80ºC for 20min to inactivate BspQ1
- Add 1µl of alkaline phosphatase to the digestion reaction at 37ºC for 10min to dephosphorylate the cut plasmid
- Incubate at 80ºC for 5min to inactivate alkaline phosphatase

5.3 Ligation and transformation

Materials:

- Competent *Escherichia coli* (Section 5.5)
- Anza™ T4 DNA Ligase Master Mix #IVGN210-4 (Invitrogen)
- tryptone #T1332 (Melford)
- yeast extract #Y20020 (Melford)
- agar #A20020 (Melford)
- NaCl #S3160/65 (Fisher Scientific)
- ampicillin #A9518 (Sigma-Aldrich)
- Luria Broth (LB): 1% tryptone, 0.5% yeast extract, 1% NaCl in milli-Q-water; autoclaved
- LB-agar: 1.5% agar in LB
- pre-warmed LB
- L-amp plate (LB-agar plate supplemented with 100µg/ml ampicillin
- milli-Q water (autoclaved)
- ice
- 42ºC water bath (Grant)

- 37ºC orbital shaker (Innova 4300, New Brunswick Scientific)
- 37ºC incubator

Methods:

- Set up the ligation reaction as below:

**reagent**	**volume**	**final concentration**
cut plasmid vector	1.7µl	
annealed oligonucleotides (diluted 1:10)	2µl	
milli-Q water	3.8µl	
Anza™ T4 DNA Ligase Master Mix	2.5µl	1x

- Incubate at RT for 1h
- Meanwhile, thaw 100µl aliquots of competent *Escherichia coli* and then put on ice
- Add 2µl of ligation mix to 100µl competent *Escherichia coli* on ice. Tap to mix
- Incubate on ice for 20min
- Transfer to a 42ºC water bath and heat shock for 30s
- Put back on ice for 2min
- Add 500µl pre-warmed LB to the transformation mix. Put in an orbital shaker at 37ºC for 1h
- Plate out dilutions (e.g. 1µl, 100µl). Spin down the rest, re-suspend pellet in 100µl LB and plate onto L-amp plate
- Put in a 37ºC incubator and grow overnight for colonies to appear.

5.4 Screening of colonies for insert

Materials:

- Order the following primers from Eurofins Genomics (or other sources)

primer	sequence	concentration
pAcSeqF	5'-GCCGAGTCAAATGCCGAATG-3'	100µM
AlbopPHB2sgRNAR	5'-AACTAGGCAGCGGCACCGACAGC-3'	100µM

- QIAprep Spin Miniprep Kit #27104 (Qiagen)
- *Taq* DNA polymerase 5U/µl #M0267S (New England Biolabs)
- 10xThermoPol® buffer containing 200mM Tris-HCl, 100mM $(NH_4)_2SO_4$, 100mM KCl, 20mM $MgSO_4$, 1% Triton® X-100 pH 8.8@25°C #B9004 (supplied with *Taq* DNA polymerase)
- 10mM dNTP #1277049 (Roche)
- tryptone #T1332 (Melford)
- yeast extract #Y20020 (Melford)
- agar #A20020 (Melford)
- NaCl #S3160/65 (Fisher Scientific)
- ampicillin #A9518 (Sigma-Aldrich)
- Luria Broth (LB): 1% tryptone, 0.5% yeast extract, 1% NaCl in milli-Q-water; autoclaved
- LB-agar: 1.5% agar in LB
- sterile LB in universals
- L-amp plate (LB-agar plate supplemented with 100µg/ml ampicillin

- TAE agarose gel (section 3.4)
- filtered milli-Q water: filter through 0.22mm filter E4780-1223 (STARLAB)
- PCR machine MULTIGENE™ MINI (Labnet)
- 37ºC incubator
- 37ºC orbital shaker (Innova 4300, New Brunswick Scientific)
- NanoDrop ND-1000 (Thermo Scientific) (section 3.3)

Methods:

- Prepare master PCR mix as follows. To make up n reactions, prepare a master mix for (n+1) reactions

**reagent**	**volume for 1 reaction**	**master mix (n+1)**	**final concentration**
10xThermoPol® buffer	2µl	2µl x (n+1)	1x
10mM dNTP	0.4µl	0.4µl x (n+1)	200µM
pAcSeqF (10µM)	0.4µl	0.4µl x (n+1)	200nM
AlbopPHB2sgRNAR (10µM)	0.4µl	0.4µl x (n+1)	200nM
Taq polymerase 5U/µl	0.1µl	0.1µl x (n+1)	0.025U/µl
filtered milli-Q water	16.7µl	16.7µl x (n+1)	

- Aliquot 20µl of master mix into individual 0.5ml PCR tubes.
- Pick single colonies and stir into individual PCR tubes and then streak out onto a master L-amp plate and incubate overnight at 37ºC incubator to keep as stocks. Start the PCR as below:

**step**	**temperature**	**time**	**no. of cycle**
**initial denaturation**	94ºC	2min	1
**denaturation**	94ºC	25s	
**annealing**	59ºC	35s	30
**extension**	68ºC	1min	
**final extension**	68ºC	7min	1

- Check PCR products on 1% TAE agarose gel electrophoresis for insert (section 3.4). 100V for 1h
- Recover positive colonies from the master plate by streaking onto new L-amp plates to isolate single colonies. Prepare plasmid DNA for sequencing
- Prepare 5ml sterile LB in a universal. Add 5µl of 100mg/ml ampicillin to a final concentration of 100µg/ml
- Pick single colonies into individual universals. Put in a 37ºC orbital shaker overnight for plasmid DNA preparation using the QIAprep Spin Miniprep Kit (Qiagen) as described in the manufacturer's instructions
- Run 1µl of plasmid DNA on 0.7% TAE agarose gel to check DNA integrity (section 3.4). 100V 1h
- NanoDrop to check DNA concentration and purity (section 3.3)
- Sequence 500ng of the plasmid DNA using primer pAcSeqF (section 3.8)

<u>5.5 Preparation of competent cells</u>

Materials:

- *Escherichia coli* (TG1) (or any other suitable strain)
- autoclaved 0.1M $MgCl_2$ #M/0600/53 (Fisher); ice-cold

- autoclaved 0.1M $CaCl_2$ #C/1500/53 (Fisher); ice-cold
- autoclaved glycerol #G/0650/08 (Fisher)
- tryptone #T1332 (Melford)
- yeast extract #Y20020 (Melford)
- agar #A20020 (Melford)
- NaCl #S3160/65 (Fisher Scientific)
- Luria Broth (LB): 1% tryptone, 0.5% yeast extract, 1% NaCl in milli-Q-water; autoclaved
- LB-agar: 1.5% agar in LB
- sterile universals
- LB-agar plates
- sterile 50ml tubes (Falcon)
- autoclaved conical flasks (PYREX)
- ice
- spectrophotometer Ultrospec 2100*pro* (Amersham BioSciences)
- refrigerated centrifuge Megafuge 40R (Thermo Scientific)
- 37ºC incubator
- 37ºC orbital shaker (Innova 4300, New Brunswick Scientific)

Methods:

- Streak out a glycerol stock of the *E. coli* TG1 on LB-agar plate to isolate single colonies. Grow in a 37ºC incubator overnight
- Inoculate a single colony in a universal with 5ml sterile LB in a 37ºC orbital shaker overnight
- Dilute the overnight culture 1:50 into a flask containing fresh LB and grow until the OD_{600} reached 0.7-0.9
- Put the flask on ice for 20min
- Transfer bacterial cells to Falcon tubes. Pellet bacterial cells at 3500rpm/4ºC for 15min
- re-suspend pellet in half of the original volume of ice-cold 0.1M $MgCl_2$
- Pellet bacterial cells at 3500rpm/4ºC for 15min
- re-suspend pellet in $1/20^{th}$ of the original volume of ice-cold 0.1M $CaCl_2$
- Put the Falcon tube on ice for a minimum of 30min before use
- Add glycerol to 15% glycerol for long-term storage at -80ºC

6. Selection of knockout cells

<u>6.1 Kill curve</u>

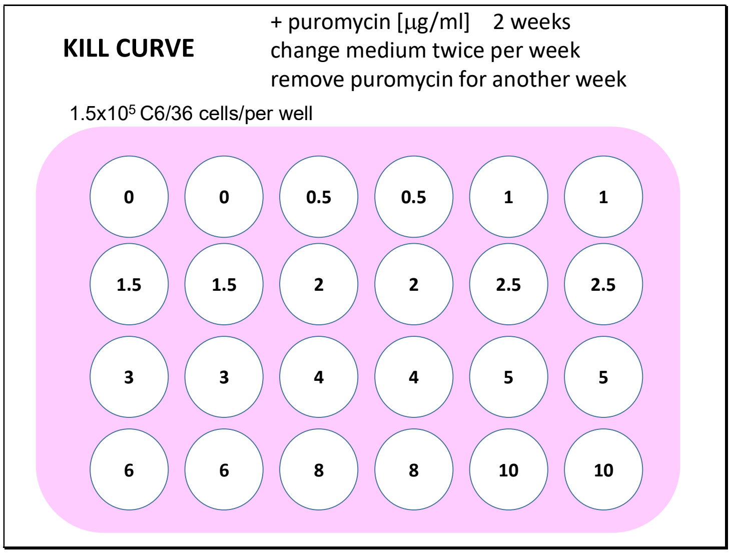

Materials:

- C6/36 cells (ATCC CRL-1660™)
- C6/36 growth medium: Eagle's minimal essential medium (MEM) #M4655 (Sigma-Aldrich) supplemented with 1x non-essential amino acid (NEAA) #M7145 (Sigma-Aldrich), 10% fetal calf serum (FCS) #16500-064 (Gibco) and 100 U/ml penicillin, and 100mg/ml streptomycin #P0781 (Sigma-Aldrich)
- puromycin #P-7255 (Sigma-Aldrich)
- 24-well tissue culture plate Costar® (Corning)
- CO_2 incubator at 28°C and 5% CO_2 (MiniGalaxy E)

Methods:

- Seed C6/36 cells at 1.5x10^5 cells/well of a 24-well plate overnight
- Replace medium in duplicate wells with growth medium containing 0µg/ml to 10µg/ml puromycin
- Change the medium to fresh medium containing puromycin twice per week for 2 weeks
- Change the medium to fresh medium without puromycin for another week
- Select the concentration of puromycin in the well without cell growth

6.2 Test for transfection efficiency

Materials:

- C6/36 cells (ATCC CRL-1660™)
- growth medium without antibiotics: Eagle's minimal essential medium (MEM) #M4655 (Sigma-Aldrich) supplemented with 1x non-essential amino acid (NEAA) #M7145 (Sigma-Aldrich), 10% fetal calf serum (FCS) #16500-064 (Gibco)
- a plasmid encoding β-galactosidase pCMV-β-gal (Clontech)
- transfection reagents
  NOTE: Transfection reagents tested include calcium phosphate (1, 2), FuGENE® HD #E2311 (Promega), XtremeGene HP #06366244001 (Roche), XtremeGene 9 #06366511001 (Roche), Trans-IT 2020 #MIR5410 (Mirus), Trans-IT X2 #MIR6010 (Mirus), Trans-IT Insect Transfection Reagent #MIR6110 (Mirus), PolyFect #301107 (Qiagen), SuperFect #301307 (Qiagen), Effectene #301425 (Qiagen), DreamFect™

#DF40500 (Oz BioSciences), JetPRIME® #114-01 (Polyplus) and Lipofectamine 3000 #L3000-008 (Invitrogen)

1. Chan SW. Fusion assays for screening of fusion inhibitors targeting SARS-CoV-2 entry and syncytia formation. Front Pharmacol. 2022;13:1007527.
2. Chan SW, Shafi T, Ford RC. Kite-Shaped Molecules Block SARS-CoV-2 Cell Entry at a Post-Attachment Step. Viruses. 2021;13(11).

- glutaraldehyde #G5882 (Sigma-Aldrich) 0.5% make up in PBS
- 40x (40mg/ml) X-Gal (5-bromo-4-chloro-3-indolyl-β-D-galactoside) #BIO-37035 (Bioline) in dimethyl formamide #227056 (stored at -20ºC)
- $K_4Fe(CN)_6$ #P9387 (Sigma-Aldrich)
- $K_3Fe(CN)_6$ #P8131 (Sigma-Aldrich)
- $MgCl_2$ #M/0600/53 (Fisher)
- PBS #P4417-100TAB (Sigma-Aldrich) made up one tablet in 200ml milli-Q water; autoclaved
- X-Gal solution: 1x (1mg/ml) X-Gal, 5mM $K_4Fe(CN)_6$, 5mM $K_3Fe(CN)_6$, 2mM $MgCl_2$ in PBS; filter through 0.2µm filter E4780-1223 (STARLAB)
- 24-well tissue culture plate Costar® (Corning)
- CO_2 incubator at 28°C and 5% CO_2 (MiniGalaxy E)
- CO_2 incubator at 37°C and 5% CO_2 (HERA Cell, Heraeus)
- microscope (WPI)

Methods:

- Seed C6/36 cells at $3x10^5$/0.5ml in MEM growth medium without antibiotics in 24-well plates overnight in 28°C/5% CO_2 incubator
- Transfect cells with pCMV-β-gal for 48h according to manufacturer's instructions
- Observe any morphological changes and toxicity under the microscope
- Remove medium. Add 1ml 0.5% glutaraldehyde to each well at RT for 15min to fix cells
- Wash cells once with 1ml cold PBS. Add 1ml X-Gal solution to each well. Incubate at 37ºC/5%CO_2 incubator overnight
- Wash cells once with 1ml cold PBS. Add 1ml cold PBS to keep cells in
- Look at cells under the microscope for blue-stained cells.

<u>6.3 Transfection by XtremeGene 9 and Lipofectamine 3000</u>

Materials:

- C6/36 cells (ATCC CRL-1660™)
- growth medium without antibiotics: Eagle's minimal essential medium (MEM) #M4655 (Sigma-Aldrich) supplemented with 1x non-essential amino acid (NEAA) #M7145 (Sigma-Aldrich), 10% fetal calf serum (FCS) #16500-064 (Gibco)
- XtremeGene 9 #06366511001 (Roche)
- Lipofectamine 3000 #L3000-008 (Invitrogen)
- Opti-MEM™ medium #31985-062 (Gibco)
- 100mm tissue culture dish (Corning)
- CO_2 incubator at 28°C and 5% CO_2 (MiniGalaxy E)

Methods:

- Transfection with XtremeGene 9
    - Seed C6/36 cells at $9x10^6$ cells per 100mm dish in 10ml MEM growth medium without antibiotics overnight
    - Add 15µl XtremeGene 9 to 500µl Opti-MEM™. Tap to mix
    - Add 5µg plasmid DNA to the above mix. Tap to mix. Set up 2 reactions: one using the plasmid with sgRNA and a control one using empty plasmid vector
    - Incubate at RT for 15min
    - Add dropwise to dish. Swirl to mix
    - Return to 28°C/5%CO_2 incubator for 48h
- transfection with Lipofectamine 3000
    - Seed C6/36 cells at $9x10^6$ cells per 100mm dish in 10ml MEM growth medium without antibiotics overnight
    - Dilute 15µg of the plasmid DNA in 750µl Opti-MEM™ medium (Gibco) and 30µl of the P3000™ Reagent (Invitrogen). Tap to mix. Set up 2 reactions: one using the plasmid with sgRNA and a control one using empty plasmid vector
    - Dilute 45µl of Lipofectamine™ 3000 (Invitrogen) in 750µl Opti-MEM™ medium. Tap to mix
    - Add the first mixture containing the plasmid DNA to the second mixture. Tap to mix
    - Incubation at RT for 15min
    - Add the transfection mix dropwise to the cell culture. Swirl to mix
    - After 6h of incubation, add 5ml of MEM growth medium without antibiotics to the cell culture to dilute out the transfection reagent
    - Transfect for a total of 48h.

7. Selection of CRISPR-edited clones

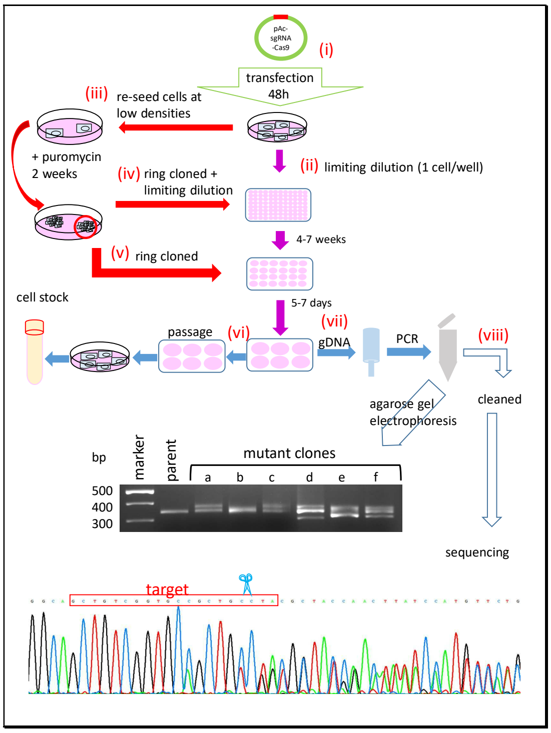

7.1 Selection of cell clones

Materials:

- C6/36 cells (ATCC CRL-1660™)
- growth medium without antibiotics: Eagle's minimal essential medium (MEM) #M4655 (Sigma-Aldrich) supplemented with 1x non-essential amino acid (NEAA) #M7145 (Sigma-Aldrich), 10% fetal calf serum (FCS) #16500-064 (Gibco)
- XtremeGene 9 #06366511001 (Roche)
- Lipofectamine 3000 #L3000-008 (Invitrogen)
- trypsin #T3924 (Sigma-Aldrich)
- puromycin #P-7255 (Sigma-Aldrich)
- 100mm tissue culture dish, 96-well plate, 24-well plate, 6-well plate (Corning)
- cell scraper #08100240 (Fisher Scientific)
- CO_2 incubator at 28°C and 5% CO_2 (MiniGalaxy E)
- microscope (WPI)

Methods:

- Seed C6/36 cells at a cell density of $9x10^6$ per 100mm dish in a 28°C/5%CO_2 incubator overnight
- Transfect C6/36 cells with either the plasmid with sgRNA or a control plasmid vector; using either XtremeGene-9 or Lipofectamine (section 6.3)
- After 48h of transfection, cells were trypsinzed and selected in two ways:
- Limiting dilution:
  - Seed transfected cells at one cell/well of a 96-well plate by limiting dilution. Monitor appearance of single cell clones daily by microscopy
  - when cells in 96-well plate become confluence (took about 4-7 weeks), trypsinize cells and transfer cells in each well to a 24-well plate
  - When cells in 24-well plate become confluent (took 5-7 days), trypsinize cells and transfer cells in each well to a 6-well plate
- Puromycin selection:
  - Re-seed transfected cells at low densities ($4.5x10^6$; $2.25x10^6$; $1.125x10^6$; $0.56x10^6$ per 100mm dish). After an overnight incubation, add 2µg/ml puromycin to each dish. Replenish puromycin by changing medium + puromycin twice per week for two weeks. Remove puromycin after 2 weeks and continue culture in growth medium without puromycin until colonies appear.
  - Isolate individual colonies by ring cloning
    - Purify colonies by seeding at one cell/well of a 96-well plate by limiting dilution OR
    - directly transfer into 24-well plate
- When cells become confluent in 6-well plate, replace medium with 2ml of fresh growth medium
- Dislodge cells by pipetting with a blue tip or by using a cell scraper
- Transfer 1ml of the medium containing dislodged cells to an Eppendorf tube for genomic DNA extraction (section 3.2)
- Add another 1ml of fresh medium to the well to keep cell passage until INDELs are identified (section 7.2). Then, make cell stocks and stored in liquid nitrogen

7.2 INDELs detection by agarose gel electrophoresis

Materials:

- Order the following primers from Eurofins Genomics (or your preferred source)

primer	sequence	concentration
IF AeAlbop (IF)	5'-ATATTCCGGCGCGGTGATTG-3'	10μM
IR AeAlbop (IR)	5'-CCCCAATCAAGTTCCAGCGG-3'	10μM

- *Taq* DNA polymerase 5U/μl #M0267S (New England Biolabs)
- 10xThermoPol® buffer containing 200mM Tris-HCl, 100mM $(NH_4)_2SO_4$, 100mM KCl, 20mM $MgSO_4$, 1% Triton® X-100 pH 8.8@25°C #B9004 (supplied with *Taq* DNA polymerase)
- 10mM dNTP #1277049 (Roche)
- TAE agarose gel (section 3.4)
- filtered milli-Q water: filter through 0.22mm filter E4780-1223 (STARLAB)
- PCR machine MULTIGENE™ MINI (Labnet)

Methods:

- Set up PCR as below. Include a PCR from parental C6/36 genomic DNA as a control.

**reagent**	**volume**	**final concentration**
10xThermoPol® buffer	2μl	1x
10mM dNTP	0.4μl	200μM
IF (10μM)	0.4μl	200nM
IR (10μM)	0.4μl	200nM
genomic DNA	xμl (=1μg)	
Taq polymerase 5U/μl	0.1μl	0.025U/μl
filtered milli-Q water	make up to total volume of 20μl	

**step**	**temperature**	**time**	**no. of cycle**
**initial denaturation**	94°C	2min	1
**denaturation**	94°C	25s	
**annealing**	54°C	35s	30
**extension**	68°C	1min	
**final extension**	68°C	7min	1

- Separate PCR fragments by 2-3% TAE agarose gel electrophoresis for 1-2h at 100V (section 3.4). Comparing the sizes of the fragments with parental PCR fragments to identify INDELs
- Clean and sequence PCR products using primer IF (section 3.7, 3.8)

7.3 Prediction of INDELs by sequence decomposition

Since CRISPR acts on the two gene alleles independently to create random and different mutations, direct sequencing from genomic DNA will generate mixed sequences. A number of computer programs are available to decompose mixed sequences that will help predicting mono-allelic, bi-allelic and mixed knockout cell clones and cell clones retaining the wild type genes. Below are some examples:

- Tracking of INDELs by DEcomposition (TIDE) (http://tide.nki.nl) predicts the number and percentage of INDELs but not the sequences. It allows users to change the decomposition criteria

- CRISPR-ID (http://crispid.gbiomed.kuleuven.be) allows users to change the decomposition criteria
- Inference of CRIPSR Edits (ICE) (http://ice.synthego.com) user-friendly with pre-set criteria
- DEconvolution of Complex DNA Repair (DECODR) (https://decodr.org) user-friendly with pre-set criteria
- Tracy Indigo application (http://www.gear-genomics.com) user-friendly with pre-set criteria

8. Characterization of CRISPR-edited cell clones

- Mono- and bi-allelic knockout cell clones are the end-products and can be used for functional study subject to:
    - Confirm sequences of the two gene alleles by molecular cloning into a sequencing vector
    - Assess the expression level of the knockout/knockdown protein by Western blotting and at single cells level by immunocytochemistry.
- Mono-allelic knockout clones can also be re-CRISPRed to target the remaining wild type allele, if required.
- Mixed clones can be further purified using limiting dilution and then re-characterized as above.

8.1 Molecular cloning into pBluescript for sequencing

Materials:

- Order the following primers from Eurofins Genomics (or your preferred source)

primer	sequence	concentration
IF AeAlbop (IF)	5’-ATATTCCGGCGCGGTGATTG-3’	10µM
IR AeAlbop (IR)	5’-CCCCAATCAAGTTCCAGCGG-3’	10µM

- CRISPR-edited cell clone
- PureLink® Genomic DNA Kits #K1820-02 (Invitrogen) (section 3.2)
- NucleoSpin Gel and PCR Clean Up Kit #740609.50 (Machery Nagel)
- Q5 DNA polymerase #M0491S (New England Biolabs)
- 5x Q5 buffer supplied with the polymerase proprietary formula containing 2mM Mg^{2+} #B9027S
- 10mM dNTP #1277049 (Roche)
- TAE agarose gel (section 3.4)
- filtered milli-Q water: filter through 0.22mm filter E4780-1223 (STARLAB)
- autoclaved milli-Q water
- PCR machine MULTIGENE™ MINI (Labnet)
- NanoDrop 1000 (Thermo Scientific) (section 3.3)

Methods:

- Extract genomic DNA from the CRISPR-edited cell clone using PureLink® Genomic DNA Kits (Invitrogen) according to the manufacturer’s instructions (section 3.2)

- Check 1µl genomic DNA on 1% TAE agarose gel electrophoresis (section 3.4). 100V for 1h
- NanoDrop to measure DNA concentration and purity (section 3.3)
- Set up PCR as follows:

reagent	volume	final concentration
5x Q5 buffer	10µl	1x
10mM dNTP	1µl	200µM
IF (10µM)	2.5µl	500nM
IR (10µM)	2.5µl	500nM
genomic DNA	xµl (=1µg)	
Q5 polymerase 2U/µl	0.5µl	0.02U/µl
filtered milli-Q water	make up to a final volume of 50µl	

step	temperature	time	no. of cycle
**initial denaturation**	98°C	30s	1
**denaturation**	98°C	10s	30
**annealing**	69°C	30s	
**extension**	72°C	30s	
**final extension**	72°C	2min	1

- Check 5µl PCR products on 1% TAE agarose gel electrophoresis (section 3.4). 100V for 1h
- Clean up the PCR products using NucleoSpin Gel and PCR Clean Up Kit (Machery Nagel) according to the manufacturer's instructions. Elute in 25µl autoclaved milli-Q water.
- Check 2µl on 1% TAE agarose gel electrophoresis (section 3.4). 100V for 1h
- NanoDrop to measure DNA concentration and purity (section 3.3)

8.2 Digestion of pBluescript to create blunt-ends

Since the PCR products from Q5 polymerase amplification are blunt-ended, we need to create blunt ends in pBluescript plasmid

Materials:

- pBluescript (Stratagene) plasmid DNA
- NucleoSpin Gel and PCR Clean Up Kit #740609.50 (Machery Nagel)
- *EcoR*V (10U/µl) #R6351 (Promega)
- 10x Buffer D #R004A (Promega) (60mM Tris-HCl, 60mM $MgCl_2$, 1.5M NaCl, 10mM DTT, pH7.9@37°C) supplied with *EcoR*V
- acetylated bovine serum albumin (10mg/ml) #R396D (Promega) supplied with *EcoR*V
- Anza™ Alkaline Phosphatase #IVGN220-4 (Invitrogen) 1U/µl
- Anza™ 10x buffer supplied with Anza™ Alkaline Phosphatase (Invitrogen)
- TAE agarose gel (section 3.4)
- milli-Q water (autoclaved)
- 37°C water bath (Grant)
- 80°C heat block (Grant)

- NanoDrop 1000 (Thermo Scientific) (section 3.3)

Methods:

- Set up a digestion reaction as below:

reagent	volume	final concentration
10x Buffer D	20µl	1x
acetylated BSA 10mg/ml	2µl	100µg/ml
pBluescript	xµl=4µg	
EcoRV (10U/µl)	10µl	0.5U/µl
milli-Q water (autoclaved)	make up to a final volume of 200µl	

- Incubate at 37ºC overnight
- Add 5µl *EcoR*V to the digestion reaction the following morning, if necessary
- Clean up the digestion using NucleoSpin Gel and PCR Clean Up Kit (Machery Nagel) according to the manufacturer's instructions. Elute in 50µl milli-Q water.
- NanoDrop to measure DNA concentration and purity.
- Set up phosphatase reaction as follows:

reagent	volume	final concentration
Anza™ 10x buffer	6µl	1x
cut pBluescript	xµl=2.5µg	
Anza™ Alkaline Phosphatase	3µl	0.05U/µl
milli-Q water (autoclaved)	make up to a final volume of 60µl	

- Incubate 37ºC for 15min
- Inactivate alkaline phosphatase at 80ºC for 5min
- Clean up the digestion using NucleoSpin Gel and PCR Clean Up Kit (Machery Nagel) according to the manufacturer's instructions. Elute in 25µl milli-Q water.
- Check 2µl on 1% TAE agarose gel electrophoresis. 100V for 1h
- NanoDrop to measure DNA concentration and purity (section 3.3)

8.3 Ligation and transformation

Materials:

- Competent *Escherichia coli* (section 5.5)
- Anza™ T4 DNA Ligase Master Mix #IVGN210-4 (Invitrogen)
- tryptone #T1332 (Melford)
- yeast extract #Y20020 (Melford)
- agar #A20020 (Melford)
- NaCl #S3160/65 (Fisher Scientific)
- ampicillin #A9518 (Sigma-Aldrich)
- X-Gal #BIO-37035 (Bioline) (40mg/ml) in dimethyl formamide #227056 (stored at -20ºC)
- IPTG (iso-propyl-β-D-thio-galactopyranoside) #BIO-37036 (Bioline) 80mM
- Luria Broth (LB): 1% tryptone, 0.5% yeast extract, 1% NaCl in milli-Q-water; autoclaved
- LB-agar: 1.5% agar in LB

- pre-warmed LB
- L-amp plate (LB-agar plate supplemented with 100µg/ml ampicillin)
- L-amp plate supplemented with 50µl of 40mg/ml X-Gal and 50µl of 80mM IPTG for blue-white screening
- milli-Q water (autoclaved)
- ice
- 42ºC water bath (Grant)
- 37ºC orbital shaker (Innova 4300, New Brunswick Scientific)
- 37ºC incubator

Methods:

- Set up the ligation reaction as below:
    - use an insert:vector molar ratio of 9:1
      NOTE: depending on the DNA concentrations, adjust the volumes of the insert and vector to give a 9:1 molar ratio and make up the final volume to 10µl with milli-Q water
    - Also set up a re-ligated control

**reagent**	**ligation**	**re-ligated control**	**final concentration**
cut plasmid vector	2.5µl	2.5µl	
PCR products	5µl	0µl	
milli-Q water	0µl	5µl	
Anza™ T4 DNA Ligase Master Mix	2.5µl	2.5µl	1x

- Incubate at RT for 30min
- Meanwhile, thaw 100µl aliquots of competent *Escherichia coli* and then put on ice
- Add 10µl of ligation mix to 100µl competent *Escherichia coli* on ice. Tap to mix
- Also set up a transformation control: add 2.5µl cut pBluescript to 100µl competent *Escherichia coli* on ice. Tap to mix
- Incubate on ice for 20min
- Transfer to a 42ºC water bath and heat shock for 30s
- Put back on ice for 2min
- Add 500µl pre-warmed LB to the transformation mix. Put in an orbital shaker at 37ºC for 1h
- Plate out dilutions (e.g. 1µl, 100µl). Spin down the rest, re-suspend pellet in 100µl LB and plate onto L-amp plate + Xgal + IPTG.
- Put in a 37ºC incubator and grow overnight for colonies to appear

<u>8.4 Screening of colonies for insert</u>

Materials:

- Order the following primers from Eurofins Genomics (or your preferred source)

primer	sequence	concentration
IF AeAlbop (IF)	5'-ATATTCCGGCGCGGTGATTG-3'	10µM
IR AeAlbop (IR)	5'-CCCCAATCAAGTTCCAGCGG-3'	10µM

- QIAprep Spin Miniprep Kit #27104 (Qiagen)

- *Taq* DNA polymerase 5U/µl #M0267S (New England Biolabs)
- 10xThermoPol® buffer containing 200mM Tris-HCl, 100mM $(NH_4)_2SO_4$, 100mM KCl, 20mM $MgSO_4$, 1% Triton® X-100 pH 8.8@25°C #B9004 (supplied with *Taq* DNA polymerase)
- 10mM dNTP #1277049 (Roche)
- tryptone #T1332 (Melford)
- yeast extract #Y20020 (Melford)
- agar #A20020 (Melford)
- NaCl #S3160/65 (Fisher Scientific)
- ampicillin #A9518 (Sigma-Aldrich)
- X-Gal #BIO-37035 (Bioline) (40mg/ml) in dimethyl formamide #227056 (stored at -20°C)
- IPTG #BIO-37036 (Bioline) 80mM
- Luria Broth (LB): 1% tryptone, 0.5% yeast extract, 1% NaCl in milli-Q-water; autoclaved
- universals with 5ml sterile LB plus 100µg/ml ampicillin
- LB-agar: 1.5% agar in LB
- L-amp plate (LB-agar plate supplemented with 100µg/ml ampicillin)
- L-amp plate supplemented with 50µl of 40mg/ml X-Gal and 50µl of 80mM iso-propyl-β-D-thio-galactopyranoside (IPTG) for blue-white screening
- TAE agarose gel (section 3.4)
- filtered milli-Q water: filter through 0.22mm filter E4780-1223 (STARLAB)
- PCR machine MULTIGENE™ MINI (Labnet)
- 37°C incubator
- 37°C orbital shaker (Innova 4300, New Brunswick Scientific)
- NanoDrop 1000 (Thermo Scientific) (section 3.3)

Methods:

- Prepare master PCR mix as follows. To make up n reactions, prepare a master mix for (n+1) reactions

**reagent**	**volume for 1 reaction**	**master mix (n+1)**	**final concentration**
10xThermoPol® buffer	2µl	2µl x (n+1)	1x
10mM dNTP	0.4µl	0.4µl x (n+1)	200µM
IF (10µM)	0.4µl	0.4µl x (n+1)	200nM
IR (10µM)	0.4µl	0.4µl x (n+1)	200nM
Taq polymerase 5U/µl	0.1µl	0.1µl x (n+1)	0.025U/µl
filtered milli-Q water	16.7µl	16.7µl x (n+1)	

- Aliquot 20µl of master mix into individual 0.5ml PCR tubes.
- Pick single white colonies and stir into individual PCR tubes and then streak out onto a master L-amp plate with X-gal and IPTG and incubate overnight at 37°C incubator to keep as colony stocks. Start the PCR as below:

**step**	**temperature**	**time**	**no. of cycle**
**initial denaturation**	94°C	2min	1
**denaturation**	94°C	25s	30
**annealing**	54°C	35s	
**extension**	68°C	1min	

**final extension**	68ºC	7min	1

- Check PCR products on 1% TAE agarose gel electrophoresis for insert (section 3.4). 100V for 1h
- Recover positive colonies from the master plate by streaking onto new L-amp plates with X-gal and IPTG to isolate single colonies. Prepare plasmid DNA for sequencing.
- Prepare 5ml sterile LB in a universal. Add 5µl of 100mg/ml ampicillin to a final concentration of 100µg/ml.
- Pick single colonies into individual universals. Put in a 37ºC orbital shaker overnight for plasmid DNA preparation using the QIAprep Spin Miniprep Kit (Qiagen) as described in the manufacturer's instructions.
- Run 1µl of plasmid DNA on 0.7% TAE agarose gel to check DNA integrity (section 3.4). 100V 1h
- NanoDrop to check DNA concentration and purity (section 3.3)
- Sequence 500ng of the plasmid DNA using primers IF or IR (section 3.8)

8.5 Detection of frameshift and truncation

- Open sequence files in sequence viewer e.g. Chromas (section 3.8)
- Export (or use copy sequence in FASTA format) and save to notepad
- Translate the nucleotides into amino acids using Expasy Translate (section 2.4)
- Use CLUSTAL Omega Multiple Sequence Alignment to align cloned sequences from CRISPR-edited cells with parental sequence to identify frameshift and truncation (section 2.5)

8.6 Western blotting

Materials:

- Cell cultures
- DPBS with Ca^{2+} and Mg^{2+} #D8662 (Sigma-Aldrich)
- Precision Plus Protein Dual Colour Standards #161-0374 (Bio-Rad)
- TGX Stain-Free SDS-PAGE gel #4568043 (Bio-Rad)
- polyvinylidene difluoride (PVDF) Immobilon-FL membranes #IPFL00010 (Millipore)
- 1:250 anti-PHB2 antibody #NBP2-13754 (Novus)
- 1:200 anti-rabbit horseradish peroxidase-conjugated secondary antibody #7074 (Cell Signaling Technology)
- Clarity™ ECL substrate #170-5061 (Bio-Rad)
- bicinchoninic acid (BCA) assay #BCA1+B9643 (Sigma-Aldrich)
- bovine serum albumin (protein standards) #A8806 (Sigma-Aldrich)
- semi-skimmed milk (Marvel)
- Tween 20 #P9416 (Sigma-Aldrich)
- Tris #BP152-1 (Fisher)
- NaCl #S3160/65 (Fisher Scientific)
- NP40 #19628 (USB)
- Na deoxycholate #D6750 (Sigma-Aldrich)
- SDS #75746 (Sigma-Aldrich)
- β-mercaptoethanol #M7154 (Sigma-Aldrich)
- glycerol #G/0650/08 (Fisher)
- bromophenol blue #B8026 (Sigma-Aldrich)

- glycine #G/0800/60 (Fisher)
- methanol #M/4000/17 (Fisher)
- radioimmunoprecipitation assay (RIPA) buffer (50 mM of Tris pH8.0, 150 mM of NaCl, 1% NP40, 0.5% Na deoxycholate, 0.1% SDS)
- protease inhibitor cocktail 100x #P8340 (Sigma-Aldrich)
- phosphatase inhibitor cocktail 1+2 #K1012 #K1013 (APExBIO)
- make up working RIPA buffer with 1x protease inhibitors and phosphatase inhibitors
- 2x Laemmli loading buffer (0.125M Tris pH6.8, 4% SDS, 10% β-mercaptoethanol, 20% glycerol, 0.004% bromophenol blue)
- 1x TBS (50mM Tris-HCl pH7.4, 150mM NaCl)
- blocking buffer: 5% semi-skimmed milk, 0.1% Tween 20, TBS
- wash buffer: 0.1% Tween 20, TBS
- transfer buffer (39mM glycine, 48mM Tris, 1.3mM SDS, 20% methanol)
- 10x reservoir buffer (0.25M Tris, 1.9M glycine)
- running buffer: 1x reservoir buffer, 0.1% SDS
- 6-well plates #BC010 3516 (Corning)
- blotting paper #1703966 (Bio-Rad)
- 100ºC heat block (Grant)
- spectrophotometer Ultrospec 2100*pro* (Amersham BioSciences)
- rotator (FALC F205)
- Mini-PROTEAN-Tetra cell system #1658005EDU (Bio-Rad)
- refrigerated centrifuge (Sorvall RT6000B)
- Bio-Rad Powerpac 300 (for running SDS-PAGE gel)
- Bio-Rad Powerpac 200 (for running semi-dry blot)
- Bio-Rad Trans-Blot SD
- ChemiDoc™ XRS+ system (Bio-Rad)
- ImageLab 6.0.1 software (Bio-Rad)

Methods:

- Grow cells to 90% confluent in a 6-well plate
- Wash cells 2x 5ml ice-cold DPBS
- Add 100µl of RIPA buffer to each well. Collect cells with a cell scraper into a 1.5ml Eppendorf tube
- Rotate on a rotator for 30 minutes at 4°C
- centrifuged at 14,000 rpm for 20 minutes at 4°C to pellet debris
- Transfer supernatant containing proteins to a new Eppendorf tube. Store at -80ºC
- Measure protein concentrations using BCA assay and BSA as protein standards with a spectrophotometer
- Prepare equal quantities of protein samples. Make up to a total of 10µl with RIPA buffer
- Add 10µl 2x Laemmli loading buffer to each sample
- heat at 100ºC for 5min
- load 20 µl of each sample onto TGX Stain-Free SDS-PAGE gel, together with protein markers
- Use Powerpac 300 to run samples at 100V for about 20min (until blue dye enter separating gel). Turn up to 200V until blue dye reaches 1cm from gel bottom
- Remove gel to ChemiDoc. Activate gel

- Transfer gel to 100ml transfer buffer for 15min. Wet a PVDF membrane with methanol for 1min and then transfer to 100ml transfer buffer for 15min. Also soak two blotting papers in transfer buffer for 15min
- Transfer proteins from gel to PVDF membrane using semi-dry blot and Powerpac 200 for 1h (start at 12V until current drops to 8mA, turn up to 25V for the remaining time)
- After transfer, use ChemiDoc to capture total protein image in blot
- block the membrane in 100ml blocking buffer for 1hr at RT with vigorous shaking
- Transfer the membrane to a plastic bag containing antibody in blocking buffer and place on a gentle rotating platform in the cold room overnight
- Next day, remove membrane from the bag and wash 3x 5min each with 100ml wash buffer at RT with vigorous shaking
- Transfer the membrane to a plastic bag containing secondary antibody in blocking buffer on a gentle rotating platform for 2h at RT
- wash 3x 5min each with 100ml wash buffer at RT with vigorous shaking. Wash 1x with 100ml TBS with vigorous shaking
- Detect protein bands with ECL substrate
- Capture image using ChemiDot

8.7 Immunocytochemistry

Materials:

- Cultured cells
- C6/36 growth medium: Eagle's minimal essential medium (MEM) #M4655 (Sigma-Aldrich) supplemented with 1x non-essential amino acid (NEAA) #M7145 (Sigma-Aldrich), 10% fetal calf serum (FCS) #16500-064 (Gibco) and 100 U/ml penicillin, and 100mg/ml streptomycin #P0781 (Sigma-Aldrich)
- anti-PHB2 antibody #12295-1-AP (Proteintech)
- rabbit serum #R9133 (Sigma-Aldrich)
- horse serum #H0146 (Sigma-Aldrich)
- anti-rabbit horseradish peroxidase-conjugated secondary antibody #7074 (Cell Signaling Technology)
- SIGMA *FAST*™ DAB (3,3′-diaminobenzidine tetrahydrochloride) #D0426-50SET (Sigma-Aldrich)
- Fluoroshield mounting medium #F6182 (Sigma-Aldrich)
- methanol #M/4000/17 (Fisher) (store at -20°C) use straight from -20°C
- PBS #P4417-100TAB (Sigma-Aldrich) made up one tablet in 200ml milli-Q water; autoclaved
- Triton-X-100 #T9284 (Sigma-Aldrich)
- BSA #A8806 (Sigma-Aldrich)
- H_2O_2 #H1009 (Sigma-Aldrich)
- 0.3% Triton-X-100 /PBS
- 1%H_2O_2/PBS Blocking buffer (1 % horse serum/2 % BSA/PBS/0.03% Triton X-100) NOTE: use the species that is used to raise the secondary antibody; in this case it is horse so horse serum is used as block
- 8-well chamber slides #177437 (Permanox, Nunc)
- CO_2 incubator at 28°C and 5% CO_2 (MiniGalaxy E)

Methods:

- Seed cells at 0.5x10^5 /0.5ml in 8-well chamber slides in growth medium in a 28°C and 5% CO_2 incubator overnight
- Remove medium. Add 0.5ml cold methanol to each well. Put on ice to fix cells for 1h
- wash 1x in PBS. Add 0.5ml 0.3% Triton-X-100 /PBS to each well for 10min
- wash 1x in PBS. Add 0.5ml 1%H_2O_2/PBS for 10min
- wash 2x in PBS, incubate 5min each
- add blocking buffer to wells for 1 h
- Add 1:100 anti-PHB2 antibody or 1:500 control rabbit serum in blocking buffer to wells and incubate overnight in a humidifying chamber at 4°C
- Wash 1x in blocking buffer with a 5min incubation
- Add 1:50 anti-rabbit horseradish peroxidase-conjugated secondary antibody in blocking buffer for 1h at RT in a humidifying chamber
- Wash 3x in blocking buffer, 5min each. Rinse 2x in PBS
- Develop colour using SIGMA *FAST*™ DAB
- Mount in Fluoroshield mounting medium

8.8 Growth curve

Materials:

- Cultured cells
- C6/36 growth medium: Eagle's minimal essential medium (MEM) #M4655 (Sigma-Aldrich) supplemented with 1x non-essential amino acid (NEAA) #M7145 (Sigma-Aldrich), 10% fetal calf serum (FCS) #16500-064 (Gibco) and 100 U/ml penicillin, and 100mg/ml streptomycin #P0781 (Sigma-Aldrich)
- trypsin #T3924 (Sigma-Aldrich)
- haemocytometer (Deubauer chamber)
- 6-well plates (Corning)
- 12-well plates (Corning)
- 28°C and 5% CO_2 incubator (MiniGalaxy E)
- Software: Excel (Microsoft) and Prism (GraphPad)

Methods:

Early growth phase

- Seed cells at 1x10^5/well in growth medium in multiple wells in a 6-well plate and grow in a 28°C and 5% CO_2 incubator overnight
- Trypsinize, count number of cells by a haemocytometer
- Repeat over a course of four days. Count one well per day.
- Analyse data using Excel (Microsoft) and Prism (GraphPad)
- Plot cell growth as a percentage of the seeding density

Prolonged growth

- Seed cells at 1-6x10^4/well in growth medium in multiple wells of a 12-well plate and grow in a 28°C and 5% CO_2 incubator overnight
- Trypsinize, count number of cells by a haemocytometer
- Repeat over a course of 12 days. Count one well per day.
- Analyse data using Excel (Microsoft) and Prism (GraphPad)
- Plot cell growth as a percentage of the seeding density

8.9 XTT viability assay

Materials:

- Cultured cells
- C6/36 complete medium: C6/36 growth medium: Eagle's minimal essential medium (MEM) #M4655 (Sigma-Aldrich) supplemented with 1x non-essential amino acid (NEAA) #M7145 (Sigma-Aldrich), 10% fetal calf serum (FCS) #16500-064 (Gibco) and 100 U/ml penicillin, and 100mg/ml streptomycin #P0781 (Sigma-Aldrich)
- C6/36 serum-free medium: C6/36 growth medium: Eagle's minimal essential medium (MEM) #M4655 (Sigma-Aldrich) supplemented with 1x non-essential amino acid (NEAA) #M7145 (Sigma-Aldrich), and 100 U/ml penicillin, and 100mg/ml streptomycin #P0781 (Sigma-Aldrich)
- XTT Cell Viability Assay Kit #30007 (Biotium). Make up XTT working solution with XTT solution and activation reagent PMS before use
- 96-well plates (Corning)
- multichannel pipette
- 28°C/5% CO_2 incubator (MiniGalaxy E)
- plate reader (Bio-Tek Synergy HT)
- Software: Excel (Microsoft) and Prism (GraphPad)

Methods:

- Day 0: seed cells at 60,000 per well of a 96-well plate in 100μl complete medium. Seed multiple plates enough to measure for the duration of the test. Put in a 28°C/5% CO_2 incubator overnight
- Day 1: add 25μl XTT working solution to each well of one plate using a multichannel pipette. Put in a 28°C/5% CO_2 incubator. Measure absorbance at 450nm and reference 650nm 1-24h after XTT addition using a plate reader. Choose one time-point for analysis. Subtract background 650nm readings from 450nm readings. Use Day1 reading to normalize the starting cell density for each cell types
- replace the medium in all the other plates with either 100μl serum-free medium or complete medium (control).
- Day 2: add 25μl XTT working solution to each well of one plate using a multichannel pipette. Put in a 28°C/5% CO_2 incubator. Measure absorbance at 450nm and reference 650nm 1-24h after XTT addition using a plate reader.
- Repeat for Day 3 using a new plate for each day of measurement.
- Analyse data using Excel and Prism
- Plot cell growth as a percentage of to the parental cells in complete medium of the same day
